# Determinants for worsening in systemic autoimmune rheumatic disease-associated interstitial lung disease: a systematic review and meta-analysis of cohort studies

**DOI:** 10.3389/fmed.2024.1465753

**Published:** 2024-11-27

**Authors:** Jiaheng Yao, Jun Wang, Luhan Guo, Peipei Su, Jiansheng Li, Bin Li

**Affiliations:** ^1^Department of Respiratory and Critical Care Medicine, The First Affiliated Hospital of Henan University of Chinese Medicine, Zhengzhou, China; ^2^The First Clinical Medical College of Henan University of Chinese Medicine, Zhengzhou, China; ^3^Collaborative Innovation Center for Chinese Medicine and Respiratory Diseases Co-Constructed by Henan Province & Education Ministry of P.R. China, Henan University of Chinese Medicine, Zhengzhou, China; ^4^Department of Rheumatology, The First Affiliated Hospital of Henan University of Chinese Medicine, Zhengzhou, China

**Keywords:** systemic autoimmune rheumatic disease, interstitial lung disease, progression, acute exacerbation, rapidly progressive interstitial lung disease, risk factors, systematic review, meta-analysis

## Abstract

**Background:**

To identify risk factors for progression, acute exacerbation (AE), and the development of rapidly progressive interstitial lung disease (RP-ILD) in Systemic autoimmune rheumatic disease-associated interstitial lung disease (SARD-ILD).

**Methods:**

We systematically searched PubMed, EMBASE, Scopus, the Cochrane Library, and Web of Science databases to identify eligible cohort studies up until January 01, 2024. Two reviewers independently screened the literature and extracted data. We employed the Newcastle-Ottawa Scale (NOS) to assess study quality and performed meta-analyses using STATA software.

**Results:**

This review included 50 studies. For progression, 28 studies were included, four significant risk factors were identified: male (OR = 1.97, 95% CI 1.26–3.08, *p* < 0.001), UIP patterns on HRCT (OR = 1.94, 95% CI 1.48–2.54, *p* < 0.001), extensive lung involvement (OR = 2.15, 95% CI 1.66–2.80, *p* < 0.001), and age (OR = 1.07, 95% CI 1.05–1.10, *p* < 0.001); and 11 potential risk factors. Seven studies were included in AE, and three potential risk factors were highlighted: FVC, UIP patterns on HRCT, and smoking history. In RP-ILD, 15 studies were included. Three risk factors were determined: High CRP (OR = 2.45, 95% CI 1.87–3.21, *p* < 0.001), Ro-52 positivity (OR = 5.35, 95% CI 3.46–8.29, *p* < 0.001), and MDA5 antibodies (OR = 2.09, 95% CI 1.47–2.95, *p* < 0.001); along with 10 potential risk factors.

**Conclusion:**

Our meta-analysis identified male sex, UIP pattern on HRCT, extensive lung involvement, and advanced age as significant risk factors for the progression of SARD-ILD. High CRP, Ro-52 positivity, and MDA5 antibodies were significant risk factors for developing of RP-ILD in patients with IIM. We also discovered several potential risk factors that may be associated with the progression of SARD-ILD and acute exacerbation, as well as the occurrence of RP-ILD in IIM patients.

**Systematic review registration:**

https://www.crd.york.ac.uk/prospero/.

## 1 Introduction

Interstitial lung diseases (ILDs) refer to a group of disorders characterized by chronic inflammation and fibrosis of the alveolar walls, lung interstitium, and pulmonary vasculature. Among the various causes of ILDs, systemic autoimmune rheumatic diseases (SARDs) are common etiologies (1, 2). Although the prevalence of ILD varies among different types of SARDs (3)—approximately 11% in rheumatoid arthritis (RA), 47% in systemic sclerosis (SSc), 41% in idiopathic inflammatory myopathies (IIM), 17% in primary Sjögren’s syndrome (pSS), 56% in mixed connective tissue disease (MCTD), and 6% in systemic lupus erythematosus (SLE)—SARD-ILD shares significant similarities with idiopathic interstitial pneumonia (IIP) in terms of imaging, pulmonary function, pathology, and clinical presentation (4–6). For example, on high-resolution computed tomography (HRCT), a portion of SARD-ILD cases present with a usual interstitial pneumonia (UIP) pattern. Pulmonary function tests (PFTs) often reveal restrictive ventilatory defects, notably decreased forced vital capacity (FVC) and diffusing capacity for carbon monoxide (DLCO). Pathologically and clinically, interstitial inflammation or fibrosis leads to impaired gas exchange and exertional dyspnea. Furthermore, some IIP patients may even exhibit autoimmune characteristics. Therefore, despite the complex etiology of SARD-ILD, it shares significant similarities with IIP. In some SARD-ILD patients, despite treatment, disease worsening is inevitable. These patients often have poor clinical control and a shorter survival time (7–9).

As research continues to advance and the number of cases increases, it has been observed that SARD-ILD closely resembles the worsening forms of IIP, primarily manifesting in three types: progression, acute exacerbation (AE), and rapidly progressive ILD (RP-ILD). Progression refers to the gradual deterioration of interstitial lung disease, with different types of ILD exhibiting various patterns of progression; some may be characterized predominantly by interstitial inflammation, some may show predominant interstitial fibrosis, but all are associated with radiographic progression or a decline in pulmonary function. AE, in contrast, involves a sudden worsening of the condition over a short period, typically presenting as an acute decline in respiratory function, accompanied by new extensive pulmonary infiltrates on imaging, although this does not necessarily correlate with worsening of pre-existing fibrosis (10). RP-ILD, commonly seen in certain IIM-ILD patients, is characterized by rapid deterioration within weeks to months, leading to the onset of dyspnea, with mechanisms often involving acute inflammation and rapid fibrosis mediated by autoimmunity (11). These three forms of exacerbation in SARD-ILD directly impact patients’ quality of life. Therefore, understanding the risk factors and early identification of high-risk patients is crucial for alleviating economic burdens, improving quality of life, and extending survival.

Numerous researchers have shifted focus to risk factors for progression, AE, and RP-ILD, yet knowledge in this area still needs to be made available. Even within the same SARD type, conclusions on risk factors by different researchers vary significantly. For instance, widely recognized factors such as male gender, UIP pattern, and smoking history have shown considerable discrepancies across studies, lacking validation through larger samples and more extensive data. Based on this, we conducted a systematic review and meta-analysis of cohort studies regarding risk factors for progression, AE, and RP-ILD in SARD-ILD, aiming to clarify these risk factors with a larger sample and more data. By analyzing results that could potentially be risk factors, this study provides direction for future research on risk factors, offers a solid evidence base for the clinical management of SARD-ILD, and lays the groundwork for devising more precise risk assessment and management strategies, thereby improving patient outcomes, enhancing quality of life, and reducing economic burdens.

## 2 Manuscript formatting

### 2.1 Methods

This study was reported following the Meta-analysis of Observational Studies in Epidemiology (MOOSE) (12) and the Preferred Reporting Items for Systematic Reviews and Meta-Analyses (PRISMA) (13) guidelines. The protocol has been preregistered on PROSPERO (CRD42024495275).

#### 2.1.1 Data and search strategy

We systematically searched the PubMed, EMBASE, Scopus, Cochrane Library, and Web of Science databases without language restrictions from their inception to January 01, 2024. Databases were searched and data abstracted by two authors working independently. We used subject headings and text words related to the study population to finish the search. The reference lists of eligible studies and relevant review articles were also hand-searched to find additional reports. The detailed search strategy is provided in Supplementary material.

#### 2.1.2 Study selection and data extraction

The included studies were required to meet the following criteria: (1) Population (P): Adults diagnosed with SARD-ILD; (2) Intervention (I) and Comparison (C): Not applicable, as the focus is on observational metrics without direct comparisons; (3) Outcomes (O): Identification of predictors for progression, AE, or RP-ILD, quantified through adjusted odds ratios (OR), relative risks (RR), or hazard ratios (HR); (4) Study Design (S): Cohort studies that enrolled at least 10 patients, focusing on progression, AE, or RP-ILD as the primary outcome. It is noteworthy that the outcomes assessed in this study did not include mortality. Exclusion criteria were as follows: (1) Repeatedly published data; (2) Literature with incomplete data or lacking target indicators; (3) Review articles, letters, conference abstracts, and editorials.

The definition of progression was based on the guidelines for PPF (7). Although there are no authoritative guidelines for the definitions of AE and RP-ILD, we adopted widely accepted criteria (14, 15). It is important to note that the studies we included did not use a uniform definition of disease progression. Therefore, we conducted a detailed review of the progression definitions to ensure that all study reports adhered to the criteria mentioned above. For example, some studies applied the more stringent criterion of a 10% decline in FVC, but all met the 5% decline threshold.

Two authors independently extracted data from the included articles based on a predefined data extraction form. Extracted data included the first author’s name, year of publication, study location, study design, sample size, demographic features of the subjects, outcomes, potential prognostic factors, and their effect estimates.

#### 2.1.3 Risk of bias assessment

The methodological quality of each cohort was assessed using the 9-point Newcastle–Ottawa scale (NOS) (16). Studies were deemed high quality when the score was at 9 points, acceptable quality when the score was 6 to 8 points, and low quality when the score was ≤5 points.

#### 2.1.4 Data synthesis and statistical analysis

All of the statistical analyses were performed using STATA V.18.0 software. The adjusted RR/OR/HR and 95% CI from each study were used to assess the risk of progression/AE/RP-ILD in SARD-ILD. The *I*^2^ value was used to evaluate heterogeneity. Generally, we used the fixed-effects model to analyse substantial homogeneous trials (I^2^ ≤ 50%, *p* > 0.05). When statistical heterogeneity existed (*I*^2^ > 50%, *p* ≤ 0.05), we used a random-effects model followed by performed sensitivity analyse to verify the robustness of the overall results and explore the sources of heterogeneity, which were carried out by gradually removing studies (17). Forest plots were used to display the results from the individual studies and the pooled estimates. The potential for publication bias was evaluated by funnel plots and Egger’s test if 5 or more studies were available for meta-analysis (18). If publication bias was present, the trim and fill method was used to verify the asymmetric funnel plot. The subgroup analysis of SARD was conducted in cases with high heterogeneity and an adequate number of studies were included. In situations where the number of studies was insufficient to group according to SARD type, alternative criteria such as different data on the inclusion of risk factors were utilized. *p* ≤ 0.05 were considered statistically significant.

## 3 Results

### 3.1 Study identification

**Figure 1:**
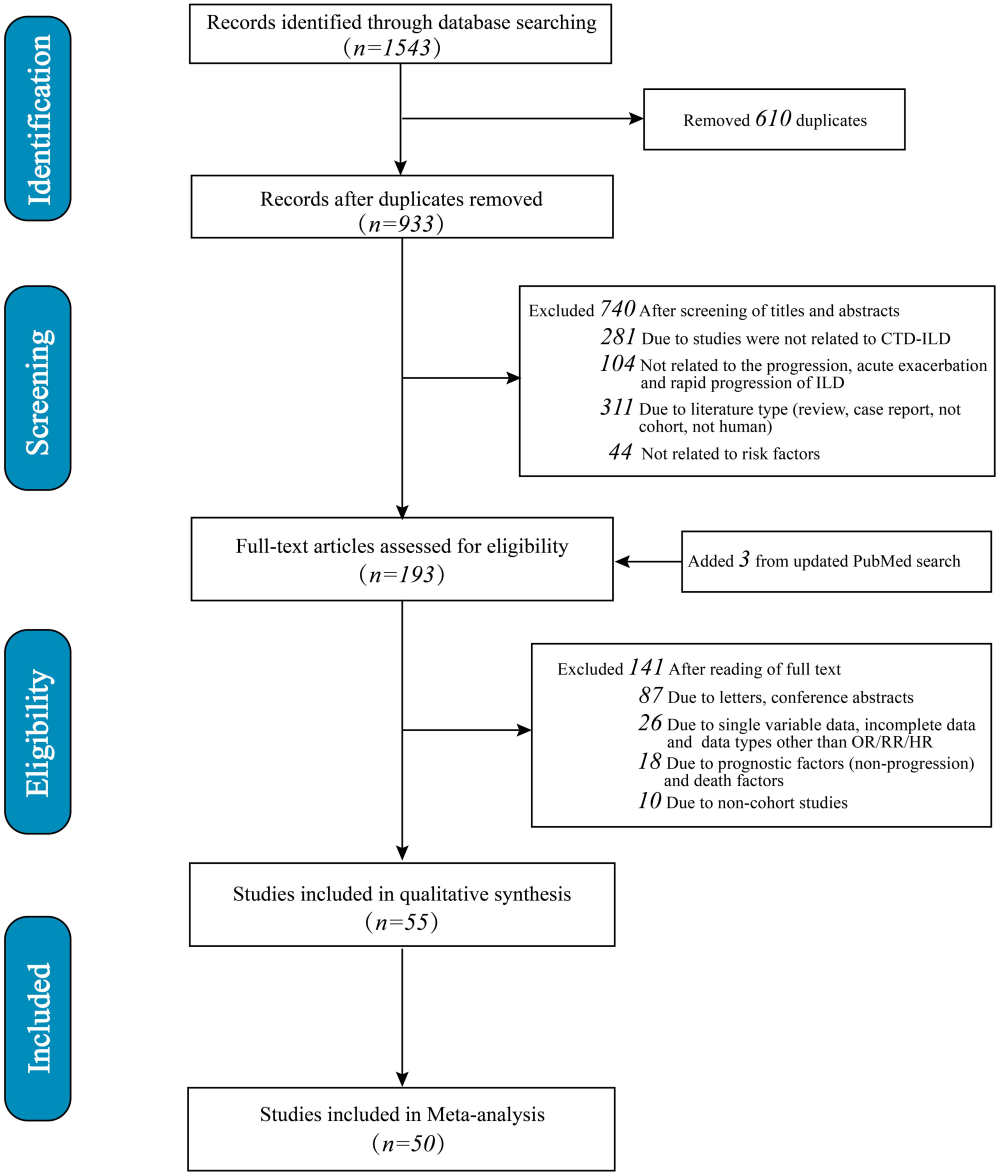
Flow diagram showing literature search and results.

A total of 1,543 studies were identified. Initially, 610 duplicates were eliminated, followed by the exclusion of 740 studies after reviewing titles and abstracts (281 were irrelevant to SARD-ILD; 104 were unrelated to progression, acute exacerbation, or rapid progression; 311 did not match the study type, such as reviews or case reports; 44 were unrelated to risk factors). Subsequently, 141 articles were excluded after full-text review (87 due to being letters or conference abstracts; 26 lacked adjusted multivariate risk factor data or did not report OR/RR/HR; 18 were excluded due to prognostic factors unrelated to progression or mortality factors; 10 were non-cohort studies). An additional three articles were identified through an updated search in PubMed. Ultimately, 55 studies met the criteria for systematic review and meta-analysis, with 50 studies involving multivariate adjustments included in the quantitative synthesis (Figure 1). Only risk factors that were reported in at least two studies were eligible for inclusion in the meta-analysis. The final five studies were excluded because the risk factors identified in these studies were not addressed in other studies, and there was insufficient data for inclusion in the meta-analysis.

#### 3.1.1 Study characteristics

The analysis incorporated 55 cohort studies (6 prospective cohorts, 49 retrospective cohorts) (19–73), including 32 in the progression group, 8 in the AE group, and 15 in the RP-ILD group, encompassing 7,948 patients. The majority of the studies originated from Asia (China *n* = 28, Japan *n* = 9, Korea *n* = 6), followed by Europe (Spain *n* = 3, France *n* = 2, Italy *n* = 1, Norway *n* = 1, the Netherlands *n* = 1), and included four international multicenter studies. The progression group comprised studies on Rheumatoid Arthritis (RA) (*n* = 10), Systemic Sclerosis (SSc) (*n* = 7), Idiopathic Inflammatory Myopathies (IIM) (*n* = 5), Primary Sjögren’s Syndrome (PSS) (*n* = 4), Mixed Connective Tissue Disease (MCTD) (*n* = 2), and general CTDs (*n* = 4); the AE group mostly included RA (*n* = 5), CTDs (*n* = 2), and SSc (*n* = 1); all 15 RP-ILD group studies were on IIM. In this systematic review, 78 risk factors were involved in the progression group, 24 in the AE group, and 38 in the RP-ILD group. The meta-analysis included 50 studies in total: 28 in the progression group (19–26, 28–40, 42–44, 46, 47, 49, 50), 7 in the AE group (65, 67–72), and 15 in the RP-ILD group (51–64, 73). Of these, 28 risk factors within the progression group, 6 within the AE group, and 15 within the RP-ILD group were analyzed. The risk factors not included in the meta-analysis were due to the involvement of only a single study. Detailed characteristics are presented in Supplementary Table S1.

#### 3.1.2 Quality evaluation of the included studies

The methodological quality of the 55 included studies was assessed using the NOS, with 3 studies categorized as medium quality (scoring 6) and 52 as high quality (4 studies scored 7, 32 studies scored 8, and 16 studies scored 9), resulting in an average score of 8.1. This indicates a high quality of the included studies with a low risk of bias. The specific scores for all studies are available in Supplementary Table S2.

#### 3.1.3 Risk factors for SARD-ILD progression

**Figure 2:**
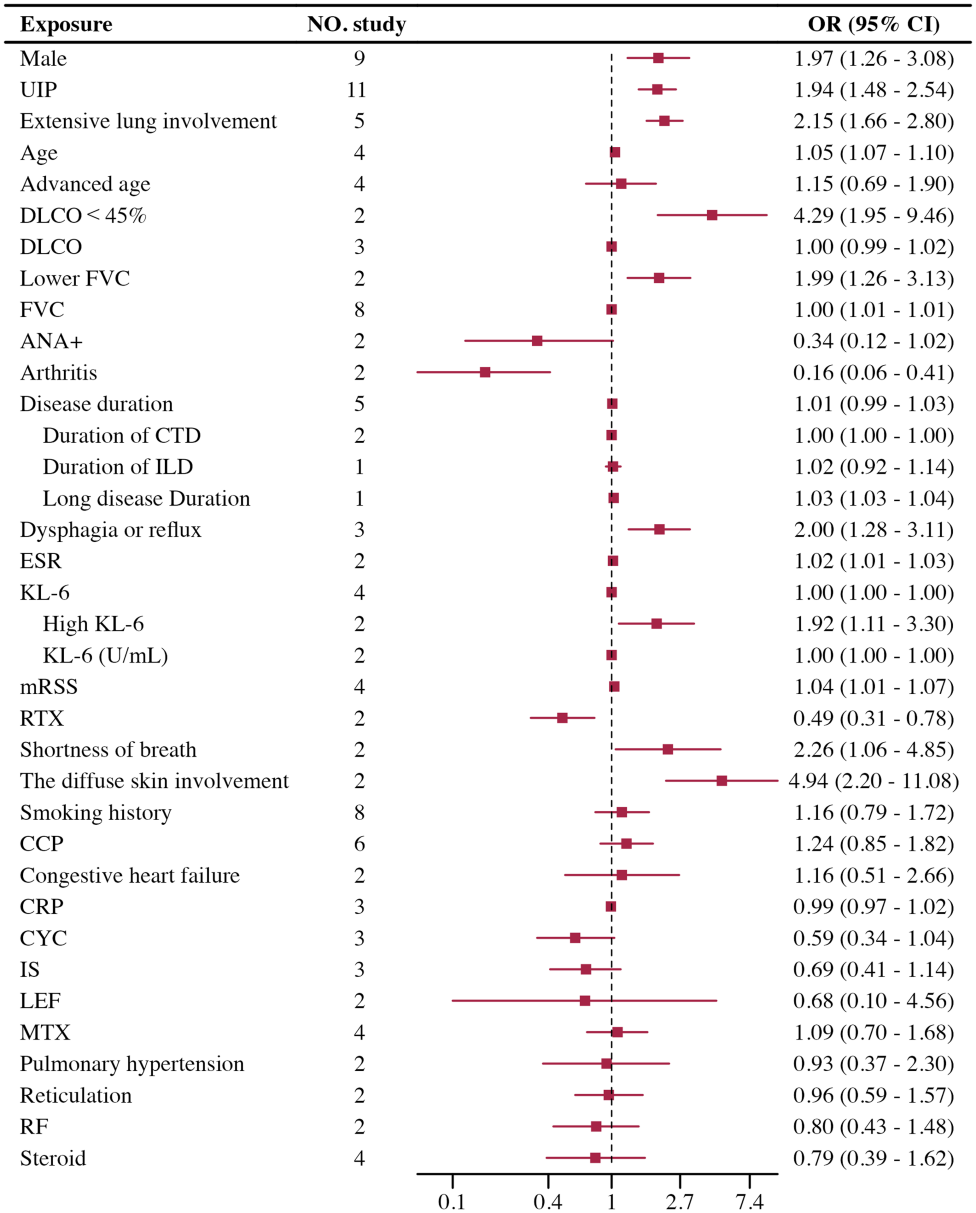
Forest plot of risk factors for progression of SARD-ILD.

A total of 28 risk factors associated with the progression of SARD-ILD were included in the meta-analysis. There were 15 statistically significant factors: male, UIP patterns on HRCT, extensive lung involvement, age, low FVC, low DLCO, positive Antinuclear Antibodies (ANA), elevated Erythrocyte Sedimentation Rate (ESR), diffuse skin involvement, Modified Rodnan Skin Score (mRSS), Krebs von den Lungen-6 (KL-6), Rituximab (RTX), arthritis, dysphagia or reflux, and shortness of breath.13 factors were not statistically significant: smoking history, disease duration, Cyclic Citrullinated Peptide (CCP), C-reactive protein (CRP), Rheumatoid Factor (RF), reticulation on HRCT, pulmonary hypertension, congestive heart failure, Cyclophosphamide (CYC), Immunosuppressive (IS), Leflunomide (LEF), Methotrexate (MTX), and steroids. The results are summarized in Figure 2.

Significant heterogeneity was observed only in the male, FVC, DLCO, smoking history, steroid, and LEF groups, with other groups showing low heterogeneity. Heterogeneity testing results are shown in Table 1. Male: High heterogeneity. Removal of the Vadillo et al. (33) study significantly reduced heterogeneity, with no significant change in the adjusted results. Steroid: High heterogeneity was observed. Removal of the Chiu et al. (35) study significantly reduced heterogeneity, and the results remained statistically significant. FVC: Subgrouping by data type into two groups: Low FVC as a dichotomous variable (*n* = 2) and FVC as a continuous variable (*n* = 8), showed persistent high heterogeneity in the FVC group but significantly reduced in the Low FVC group. The Low FVC group was statistically significant, while the FVC group showed no significant differences. DLCO: Subgrouping by data type into Low DLCO as a dichotomous variable (*n* = 2) and DLCO as a continuous variable (*n* = 3), significantly reduced heterogeneity in both subgroups. The Low DLCO group showed statistical significance, while the DLCO group showed no significant differences. Smoking history: Subgroup analysis by SARD type significantly reduced heterogeneity, indicating statistical significance only in the undifferentiated SARD group, with no significance in others. LEF: High heterogeneity, with only two studies involved, making further analysis difficult.

**Table 1:** Summary of results after adjustments for risk factors with high heterogeneity.

Type	Risk factor	Heterogeneity (before adjustment)	Heterogeneity (before adjustment)	Result (before adjustment)
Progression	Male	*I*^2^ = 51.32%, *p* = 0.04	*I*^2^ = 0.82%, *p* = 0.42	OR = 2.32, 95% CI 1.65–3.27, *p* < 0.001
	FVC	*I*^2^ = 80.29%, *p* < 0.001		
	Low FVC	Subgroup	*I*^2^ = 43.98%, *p* = 0.18	OR = 1.99, 95% CI 1.26–3.13, *p* < 0.001
	FVC (continuous variable)	Subgroup	*I*^2^ = 80.13%, *p* < 0.001	OR = 0.99, 95% CI 0.97–1.01, *p* = 0.18
	DLCO	*I*^2^ = 75.02%, *p* < 0.001		
	Lower DLCO	Subgroup	*I*^2^ = 29.8%, *p* = 0.23	OR = 4.50, 95% CI 1.71–11.83, *p* < 0.001
	DLCO (continuous variable)	Subgroup	*I*^2^ = 0%, *p* = 0.46	OR = 1.00, 95% CI 0.99–1.02, *p* = 0.82
	Smoking history	*I*^2^ = 57.64%, *p* = 0.02	NA	NA
	Steroid	*I*^2^ = 70.29%, *p* = 0.02	*I*^2^ = 0%, *p* = 0.70	OR = 0.57, 95% CI 0.38–0.86, *p* = 0.01
	LEF	*I*^2^ = 91.16%, *p* < 0.001	NA	NA
AE	FVC	*I*^2^ = 0%, *p* = 0.37	NA	NA
	UIP	*I*^2^ = 0%, *p* = 0.65	NA	NA
	Smoking history	*I*^2^ = 0%, *p* = 0.96	NA	NA
	Age	*I*^2^ = 40.69%, *p* = 0.17	NA	NA
	MTX	*I*^2^ = 0%, *p* = 0.86	NA	NA
	Steroid	*I*^2^ = 77.76%, *p* = 0.03	NA	NA
RP-ILD	Disease duration	*I*^2^ = 91.88%, *p* < 0.001		
	Disease duration (continuous variable)	Subgroup	*I*^2^ = 8.76%, *p* = 0.3	OR = 0.76, 95% CI 0.67–0.87, *p* < 0.001
	Short disease duration	Subgroup	*I*^2^ = 0, *p* = 0.61	OR = 3.40, 95% CI 2.37–4.86, *p* < 0.001
	CRP	*I*^2^ = 82.26%, *p* < 0.001		
	High CRP	Subgroup	*I*^2^ = 0, *p* = 0.76	OR = 2.45, 95% CI 1.87–3.21, *p* < 0.001
	CRP (continuous variable)	Subgroup	*I*^2^ = 29.43%, *p* = 0.24	OR = 1.07, 95% CI 1.02–1.12, *p* < 0.001
	SF	*I*^2^ = 65.15%, *p* = 0.01		
	High SF	Subgroup	*I*^2^ = 0, *p* = 0.95	OR = 1.86, 95% CI 1.17–2.69, *p* = 0.01
	SF (continuous variable)	Subgroup	*I*^2^ = 60.10%, *p* = 0.06	OR = 1.00, 95% CI 1.00–1.00, *p* = 0.05
	Lymphocytes	*I*^2^ = 64.06%, *p* = 0.04		
	Low lymphocytes	Subgroup	*I*^2^ = 0, *p* = 0.44	OR = 2.42, 95% CI 1.28–4.58, *p* = 0.01
	Lymphocytes (continuous variable)	Subgroup	NA	NA

NA: Not available, Due to the limited number of included studies, heterogeneity was not addressed. FVC, DLCO, disease duration, CRP, SF, and lymphocytes were divided into two subgroups for analysis, with the results presented in the table.

It should be noted that the thresholds for defining low FVC, low DLCO, and advanced age as dichotomous variables varied among the studies included. Specifically, low FVC was defined in two studies as below 70 and 80%, respectively. For low DLCO, both studies used a threshold of less than 45%. Among the four studies assessing age, three defined advanced age as over 60 years, while one used a threshold of over 65 years.

For risk factors included in five or more studies, sensitivity analysis, Egger’s test, and the trim-and-fill method suggest that male, UIP, extensive lung involvement, and age are credible risk factors for SARD-ILD progression. Male, Extensive lung involvement: Sensitivity analysis using the leave-one-out method showed stable results. Egger’s test indicated no significant publication bias, and after applying the trim-and-fill method, bias did not substantially influence the results, suggesting high credibility that both male gender and extensive lung involvement are significant risk factors for progression. UIP patterns on HRCT: Sensitivity analysis showed stable results. Egger’s test indicated significant publication bias, but the trim-and-fill method adjusted the results minimally, indicating that UIP patterns are a highly credible risk factor for progression. Age: Eight studies were included, divided into two subgroups based on data type: age as a dichotomous variable (*n* = 4) and age as a continuous variable (*n* = 4). Only sensitivity analysis was performed due to the nature of the data. Results from both subgroups showed stability, with the age group showing statistical significance. For other risk factors that included fewer than five studies (or subgroups with fewer than five studies), sensitivity analysis and other tests were not performed. Detailed information is shown in Table 2.

**Table 2:** Results after sensitivity analysis, egger’s test, and trim-and-fill method adjustment.

Type	Risk factor	Sensitivity analysis	Egger’s test	Result after trim-and-fill method
Progression	Male	Stable	*p* = 0.103	OR = 1.71, 95% CI 1.12–2.61
	UIP patterns on HRCT	Stable	*p* = 0.009	OR = 1.63, 95% CI 1.27–2.09
	Extensive lung involvement	Stable	*p* = 0.394	OR = 2.09, 95% CI 1.62–2.70
	Age	Stable	*p* = 0.345	OR = 1.07, 95% CI 1.05–1.10
RP-ILD	High CRP	Stable	*p* = 0.807	OR = 2.45, 95% CI 1.87–3.21
	Ro-52	Stable	*p* = 0.183	OR = 4.57, 95% CI 3.05–6.85
	MDA5	Stable	*p* = 0.017	OR = 1.73, 95% CI 1.18–2.55

#### 3.1.4 Risk factors for AE in SARD-ILD

**Figure 3:**
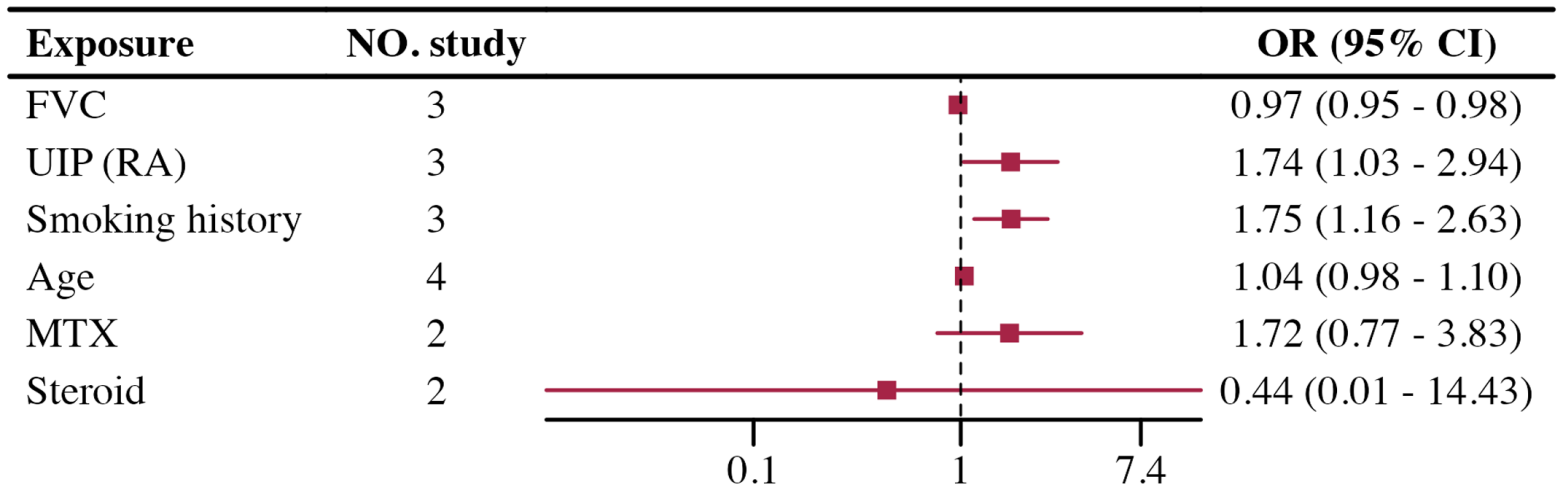
Forest plot of risk factors for AE of SARD-ILD.

Few studies involved multivariate adjustments for acute exacerbation, all with less than five studies. The analysis results for FVC, UIP, and smoking history showed low heterogeneity and were statistically significant. However, analysis of age, MTX, and steroids showed high heterogeneity in the groups, with no statistically significant differences found. Due to the small number of studies included, further analysis such as sensitivity analysis was challenging. Details are provided in Figure 3. Heterogeneity testing results are shown in Table 1.

#### 3.1.5 Risk factors for RP-ILD

**Figure 4:**
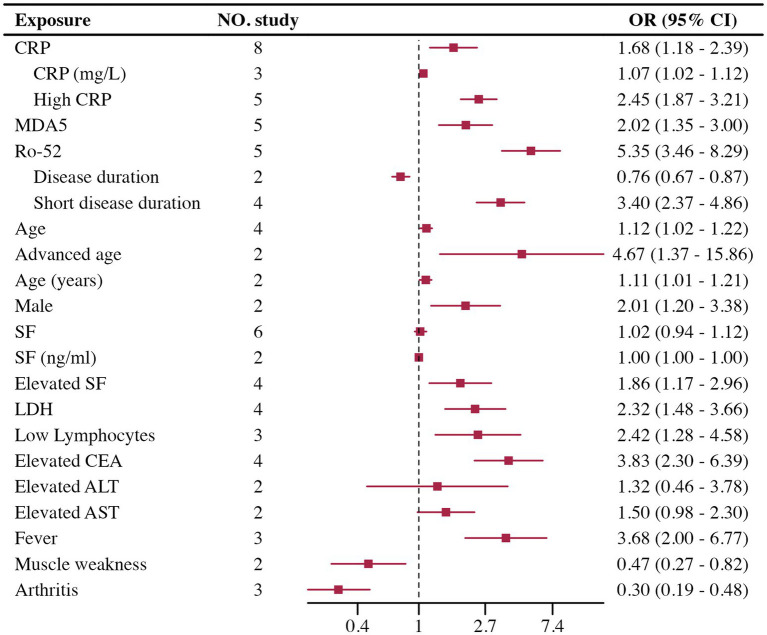
Forest plot of risk factors for RP-ILD.

The Meta-analysis included 15 RP-ILD related risk factors. Thirteen factors were statistically significant: short disease duration, CRP, Ro-52 positivity, MDA5 antibodies, age, male, lymphocytes, Lactate Dehydrogenase (LDH), Serum Ferritin (SF), Carcinoembryonic Antigen (CEA), fever, arthritis, and muscle weakness; only Aspartate Aminotransferase (AST) and Alanine Aminotransferase (ALT) were not statistically significant. Results are summarized in Figure 4.

Heterogeneity testing indicated high heterogeneity for disease duration, CRP, SF, and lymphocytes. Heterogeneity testing results are shown in Table 1. Disease duration: High heterogeneity was observed. It was divided into continuous variable groups for disease duration (*n* = 2) and short disease duration as a dichotomous variable (*n* = 4), with both subgroups showing insignificant heterogeneity. Both groups indicated that short disease duration is a risk factor for RP-ILD. CRP: High heterogeneity was reduced when divided by data type into high CRP as a dichotomous variable (*n* = 5) and CRP as a continuous variable (*n* = 3), with both showing low heterogeneity. Results showed significant differences for high CRP and CRP, suggesting high CRP is a risk factor for RP-ILD. SF: High heterogeneity was reduced when divided by data type into high SF as a dichotomous variable (*n* = 4) and SF as a continuous variable (*n* = 2), with both showing low heterogeneity. Results showed significant differences for high SF and SF, suggesting high SF is a risk factor for RP-ILD. Lymphocytes: High heterogeneity was reduced when divided by data type into low lymphocytes as a dichotomous variable (*n* = 3) and lymphocytes as a continuous variable (*n* = 1), with low heterogeneity in the low lymphocytes group. Results showed significant differences for low lymphocytes and lymphocytes, suggesting low lymphocytes are a risk factor for RP-ILD.

In the RP-ILD group, definitions for short disease duration varied: three studies defined it as ILD diagnosed within less than 3 months, while one study used a two-month threshold. For high CRP, two studies set the cutoff at greater than 5 mg/L, another two studies used 8 mg/L, and one study used 13 mg/L. Among the studies assessing high SF levels, three provided specific thresholds (2,000 ng/mL, 336.2 ng/mL, and 823 ng/mL), while one study classified high SF based on abnormal levels without specifying a cutoff. In the low lymphocyte group, definitions varied across three studies, with two studies using thresholds of less than 500 and 740, respectively, and one study not specifying a definition.

Sensitivity analysis, Egger’s test, and the trim-and-fill method were performed for risk factors included in five or more studies, suggesting that high CRP, Ro-52 positivity, and MDA5 antibody levels are credible risk factors for RP-ILD in patients with IIM. High CRP, Ro-52, and MDA5 all demonstrated stable results in sensitivity analysis. For high CRP and Ro-52, Egger’s test showed no significant publication bias, and the trim-and-fill method indicated no need for adjustment, suggesting high credibility that both high CRP and Ro-52 positivity are significant risk factors. For MDA5, although Egger’s test showed significant publication bias, the trim-and-fill method indicated no change in the results after adjustment, suggesting high credibility that the MDA5 antibody is a significant risk factor for RP-ILD. Other risk factors included fewer than five studies (or fewer than five studies after subgrouping, and therefore, sensitivity analysis and other tests were not conducted). Detailed information is shown in Table 2.

#### 3.1.6 Risk factors not included in the quantitative synthesis

This study extracted all risk factors from the included literature, but not all data were suitable for quantitative analysis. For data insufficient for quantitative analysis, we compiled and organized them for reference: a total of 91 risk factors, including 50 in the progression group, 18 in the AE group, and 23 in the RP-ILD group (Supplementary Table S3).

## 4 Discussion

In this systematic review and meta-analysis, we synthesized data from 50 cohort studies to identify risk factors associated with the progression, AE, and RP-ILD in SARD-ILD. The design of cohort studies provided our analysis with a robust capacity for causal inference. An average bias risk score of 8.1 indicates the high methodological quality of the included studies, enhancing the credibility of our findings. Furthermore, all studies employed multivariate adjusted data, effectively eliminating potential confounders. Sensitivity analysis was conducted to explore the impact of individual studies on the overall effect size. For data derived from more than five studies, Egger’s test was used to assess publication bias, and the trim-and-fill method was employed to evaluate data reliability. The aim was to identify and explore risk factors associated with the progression, AE, and RP-ILD in SARD-ILD.

### 4.1 Progression

Our analysis confirmed four relatively certain risk factors: male gender, UIP pattern, extensive lung involvement, and advanced age. Previous clinical studies on progressive pulmonary fibrosis (74) and systematic reviews on scleroderma (75) have corroborated our findings, indicating that male gender, advanced age, and the extent of lung involvement are independent risk factors for disease progression. The association of male gender with disease progression is not limited to idiopathic pulmonary fibrosis (IPF) but is also applicable to SARD-ILD. This may be due to differences in immune responses, environmental exposures, genetic susceptibility, and hormonal influences between men and women (76). Advanced age is often associated with a decline in immune function, particularly in the capacity to combat chronic inflammation and fibrosis, which may explain the more rapid disease progression observed in older patients. Additionally, older patients tend to have reduced physiological reserves, leading to decreased compensatory ability when facing the progression of lung fibrosis. Lower baseline lung function in elderly patients means that even minor exacerbations of fibrosis may result in significant clinical deterioration, further emphasizing the need for early identification and treatment in elderly SARD-ILD patients to prevent irreversible lung damage.

Extensive lung involvement suggests a greater fibrotic burden, which is typically associated with severe impairment of lung function and may accelerate disease progression. The broader the extent of lung fibrosis, the greater the damage to the alveoli, leading to more severe gas exchange impairment and rapid decline in respiratory function. This is consistent with findings from other studies, indicating that extensive lung involvement is a key predictor of poor prognosis. Therefore, early monitoring and more intensive therapeutic interventions for patients with extensive lung involvement may help delay disease progression and improve survival rates. Additionally, the studies included in this research defined extensive lung involvement in varying ways. For instance, some studies utilized HRCT scoring, while others based their criteria on the extent of lesions. Therefore, further research is needed to standardize these definitions and to enhance our understanding of extensive lung involvement. The UIP pattern serves as a radiological risk factor in SARD-ILD patients, correlating with poorer prognosis and higher mortality (77). This phenomenon may be due to the tendency of UIP patients to develop irreversible fibrotic lesions, which continuously damage lung tissue and impair gas exchange. Such irreversible fibrotic changes are also common in IPF, suggesting that UIP patients may require more aggressive treatment strategies. Furthermore, the presence of a UIP pattern may indicate that patients are already in the late stages of fibrotic disease, highlighting the critical importance of early diagnosis and treatment to mitigate the decline in lung function and risk of mortality.

Among the 11 factors potentially related to disease progression, low DLCO and low FVC warrant special mention. Although many researchers consider them as predictive factors, our study did not provide reliable confirmation, merely showing differences between the low DLCO and low FVC groups. We speculate that this discrepancy may arise from the majority of studies analyzing FVC as a continuous variable. In our study, low FVC and low DLCO were identified as risk factors for disease progression. However, the number of studies included was limited, and their definitions varied. Future research should aim to establish clear thresholds and consider stratifying populations to better identify high-risk groups [e.g., DLCO <40%, 40–59%, 60–79%, DLCO >80% (78)]. Other risk factors have also been suggested by previous researchers, but our analysis was limited due to the small number of studies available for further evaluation. Regarding the 13 factors that did not show significant statistical significance, the most controversial is the MTX group (79), where the impact of MTX use on ILD progression remains debated. In our study, four studies focused on RA-ILD showed no statistical significance, suggesting that at least in RA-ILD, progression is not related to MTX use. This supports our conclusion but does not demonstrate a protective effect, necessitating further clinical cohort analysis.

### 4.2 Acute exacerbation

In a previous unadjusted univariate systematic review and meta-analysis of RA-ILD acute exacerbation (80), the results (male gender, smoking history, low FVC, UIP patterns on HRCT) were consistent with our findings. Unfortunately, the limited number of relevant studies included did not allow for further validation of the results’ credibility. Notably, the use of MTX and steroids did not demonstrate significant statistical significance in our study. The treatment options for RA-ILD have become complicated due to the potential pulmonary toxicity of many disease-modifying anti-rheumatic drugs (DMARDs) and their uncertain efficacy on lung disease. While MTX and steroids are standard treatments for RA, their relationship with acute exacerbation remains controversial (81–83). Our results indicate that neither MTX nor steroids significantly increased or decreased the risk of acute exacerbation, further underscoring the need for additional prospective studies to clarify their roles and associated risks.

### 4.3 RP-ILD

In the RP-ILD group, our study identified four major risk factors for IIM patients: short disease duration, high CRP, Ro-52 positivity, and MDA5 antibody presence. These factors are regarded as key pathogenic contributors to RP-ILD in numerous studies, aligning with our findings from the meta-analysis of multivariate adjusted data. Ro-52 (84) and MDA5 antibodies (85) are known risk factors for RP-ILD, playing important roles in immune regulation and the progression of fibrosis. Ro-52 positive patients may face a stronger immune dysregulation response, leading to more rapid lung fibrosis progression, while the presence of MDA5 antibodies is closely associated with acute and extensive lung injury in inflammatory myopathy. Our research further confirms these factors as independent risk factors for RP-ILD. Recently, a predictive model built on single-factor variables using machine learning also identified CRP levels, Ro-52, and MDA5 as the most critical risk factors (86). This underscores that high CRP levels may serve as early indicators of rapid disease progression. CRP, as a marker of systemic inflammation, reflects higher disease activity, suggesting that these patients have a more intense immune response that could lead to rapid progression (87). Therefore, early identification and proactive intervention for these high-risk patients may help improve prognosis and slow the progression of RP-ILD. Among the nine other risk factors with statistically significant differences identified in our study, although many researchers have previously explored them, we were unable to achieve highly reliable results, possibly due to the limited number of included studies. These factors demonstrated a certain degree of heterogeneity across different studies, indicating the complexity of the pathological mechanisms underlying RP-ILD. Future research should focus on larger-scale prospective cohort studies to further validate the role of these risk factors in RP-ILD, especially in different subtypes of IIM patients.

This study has several advantages: first, it is the first meta-analysis related to risk factors for SARD-ILD progression, AE, and RP-ILD, incorporating data from multivariate adjusted risk factors, which offers higher credibility compared to univariate analyses; second, our study not only conducted sensitivity analyses for risk factors based on more than five included studies but also employed Egger’s test to assess publication bias and used the trim-and-fill method to adjust results, ensuring the stability and reliability of our findings; third, we performed a meta-analysis of multiple risk factors and categorized them by credibility, providing more precise insights for future research; lastly, we compiled risk factors that could not be included in the meta-analysis, which are all multivariate adjusted data of high significance, summarized in tables to serve as a reference for future related researchers.

It is unfortunate that, our study also has limitations. First, although we considered the heterogeneity caused by different SARD types from the outset of the study and recognized the need for subgroup analyses by type, the included studies were not evenly distributed across the various SARD types, making it difficult to draw conclusions about SARD types. Second, the majority of studies included in the AE group were primarily RA-ILD, which may not apply to all SARD types and could have an impact on the accuracy of the results. Finally, the definition of progression was only established in the guidelines (7), and there are differences between this definition and those of studies published before 2022. There is still no authoritative definition for AE and RP-ILD, leading to some variability among the included studies that could affect the results. Future research should aim for unified standards and conduct large-scale, prospective cohort studies for validation.

## 5 Conclusion

In conclusion, our meta-analysis identified male sex, UIP pattern on HRCT, extensive lung involvement, and advanced age as significant risk factors for the progression of SARD-ILD. Additionally, high CRP, Ro-52 positivity, and of MDA5 antibodies were found to be significant risk factors for the development of RP-ILD in patients with IIM. We also discovered several potential risk factors that may be associated with the progression of SARD-ILD and acute exacerbations, as well as the occurrence of RP-ILD in IIM patients. This study aids researchers, clinicians, and patients in better understanding the risk factors associated with SARD-ILD exacerbation, thereby improving prognosis, enhancing quality of life, and extending survival.

## Data Availability

The original contributions presented in the study are included in the article/Supplementary material, further inquiries can be directed to the corresponding author.
