# Supplementary material for: Determinants for worsening in systemic autoimmune rheumatic disease-associated interstitial lung disease: a systematic review and meta-analysis of cohort studies
Source: Front Med (Lausanne). 2024 Nov 27;11:1465753. doi: 10.3389/fmed.2024.1465753 (PMC11631603; doi:10.3389/fmed.2024.1465753)
Supplement: Supplementary file 1 [file Data_Sheet_1.docx]

**Supplementary appendix**

[1. Search equation via PubMed, EMBASE, Scopus, Cochrane library and Web of Science databases 3](#_Toc21213)

[2. Supplementary Table 1: Search equation via PubMed, EMBASE, Scopus, Cochrane library and Web of Science databases 8](#_Toc28775)

[3. Supplementary Table 2: Quality Evaluation of the Included Studies 18](#_Toc4695)

[4. Meta-analysis results of the progression of CTD-ILD. 22](#_Toc10511)

[Male 22](#_Toc27326)

[UIP patterns on HRCT 24](#_Toc25051)

[extensive lung involvement 26](#_Toc7929)

[Age 27](#_Toc15217)

[FVC 29](#_Toc30982)

[DLCO 31](#_Toc7165)

[ANA+ 32](#_Toc8674)

[ESR 32](#_Toc32265)

[diffuse skin involvement 33](#_Toc12675)

[mRSS 33](#_Toc19191)

[KL-6 34](#_Toc32740)

[RTX 34](#_Toc18035)

[Arthritis 35](#_Toc18228)

[Dysphagia or reflux 35](#_Toc31321)

[Shortness of breath 36](#_Toc26782)

[Smoking history 36](#_Toc20193)

[Disease duration 37](#_Toc28752)

[CCP 38](#_Toc6414)

[CRP 39](#_Toc6817)

[RF 39](#_Toc31155)

[Reticulation on HRCT 40](#_Toc27530)

[Pulmonary hypertension 40](#_Toc31119)

[Congestive heart failure 41](#_Toc13010)

[CYC 41](#_Toc29512)

[IS 42](#_Toc27284)

[LEF 42](#_Toc8790)

[MTX 43](#_Toc2832)

[Steroids 43](#_Toc9748)

[5.Meta-analysis results of the acute exacerbation of CTD-ILD. 45](#_Toc1506)

[FVC 45](#_Toc662)

[UIP 45](#_Toc27670)

[Smoking history 46](#_Toc13555)

[Age 47](#_Toc7600)

[MTX 48](#_Toc24361)

[Steroids 48](#_Toc5794)

[6.Meta-analysis results of the rapidly progressive of CTD-ILD 49](#_Toc28605)

[Disease duration 49](#_Toc19526)

[CRP 50](#_Toc3017)

[Ro-52 51](#_Toc26583)

[MDA5 52](#_Toc30235)

[Age 54](#_Toc19835)

[Male 54](#_Toc17540)

[Lymphocytes 55](#_Toc7391)

[LDH 55](#_Toc31897)

[SF 56](#_Toc32632)

[CEA 57](#_Toc15210)

[Fever 57](#_Toc31190)

[Arthritis 58](#_Toc26620)

[Muscle weakness 58](#_Toc24766)

[AST 59](#_Toc11913)

[ALT 59](#_Toc22499)

[7.Supplementary Table 3: Summary of risk factors not included in the meta-analysis 60](#_Toc13013)

## **Search equation via PubMed, EMBASE, Scopus, Cochrane library and Web of Science databases**

**Search strategies for the different databases ran on January 1, 2024**

**PubMed (269)**

("Connective Tissue Diseases"[Mesh] OR "Mixed Connective Tissue Disease"[All Fields] OR "Arthritis, Rheumatoid"[All Fields] OR "Scleroderma, Systemic"[All Fields] OR "Sjogren's syndrome"[All Fields] OR "Dermatomyositis"[All Fields] OR "Undifferentiated Connective Tissue Diseases"[All Fields])

AND ("Lung Diseases, Interstitial"[Mesh])

AND (("Rapid Progress"[All Fields] OR "Acute Exacerbation"[All Fields] OR "Disease Progression"[All Fields] OR "Progression, Disease"[All Fields] OR "Clinical Course"[All Fields] OR "Clinical Progression"[All Fields] OR "Progression, Clinical"[All Fields] OR "Disease Exacerbation"[All Fields] OR "Exacerbation, Disease"[All Fields] OR "Clinical Deterioration"[All Fields] OR "Exacerbation"[All Fields] OR "Deterioration"[All Fields] OR "Course"[All Fields] OR "Progression"[All Fields] OR "Symptom Flare Up"[All Fields] OR "Flare Up, Symptom"[All Fields] OR "Symptom Flare Ups"[All Fields] OR "Symptom Flaring Up"[All Fields] OR "Acute Symptom Flare"[All Fields] OR "Symptom Flareup"[All Fields] OR "Flareups, Symptom"[All Fields] OR "Symptom Flareups"[All Fields] OR "Symptom Flare-up"[All Fields] OR "Flare-up, Symptom"[All Fields] OR "Symptom Exacerbation"[All Fields] OR "Exacerbation, Symptom"[All Fields] OR "Symptom Increase"[All Fields] OR "Increase, Symptom"[All Fields] OR "Symptom Magnification"[All Fields] OR "Magnification, Symptom"[All Fields] OR "Symptom Worsening"[All Fields] OR "Worsening, Symptom"[All Fields] OR "Symptom Exaggeration"[All Fields] OR "Exaggeration, Symptom"[All Fields])

AND ("Risk Factors"[All Fields] OR "Factor, Risk"[All Fields] OR "Factor, Social Risk"[All Fields] OR "Risk Factor, Social"[All Fields] OR "Social Risk Factor"[All Fields] OR "Health Correlates"[All Fields] OR "Correlates, Health"[All Fields] OR "Population at Risk"[All Fields] OR "Risk Score"[All Fields] OR "Score, Risk"[All Fields] OR "Risk Factor Score"[All Fields] OR "Score, Risk Factor"[All Fields])

**Embase (369)**

('connective tissue disease'/exp OR 'connective tissue defect':ti,ab,kw OR 'connective tissue diseases':ti,ab,kw OR 'connective tissue disorder':ti,ab,kw OR 'connective tissue dysplasia':ti,ab,kw OR 'mesenchymal disease':ti,ab,kw OR 'connective tissue disease':ti,ab,kw)

AND ('interstitial lung disease'/exp OR 'diffuse interstitial pneumopathy':ti,ab,kw OR 'diffuse parenchyma lung disease':ti,ab,kw OR 'diffuse parenchymal lung disease':ti,ab,kw OR 'diffuse parenchymal pulmonary disease':ti,ab,kw OR 'diffuse parenchymal pulmonary disorder':ti,ab,kw OR 'interstitial lung diseases':ti,ab,kw OR 'interstitial lung disorder':ti,ab,kw OR 'interstitial pneumopathy':ti,ab,kw OR 'interstitial pulmonary disease':ti,ab,kw OR 'interstitial pulmonary disorder':ti,ab,kw OR 'lung disease, interstitial':ti,ab,kw OR 'lung diseases, interstitial':ti,ab,kw OR 'pneumopathy, interstitial':ti,ab,kw OR 'interstitial lung disease':ti,ab,kw)

AND ('Rapid Progress':ti,ab,kw OR 'Acute Exacerbation':ti,ab,kw OR 'disease progression':ti,ab,kw OR 'progression, disease':ti,ab,kw OR 'clinical course':ti,ab,kw OR 'clinical progression':ti,ab,kw OR 'progression, clinical':ti,ab,kw OR 'disease exacerbation':ti,ab,kw OR 'exacerbation, disease':ti,ab,kw OR 'clinical deterioration':ti,ab,kw OR 'exacerbation':ti,ab,kw OR 'deterioration':ti,ab,kw OR 'course':ti,ab,kw OR 'progression':ti,ab,kw OR 'symptom flare up':ti,ab,kw OR 'flare up, symptom':ti,ab,kw OR 'symptom flare ups':ti,ab,kw OR 'symptom flaring up':ti,ab,kw OR 'acute symptom flare':ti,ab,kw OR 'symptom flareup':ti,ab,kw OR 'flareups, symptom':ti,ab,kw OR 'symptom flareups':ti,ab,kw OR 'symptom flare-up':ti,ab,kw OR 'flare-up, symptom':ti,ab,kw OR 'symptom exacerbation':ti,ab,kw OR 'exacerbation, symptom':ti,ab,kw OR 'symptom increase':ti,ab,kw OR 'increase, symptom':ti,ab,kw OR 'symptom magnification':ti,ab,kw OR 'magnification, symptom':ti,ab,kw OR 'symptom worsening':ti,ab,kw OR 'worsening, symptom':ti,ab,kw OR 'symptom exaggeration':ti,ab,kw OR 'exaggeration, symptom':ti,ab,kw)

AND ('risk factors':ti,ab,kw OR 'factor, risk':ti,ab,kw OR 'factor, social risk':ti,ab,kw OR 'risk factor, social':ti,ab,kw OR 'social risk factor':ti,ab,kw OR 'health correlates':ti,ab,kw OR 'correlates, health':ti,ab,kw OR 'population at risk':ti,ab,kw OR 'risk score':ti,ab,kw OR 'score, risk':ti,ab,kw OR 'risk factor score':ti,ab,kw OR 'score, risk factor':ti,ab,kw)

**Scopus (542)**

TITLE-ABS-KEY ( ( "Connective Tissue Diseases" OR "Disease, Connective Tissue" OR "Anetoderma" OR "Cartilage Diseases" OR "Chondromalacia Patellae" OR "Laryngomalacia" OR "Osteochondritis" OR "Pectus Carinatum" OR "Polychondritis, Relapsing" OR "Tietze&apos;s Syndrome" OR "Tracheobronchomalacia" OR "Cellulitis" OR "Orbital Cellulitis" OR "Collagen Diseases" OR "Ehlers-Danlos Syndrome" OR "Epidermolysis Bullosa Dystrophica" OR "Keloid" OR "Necrobiotic Disorders" OR "Nephritis, Hereditary" OR "Osteogenesis Imperfecta" OR "Cutis Laxa" OR "Dermatomyositis" OR "Dupuytren Contracture" OR "Fibromatosis, Plantar" OR "Homocystinuria" OR "Lipedema" OR "Lupus Erythematosus, Cutaneous" OR "Lupus Erythematosus, Discoid" OR "Lupus Erythematosus, Systemic" OR "Lupus Nephritis" OR "Lupus Vasculitis, Central Nervous System" OR "Marfan Syndrome" OR "Mixed Connective Tissue Disease" OR "Mucinoses" OR "Ganglion Cysts" OR "Mucinosis, Follicular" OR "Mucopolysaccharidoses" OR "Myofibroma" OR "Myopericytoma" OR "Noonan Syndrome" OR "Osteopoikilosis" OR "Panniculitis" OR "Erythema Induratum" OR "Panniculitis, Lupus Erythematosus" OR "Panniculitis, Nodular Nonsuppurative" OR "Panniculitis, Peritoneal" OR "Penile Induration" OR "Pseudoxanthoma Elasticum" OR "Rheumatic Diseases" OR "Arthritis, Juvenile" OR "Arthritis, Rheumatoid" OR "Hyperostosis, Sternocostoclavicular" OR "Polymyalgia Rheumatica" OR "Rheumatic Fever" OR "Scleroderma, Localized" OR "Scleroderma, Systemic" OR "Scleroderma, Diffuse" OR "Scleroderma, Limited" OR "Undifferentiated Connective Tissue Diseases" OR "Weill-Marchesani Syndrome" OR "Sjogren's syndrome" )

AND ( "Lung Diseases, Interstitial" OR "Alveolitis, Extrinsic Allergic" OR "Bird Fancier&apos;s Lung" OR "Farmer&apos;s Lung" OR "Silo Filler&apos;s Disease" OR "Trichosporonosis" OR "Anti-Glomerular Basement Membrane Disease" OR "Granulomatosis with Polyangiitis" OR "Histiocytosis, Langerhans-Cell" OR "Eosinophilic Granuloma" OR "Pneumoconiosis" OR "Anthracosis" OR "Asbestosis" OR "Berylliosis" OR "Byssinosis" OR "Caplan Syndrome" OR "Siderosis" OR "Silicosis" OR "Pulmonary Fibrosis" OR "Idiopathic Pulmonary Fibrosis" OR "Radiation Pneumonitis" OR "Sarcoidosis, Pulmonary" OR "Diffuse Parenchymal Lung Disease" OR "Interstitial Lung Disease" OR "Lung Disease, Interstitial" OR "Pneumonia, Interstitial" OR "Interstitial Pneumonia" OR "Pneumonias, Interstitial" OR "Interstitial Pneumonitis" OR "Pneumonitides, Interstitial" )

AND ( "Rapid Progress" OR "Acute Exacerbation" OR "Disease Progression" OR "Progression, Disease" OR "Clinical Course" OR "Clinical Progression" OR "Progression, Clinical" OR "Disease Exacerbation" OR "Exacerbation, Disease" OR "Clinical Deterioration" OR "Exacerbation" OR "Deterioration" OR "Course" OR "Progression" OR "Symptom Flare Up" OR "Flare Up, Symptom" OR "Symptom Flare Ups" OR "Symptom Flaring Up" OR "Acute Symptom Flare" OR "Symptom Flareup" OR "Flareups, Symptom" OR "Symptom Flareups" OR "Symptom Flare-up" OR "Flare-up, Symptom" OR "Symptom Exacerbation" OR "Exacerbation, Symptom" OR "Symptom Increase" OR "Increase, Symptom" OR "Symptom Magnification" OR "Magnification, Symptom" OR "Symptom Worsening" OR "Worsening, Symptom" OR "Symptom Exaggeration" OR "Exaggeration, Symptom" )

AND ( "Risk Factors" OR "Factor, Risk" OR "Factor, Social Risk" OR "Risk Factor, Social" OR "Social Risk Factor" OR "Health Correlates" OR "Correlates, Health" OR "Population at Risk" OR "Risk Score" OR "Score, Risk" OR "Risk Factor Score" OR "Score, Risk Factor" ) )

**Web of Science (348)**

TS=("Connective Tissue Diseases" OR "Disease, Connective Tissue" OR "anetodermia" OR "Cartilage Diseases" OR "Chondromalacia Patellae" OR "Laryngomalacia" OR "Osteochondritis" OR "Pectus Carinatum" OR "Polychondritis, Relapsing" OR "Tietze's Syndrome" OR "Tracheobronchomalacia" OR "Cellulitis" OR "Orbital Cellulitis" OR "Collagen Diseases" OR "Ehlers-Danlos Syndrome" OR "Epidermolysis Bullosa Dystrophica" OR "Keloid" OR "Necrobiotic Disorders" OR "Nephritis, Hereditary" OR "Osteogenesis Imperfecta" OR "Cutis Laxa" OR "Dermatomyositis" OR "Dupuytren Contracture" OR "Fibromatosis, Plantar" OR "Homocystinuria" OR "Lipedema" OR "Lupus Erythematosus, Cutaneous" OR "Lupus Erythematosus, Discoid" OR "Lupus Erythematosus, Systemic" OR "Lupus Nephritis" OR "Lupus Vasculitis, Central Nervous System" OR "Marfan Syndrome" OR "Mixed Connective Tissue Disease" OR "mucinosis" OR "Ganglion Cysts" OR "Mucinosis, Follicular" OR "Mucopolysaccharidoses" OR "Myxedema" OR "Scleredema Adultorum" OR "Scleromyxedema" OR "Neoplasms, Connective Tissue" OR "myelofibroma" OR "Myopericytoma" OR "Noonan Syndrome" OR "Osteopoikilosis" OR "Panniculitis" OR "Erythema Induratum" OR "Panniculitis, Lupus Erythematosus" OR "Panniculitis, Nodular Nonsuppurative" OR "Panniculitis, Peritoneal" OR "Penile Induration" OR "Pseudoxanthoma Elasticum" OR "Rheumatic Diseases" OR "Arthritis, Juvenile" OR "Arthritis, Rheumatoid" OR "Hyperostosis, Sternocostoclavicular" OR "Polymyalgia Rheumatica" OR "Rheumatic Fever" OR "Scleroderma, Localized" OR "Scleroderma, Systemic" OR "Scleroderma, Diffuse" OR "Scleroderma, Limited" OR "Undifferentiated Connective Tissue Diseases" OR "Weill-Marchesani Syndrome" OR "Sjogren's syndrome")

AND TS=("Lung Diseases, Interstitial" OR "Alveolitis, Extrinsic Allergic" OR "Bird Fancier's Lung" OR "Farmer's Lung" OR "Silo Filler's Disease" OR "Trichosporonosis" OR "Anti-Glomerular Basement Membrane Disease" OR "Granulomatosis with Polyangiitis" OR "Histiocytosis, Langerhans-Cell" OR "Eosinophilic Granuloma" OR "Pneumoconiosis" OR "Anthracosis" OR "Asbestosis" OR "Berylliosis" OR "Byssinosis" OR "Caplan Syndrome" OR "Siderosis" OR "Silicosis" OR "Pulmonary Fibrosis" OR "Idiopathic Pulmonary Fibrosis" OR "Radiation Pneumonitis" OR "Sarcoidosis, Pulmonary" OR "Diffuse Parenchymal Lung Disease" OR "Interstitial Lung Disease" OR "Lung Disease, Interstitial" OR "Pneumonia, Interstitial" OR "Interstitial Pneumonia" OR "Pneumonias, Interstitial" OR "Interstitial Pneumonitis" OR "Pneumonitides, Interstitial")

AND TS=("Rapid Progress" OR "Acute Exacerbation" OR "Disease Progression" OR "Progression, Disease" OR "Clinical Course" OR "Clinical Progression" OR "Progression, Clinical" OR "Disease Exacerbation" OR "Exacerbation, Disease" OR "Clinical Deterioration" OR "Exacerbation" OR "Deterioration" OR "Course" OR "Progression" OR "Symptom Flare Up" OR "Flare Up, Symptom" OR "Symptom Flare Ups" OR "Symptom Flaring Up" OR "Acute Symptom Flare" OR "Symptom Flareup" OR "Flareups, Symptom" OR "Symptom Flareups" OR "Symptom Flare-up" OR "Flare-up, Symptom" OR "Symptom Exacerbation" OR "Exacerbation, Symptom" OR "Symptom Increase" OR "Increase, Symptom" OR "Symptom Magnification" OR "Magnification, Symptom" OR "Symptom Worsening" OR "Worsening, Symptom" OR "Symptom Exaggeration" OR "Exaggeration, Symptom")

AND TS=("Risk Factors" OR "Factor, Risk" OR "Factor, Social Risk" OR "Risk Factor, Social" OR "Social Risk Factor" OR "Health Correlates" OR "Correlates, Health" OR "Population at Risk" OR "Risk Score" OR "Score, Risk" OR "Risk Factor Score" OR "Score, Risk Factor")

**Cochrance (15)**

("Risk Factors" OR "Factor, Risk" OR "Factor, Social Risk" OR "Risk Factor, Social" OR "Social Risk Factor" OR "Health Correlates" OR "Correlates, Health" OR "Population at Risk" OR "Risk Score" OR "Score, Risk" OR "Risk Factor Score" OR "Score, Risk Factor") AND

("Disease Progression" OR "Progression, Disease" OR "Clinical Course" OR "Clinical Progression" OR "Progression, Clinical" OR "Disease Exacerbation" OR "Exacerbation, Disease" OR "Clinical Deterioration" OR "Exacerbation" OR "Deterioration" OR "Course" OR "Progression" OR "Symptom Flare Up" OR "Flare Up, Symptom" OR "Symptom Flare Ups" OR "Symptom Flaring Up" OR "Acute Symptom Flare" OR "Symptom Flareup" OR "Flareups, Symptom" OR "Symptom Flareups" OR "Symptom Flare-up" OR "Flare-up, Symptom" OR "Symptom Exacerbation" OR "Exacerbation, Symptom" OR "Symptom Increase" OR "Increase, Symptom" OR "Symptom Magnification" OR "Magnification, Symptom" OR "Symptom Worsening" OR "Worsening, Symptom" OR "Symptom Exaggeration" OR "Exaggeration, Symptom") AND

("Lung Diseases, Interstitial" OR "Alveolitis, Extrinsic Allergic" OR "Bird Fancier's Lung" OR "Farmer's Lung" OR "Silo Filler's Disease" OR "Trichosporonosis" OR "Anti-Glomerular Basement Membrane Disease" OR "Granulomatosis with Polyangiitis" OR "Histiocytosis, Langerhans-Cell" OR "Eosinophilic Granuloma" OR "Pneumoconiosis" OR "Anthracosis" OR "Asbestosis" OR "Berylliosis" OR "Byssinosis" OR "Caplan Syndrome" OR "Siderosis" OR "Silicosis" OR "Pulmonary Fibrosis" OR "Idiopathic Pulmonary Fibrosis" OR "Radiation Pneumonitis" OR "Sarcoidosis, Pulmonary" OR "Diffuse Parenchymal Lung Disease" OR "Interstitial Lung Disease" OR "Lung Disease, Interstitial" OR "Pneumonia, Interstitial" OR "Interstitial Pneumonia" OR "Pneumonias, Interstitial" OR "Interstitial Pneumonitis" OR "Pneumonitides, Interstitial") AND

("Connective Tissue Diseases" OR "Disease, Connective Tissue" OR "Anetoderma" OR "Cartilage Diseases" OR "Chondromalacia Patellae" OR "Laryngomalacia" OR "Osteochondritis" OR "Pectus Carinatum" OR "Polychondritis, Relapsing" OR "Tietze's Syndrome" OR "Tracheobronchomalacia" OR "Cellulitis" OR "Orbital Cellulitis" OR "Collagen Diseases" OR "Ehlers-Danlos Syndrome" OR "Epidermolysis Bullosa Dystrophica" OR "Keloid" OR "Necrobiotic Disorders" OR "Nephritis, Hereditary" OR "Osteogenesis Imperfecta" OR "Cutis Laxa" OR "Dermatomyositis" OR "Dupuytren Contracture" OR "Fibromatosis, Plantar" OR "Homocystinuria" OR "Lipedema" OR "Lupus Erythematosus, Cutaneous" OR "Lupus Erythematosus, Discoid" OR "Lupus Erythematosus, Systemic" OR "Lupus Nephritis" OR "Lupus Vasculitis, Central Nervous System" OR "Marfan Syndrome" OR "Mixed Connective Tissue Disease" OR "Mucinoses" OR "Ganglion Cysts" OR "Mucinosis, Follicular" OR "Mucopolysaccharidoses" OR "Myofibroma" OR "Myopericytoma" OR "Noonan Syndrome" OR "Osteopoikilosis" OR "Panniculitis" OR "Erythema Induratum" OR "Panniculitis, Lupus Erythematosus" OR "Panniculitis, Nodular Nonsuppurative" OR "Panniculitis, Peritoneal" OR "Penile Induration" OR "Pseudoxanthoma Elasticum" OR "Rheumatic Diseases" OR "Arthritis, Juvenile" OR "Arthritis, Rheumatoid" OR "Hyperostosis, Sternocostoclavicular" OR "Polymyalgia Rheumatica" OR "Rheumatic Fever" OR "Scleroderma, Localized" OR "Scleroderma, Systemic" OR "Scleroderma, Diffuse" OR "Scleroderma, Limited" OR "Undifferentiated Connective Tissue Diseases" OR "Weill-Marchesani Syndrome" OR "Sjogren's syndrome"**)**

## **Supplementary Table 1: Search equation via PubMed, EMBASE, Scopus, Cochrane library and Web of Science databases**

| **First author** | **Year** | **Region** | **Study design** | **Observation period** | **Sample size** | **CTD** | **Type** | **Criteria for progression of interstitial lung disease** | **Adjusted** | **Data** |
| --- | --- | --- | --- | --- | --- | --- | --- | --- | --- | --- |
| I. MARIE | 2013 | France | Retrospective | 1996-2010 | 66 | IIM-ASS | Progression | Changes of ≥ 10% in FVC and/or ≥ 15% in DLCO were considered to be significant, and were used as determinants of deterioration | Age, UIP, Ventilatory insufficiency due to striated muscle weakness | OR |
| Giacomo De Luca | 2015 | Italy | Retrospective | NR | 80 | SSc | Progression | A modification of FVC >10% and DLCO >15% at follow-up has been considered a clinically significant variation an increase of at least 2 points in the interstitial and/or alveolar score was considered a significant worsening of lung impairment. | The diffuse skin involvement, long disease duration | RR |
| Young Seok Lee | 2016 | Korea | Retrospective | 2005-2012 | 62 | RA | Progression | Improvement or progression was defined as a 10% or greater change in the FVC and/or a 15% or greater decrease in the DLCO, with or without aggravation of dyspnea and the HRCT findings | FVC, KL-6 | OR |
| Tomoyuki Fujisawa | 2016 | Japan | Retrospective | 1990-2013 | 34 | IIM | Progression | (1) symptomatic exacerbation(e.g., dyspnea upon exertion); (2) an increase in opacity on a chest HRCT scan; and (3) a 10% decrease in percentage of predicted FVC or ＞10 mmHg decrease in PaO2 | Positeve anti-PL-7 antibody, FVC | OR |
| Sébastien Rivière | 2018 | France | Prospective | 2005-2012 | 106 | SSC | Progression | Defined as a 10% decrease in TLC or FVC from baseline, or death | CC16> 33 ng/ml, Serum creatinine, Age, Smoking history, Duration of SSc, mRSS, FVC, DLCO, IS | HR |
| Silje Reiseter | 2018 | Norway | Prospective | 2005-2008 | 49 | MCTD | Progression | ILD progression was defined as a disease extension as a percentage of total lung volume | Arthritis, Age, Male, Anti-Ro-52 positivity, Anti-RNP value, ILD of TLV | HR |
| Anna-Maria Hoffmann-Vold | 2018 | USA、Norway | Prospective | NR-2017 | 292 | SSc | Progression | FVC decline＞5%, or DLCO decline＞7.5%, or death within 12 months | mRss, Male sex, CX3CL1, Age at onset | OR |
| Tatiana Cobo-Ibáñez | 2019 | Spain | Retrospective | 1980-2014 | 112 | IIM | Progression | A decrease of FVC ≥ 10%, or between 5 and 10% with a  decrease of DLCO ≥ 15% | Smoking history, Ana+, CRP, Arthritis | HR |
| Hui Liu | 2019 | China | Retrospective study | 2009-2018 | 69 | ASS | Progression | Deterioration and regression of the overall disease extent were respectively defined by an increase or decrease of at least 10% of the overall disease extent | Fever at presentation  CD3+CD4+ cell counts | OR |
| Qiang Fu | 2019 | China | Retrospective | 2008-2014 | 184 | RA | Progression | A decrease of FVC > 10% or DLCO >15% predicted, worsening of ILDS or death from respiratory failure due to ILD and/or pneumonia | Age >60 years, Smoking history  Anti-CCP antibody high titer positive, UIP, CYC, MTX, FVC% <70%, DLCO %< 45%, ILD occurs within 10 years of RA diagnosed | OR |
| Takeshi Mochizuki | 2019 | Japan | Retrospective | NR | 131 | RA | Progression | The HRCT findings were classified as follows: grade 0, ILD not determined; grade 1, ILD extended by less than one-third; grade 2, extended by more than one-third but less than two-thirds; and grade 3, extended by more than two-thirds.We defined “deterioration” as occurring when the grade of the HRCT findings increased | MTX, KL-6 | OR |
| Muriel Elhai | 2019 | France, Norway | Prospective | NR | 234 | SSc | Progression | DLCO decline >15% and FVC decline >10% were defined as clinically significant | Male, Immunosuppressive, High CCL18 | OR |
| Luling Li | 2019 | China | Retrospective | 2008-2017 | 213 | RA | Progression | Progressive fibrosis was defined as a new development or an increase in the extent of reticular pattern and/or honeycombing on follow-up CT | Age >65 years, Male, Smoking history, RA duration at baseline months, Shortness of breath, Anti-CCP antibody high titer positive, Overall extent of lung abnormalities | OR |
| Luling Li | 2020 | China | Retrospective | 2008-2017 | 185 | RA | Progression | ILD progression was defined as meeting any of the following: a decrease of FVC >10% or DLCO > 15% predicted and worsening of ILD score or death from respiratory failure due to ILD and/or pneumonia | Age >60 years  Anti-CCP antibody high titer positive, DLCO%<45%, Possible UIP, MTX, Patients with respiratory symptoms | OR |
| Cristina Vadillo | 2020 | Spain | Prospective | 2007-2018 | 68 | RA | Progression | Defined as a decline of 5% in the predicted FVC per visit compared with the previous one. Pulmonary function tests were performed at baseline and every 6–12 months | Gender (female vs male), Age at ILD diagnosis, Anti-CCP (>300 vs lower), ESR (>40 vs lower), Dysphagia or reflux, FVC, UIP, Glucocorticoids, RTX, Emphysema, Lag time to ILD diagnosis | HR |
| Yuetong Xu | 2021 | China | Retrospective | 2011-2017 | 113 | PSS | Progression | ILD progression was defined as a decrease of FVC > 10% predicted or DLCO > 15% predicted,13 an elevation of the ILD score on HRCT, or death from respiratory failure due to ILD and/or pneumonia | CYC, Extensive lung involvement, UIP, Decreased C3(%), Raynaud's syndrome, Hypoproteinemia | OR |
| Yu-Hsiang Chiu | 2021 | Netherlands | Retrospective | 2004-2018 | 150 | CTD | Progression | A significant and clinically relevant change in PFT was defined as ≥ 10% change in FVC or ≥15% change in DLCO within 2 years | Congestive heart failure, Pulmonary hypertension, Diabetes mellitus, Obesity, RTX, steroid-use, Anti-CCP, Anti-Ro, Anti-dsDNA, Anti-Scl-PM, Coronary artery disease | OR |
| Anna-Maria Hoffmann-Vold | 2021 | Europe | Retrospective | NR | 826 | SSc | Progression | A decline in FVC of ≥10%, or a decline in FVC of 5% to 10% along with a decline in DLCO of 15%, is a proposed definition of progressive fibrosis | FVC, shorter disease duration, higher erythrocyte sedimentation rate, Dysphagia or reflux, mRSS | OR |
| Natalia Mena-Vázquez | 2021 | Spain | Retrospective | 2015-2020 | 204 | CTD | Progression | progression (worsening of FVC > 10%  or DLCO > 15% and radiological progression); or death | Smoking history, UIP, FVC <80%, RA VS SSC, IM | HR |
| Satoshi Watanabe | 2022 | Japan | Retrospective | 2004-2021 | 77 | SSC | Progression | A relative decrease in FVC from baseline of ≥10%, or a relative decrease in FVC of 5%─10% combined with a relative decrease in DLCO of ≥15% | Diffuse cutaneous SSc, Anti-topoisomerase-I positive, Extensive lung involvement, KL-6 > 1273 U/ml | HR |
| Ning Chen | 2022 | China | Retrospective | 2010-2021 | 75 | RA | Progression | HRCT scores increased, notably, the new bilateral ground-glass opacity and/or consolidation could not be fully explained by cardiac failure or fluid overload, or a decline of 5% in the predicted FVC compared with the baseline FVC | Gender, Smoking, RF-IgA>200, UIP, LEF, HRCT scores at baseline | HR |
| Lei Liu | 2022 | China | Retrospective | 2014-2021 | 201 | RA | Progression | decrease in FVC of 15% of the predicted value or a decline in FVC of 10% combined with a decline in DLCO of 15% from the baseline PFTs | Male, Smoking history, Definite UIP, Reticulation, Fibrosis score, CYC, Interlobular septal thickening, HAQ-DI, GGO, High DAS28-ESR | OR |
| Na Zhao | 2022 | China | Retrospective | 2013-2020 | 111 | ASS | Progression | Deterioration and regression of the overall ILD range in HRCT images were defined as an increase or decrease of at least 10% of the over all ILD image | Initial glucocorticoid dose, CD3-CD19+ | OR |
| Shi‑Hao He | 2022 | China | Retrospective | 2015-2019 | 83 | PSS | Progression | (1) a relative decline in FVC from baseline of ≥ 10% or in DLCO of ≥ 15%; (2) a relative decline in FVC of 5–9% plus increased extent of fbrosis on HRCT; (3) a relative decline in FVC of 5–9% plus worsening of respiratory symptoms; (4) worsened respiratory symptoms plus increased extent of fbrosis on HRCT | Male, LDH, FVC, Reticular , Sicca onset | OR |
| Ju Kwang Lee | 2023 | Korea | Retrospective | 2007-2022 | 197 | CTD | Progression | PPF as meeting a minimum of two of the following three criteria: a decrease of at least 5% in the absolute value of the FVC or a decrease of at least 10% in DLCO, worsening of symptoms, and radiological evidence indicating disease progression | Baseline KL-6≥1000, Baseline pulmonary function test FVC, DLCO, Baseline Albumin | HR |
| Dandan Chai | 2023 | China | Retrospective | 2017-2021 | 98 | RA | Progression | Progression was defined as the appearance of new imaging features and/or an increase in the extent or density of abnormal imaging compared to the initial scan | CRP>0.8 mg/dl, ESR>15 mm/h, Extensive lung involvement | OR |
| Gonçalo Boleto | 2023 | multicenter | Retrospective | NR | 150 | MCTD | Progression | Absolute FVC decline of ≥10% between baseline and last available visit was used to define major ILD progression | Digital ulcers | HR |
| Kyung‑Ann Lee | 2023 | Korea | Retrospective | 2013-2021 | 39 | PSS | Progression | The progression of lung disease was defined as the worsening of FVC > 10% or DLCO > 15% | Follow-up duration, UIP | OR |
| Kyuhwan Kim | 2023 | Korea | Retrospective | 2019-2022 | 110 | CTD | Progression | Decline rate of FVC⩾10% or DLCO⩾15% during follow-up | UIP, ANA positivity, RF positivity, Cardiac disease (HTN, IHD, CHF), Systemic steroid, Immunomodulating agents, Dysphagia or reflux, Time interval between CTD and ILD diagnosis, ANCA, Antifibrotics | OR |
| Yu-Hsuan Chen | 2023 | China | Retrospective | 2015-2021 | 68 | PSS | Progression | PF-ILD was defined as a relative decline in FVC≧10% of predicted values, a relative decline in FVC≧5–10% of predicted values with worsening of respiratory symptoms or increased fibrosis extent on HRCT, or worsening of symptoms and imaging in previous 24 months | Albumin level at 3rd month, IgG level change during first 3 months of ILD diagnosis | OR |
| Wen Zeng | 2023 | China | Retrospective | 2012-2019 | 169 | SSC | Progression | SSc-ILD patients were considered to have deteriorative SSc-ILD if the HRCT score changes less than 4% | Age, Duration of disease, MRSS, Exertional dyspnea, pulmonary hypertension, ESR, CRP, Cough  Chest tightness and pain | OR |
| Ji-Won Kim | 2023 | Korea | Multicentre prospective study | 2015-2021 | 143 | RA | Progression | (1) a decrease of ≥10% in FVC, (2) a decrease of ≥15% in DLCO, or (3) death from respiratory failure due to ILD and/or pneumonia | Age, Male, Ever-smoker, RA duration, ILD duration, RF-positive, CCP-positive, FVC, DLco, UIP, Extensive lung involvement, Glucocorticoids use, MTX use, ,LEF use, TAC, Biological or targeted synthetic DMARD, DAS28-ESR | HR |
| Y. Xu | 2016 | China | Retrospective | NR | 40 | CADM | RP-ILD | The RP-ILD was definedas a progressive deterioration of ILD within 3 months | CRP, Lymphocytes, Serum ferritin, Anti-MDA5 antibody, Skin ulcerations | OR |
| Kohei Karino | 2020 | Japan | Retrospective | 2014-2019 | 41 | DM、CADM | RP-ILD | RPILD was defined as a progressive deterioration of ILD with respect to dyspnoea and radiological findings, including chest radio graph and chest high-resolution CT within 3 months from the onset of respiratory symptoms | Anti-MDA5 antibody+, CRP, KL-6, Age, Myofascia | OR |
| Qihua Yang | 2021 | China | Retrospective | 2018-2019 | 90 | anti-  MDA5 positive IIM | RP-ILD | RP-ILD was defined as following: deteriorating dyspnea on exertion, decrease in PaO2 levels by>10 mmHg within 4 weeks, or expanding GGO on HRCT within 4 weeks | Disease duration<2 months, Serum ferritin≥1500 ng/ml, KL-6≥1600 U/ml, CRP≥13 mg/L, Total CT GGO score≥4 | OR |
| Dingxian Zhu | 2021 | China | Retrospective | 2010-2019 | 41 | CADM | RP-ILD | RP-ILD was defined as a previous or concurrent diagnosis of ILD and acute and progressive worsening of dyspnoea secondary to ILD requiring hospitalization, supplementary oxygen or respiratory failure requiring intubation within 3 months of the diagnosis of ILD | Age, Ferritin, CEA | OR |
| Yu Zuo | 2022 | China | Retrospective | 2013-2019 | 175 | anti-  MDA5 positive IIM | RP-ILD | RP-ILD was defined as displaying two or more of the following within 3 months:(1) dyspnea exacerbation; (2) an increase in parenchymal abnormality on HRCT scan; and (3) one of the following physiological changes: >10% decrease in VC or >1.33 kPa decrease in arterial oxygen tension (PaO_2_) | Arthralgia, Fever, Elevated ALT, Elevated LDH, Lymphopenia, Elevated CEA, Elevated Fet, Decreased CD3+T, Decreased CD3+CD4+T, Decreased CD3+CD8+T, Elevated B2, Elevated CA153, | OR |
| Yu Zuo | 2022 | China | Retrospective | 2013-2019 | 158 | anti-  ARS antibody | RP-ILD | RP-ILD was defined as displaying two or more of the following within 3 months:(1) dyspnea exacerbation; (2) an increase in parenchymal abnormality on HRCT scan; and (3) one of the following physiological changes: >10% decrease in VC or >1.33 kPa decrease in arterial oxygen tension (PaO2) | Muscle weakness, Arthralgia, Elevated CEA, Myalgia, Elevated CA125, Elevated NSE | OR |
| Y. Zhu | 2022 | China | Retrospective | 2019-2021 | 41 | MDA5 DM | RP-ILD | The RP-ILD was defined as the presence of any the following four conditions within one month: (1) dyspnoea or cough symptoms become progressively worse and quality of life is significantly reduced; (2) decreased lung function including FVC decreased by more than 10%, or DLCO fell over 15% with the decreased FVC; (3) HRCT of chest demonstrated that the extent of interstitial pneumonia continues to increase; (4) arterial blood gas analysis suggested respiratory failure or the oxygen partial pressure reduced than 10 mmHg, independently determined by senior physicians in the rheumatology department and the respiratory department | Age >50 years, ESR, > 21 mm/H, KL-6, > 500.9 pg/mL, V sign, Shawl sign | OR |
| Hanxiao You | 2023 | China | Multicentre retrospective | NR | 272 | MDA5 DM | RP-ILD | RP-ILD was defined as the acute and progressive worsening of dyspnoea onset within 1 month, with the presence of any of the following four conditions: (1) acute and progressive worsening of dyspnoea requiring hospitalization or sup plementary oxygen; (2) lung function, including FVC, decreases by >10%, or diffusion capacity for carbon monoxide of the lung falls over 15% with the decreased FVC; (3) HRCT of the chest demon strates that the extent of interstitial abnormalities has increased >20%; (4) arterial blood gas analysis suggests respiratory failure or the oxygen partial pressure reduction is >10 mmHg. | Disease duration <3 months, CRP >8 mg/l, Anti-Ro52 positive, Anti-MDA5 | HR |
| Li Guo | 2023 | China | Retrospective | 2018-2021 | 254 | MDA5 DM | RP-ILD | RP-ILD is defined as either increased level of dyspnea and progression of initial change >10% on CT within 1 month, or deterioration to partial pressure of arterial oxygen (PaO_2_) level<60 mmHg within 3 months of the diagnosis of ILD | Serum ferrtin ≥ 823 (ng/ml), Prednisolone ≥ 67.5 (mg/d), WDFY4 rs7919656, FVC < 50%, DLCO< 30% | OR |
| Haoru Zhang | 2023 | China | Retrospective | 2018-2022 | 71 | DM | RP-ILD | RP-ILD was defined as progressive dyspnea, hypoxemia, and worsening of the radiological interstitial change at 1 month or deterioration to respiratory failure at 3 months after the onset of respiratory symptoms and no evidence of infections | Fever, Anti-MDA5 antibody positive, CRP, RF-IgA | OR |
| Zou Ruyi | 2023 | China | Retrospective study | 2017.01-2021.12 | 105 | MDA5 DM | RP-ILD | RP-ILD was defined as the presence of one of the following symptoms within 3 months of the onset of respiratory symptoms: (1) acute and progressive worsening of dyspnea requiring hospitalization or supplemental oxygen; (2) Impaired lung function, including FVC decreased by >10% or DLCO decreased by >15%; (3) The degree of interstitial abnormalities increased by more than 20% on chest HRCT; (4) Arterial blood gas analysis suggested respiratory failure or oxygen partial pressure reduction >10 mmHg | Age >53 years, sex, fever, Arthritis, Lymphocyte Counts≤740cell/μl, LDH>307 U/L, oxygenation index, Heliotrope rash | OR |
| Lu Cheng | 2024 | China | Multicenter Retrospective | 2014-2022 | 251 | MDA5+ DM | RP-ILD | RP-ILD was defined as the acute and progressive worsening of dyspnea within one month, with any of the four conditions listed below: (1) HRCT of the chest shows that the interstitial abnormalities have grown by more than 20%; (2) FVC drops by more than 10% or DLCO drops by more than 15% along with the decreased FVC, affecting lung function; (3) dyspnea that is severe and worsens over time, necessitating hospitalization or extra oxygen; and (4) arterial blood gas analysis suggests respiratory failure or the oxygen partial pressure reduction is greater than 10 mmHg | Disease duration, CRP, Anti-Ro52 positive | HR |
| HAN Yuan-yuan | 2023 | China | Retrospective | 2018-2021 | 145 | MDA5+ DM | RP-ILD | The diagnostic criteria for RP-ILD were progressive worsening of dyspnea within 3 months from the onset of pulmonary symptoms, with significant worsening of hypoxemia and radiological interstitial lung disease | LDH (≥370 IU/L), CRP (≥5 mg/L), CEA (≥5 ng/mL), Myasthenia, Neutrophil/lymphocyte ratio (≥4) | OR |
| Wang Yafei | 2023 | China | Retrospective study | 2016-2022 | 63 | MDA5+ DM | RP-ILD | The diagnostic criteria for RP-ILD were progressive worsening of dyspnea within 3 months from the onset of pulmonary symptoms, with significant worsening of hypoxemia and radiological interstitial lung disease | Lymphocyte, Ferritin, Ro52, SpO_2_<90% | OR |
| Chengyin Lv | 2023 | China | Multicenter Retrospective | 2019-2021 | 246 | MDA5+ DM | RP-ILD | RP-ILD was defined as the presence of progressive dyspnea and progressive hypoxemia as well as worsening of interstitial change as determined by chest CT within 1 month from the onset of respiratory symptoms | Disease duration ≤ 3 mouth, CRP ≥ 8 mg/L | HR |
| Lei Wang | 2024 | China | Multicenter Retrospective | 2019-2021 | 170 | MDA5+ DM | RP-ILD | (1) acute and progressive worsening of dyspne are quiring hospitalization or supplementary oxygen; (2) lung function including FVC decreases by morethan 10%, or DLCO falls over 15% with the decreased FVC; (3) HRCT of the chest demonstrates that the extent of interstitial abnormalities increased more than 20%; (4) arterial blood gas analysis suggests respiratory failure or the oxygen partial pressure reduction is more significant than 10mmHg | Sex, Short disease duration (less than 3 months), AST abnormal, LDH abnormal, CRP abnormal, SF abnormal, Anti-Ro52 antibody positive, Anti-MDA5 antibody high titer (+++) | HR |
| Takafumi Suda | 2009 | Japan | Retrospective | 1987-2007 | 83 | CTD | AE | (1) previous diagnosis of CTD-IP;(2) unexplained worsening or development of dyspnea within 30 days; (3) HRCT with new bilateral ground-glass abnormality and/or consolidation superimposed on a background reticular or honeycomb pattern; (4) no evidence of pulmonary infection by negative respiratory culture, including endotracheal aspirate or bronchoalveolar lavage, and serological test results for respiratory pathogens; and (5) exclusion of alternative causes, such as left heart failure, pulmonary embolism, and an identifiable cause of acute lung injury | Age, RA | HR |
| Fumiko Tomiyama | 2016 | Japan | Retrospective | 1990-2015 | 139 | SSc | AE | The definition of acute exacerbation or AE-ILD included a prior diagnosis of ILD, worsening of dyspnea within 1 month, new ground-glass opacities on HRCT, hypoxemia, and the exclusion of other causes for the worsening symptoms | Anticentromere, Overlap with polymyositis/dermatomyositis positive | HR |
| Mengshu Cao | 2019 | China | Retrospective | 2010-2016 | 70 | CTD | AE | AE-CTD-fILD were defined as an acute, clinically significant respiratory deterioration characterized by evidence of new widespread alveolar abnormality with: (1) Previous or concurrent diagnosis of IPF or a characterized CTD-fILD; (2) Acute worsening or development of dyspnea typically 1 month duration; (3) CT with new bilateral ground-glass opacity and/or consolidation superimposed on a background pattern consistent with UIP or possible UIP pattern; (4) Deterioration not fully explained by cardiac failure or fluid overload | Smoking history  Prior corticosteroids use  FVC, TLC, Corticosteroids reduction or discontinuation  Pulmonary hypertension | HR |
| Shinji Izuka | 2021 | Japan | Retrospective | 2007-2019 | 165 | RA | AE | AE-ILD status required a patient to meet all five of the following criteria: a previous diagnosis of RA-ILD, unexplained worsening or onset of dyspnoea within the last 30 days, new bilateral ground-glass abnormalities or consolidation superimposed on a reticular or honeycomb pattern on HRCT, no evidence of pulmonary infection and a negative respiratory culture and exclusion of alternative causes, such as left heart failure, pulmonary embolism or identifiable lung injury | Age >65 years, UIP, MTX | OR |
| Na Wang | 2020 | China | Retrospective | 2016-2019 | 45 | RA | AE | Acute exacerbations were defined according to five criteria: previously diagnosed RA-ILD; idiopathic worsening of dyspnea with in one month of onset of RA-ILD; new bilateral ground-glass lung opacities or consolidation with reticular (honeycomb) fibrosis on HRCT, no evidence of lung infection or a negative sputum culture test; and exclusion of known causes of respiratory dysfunction, such as left heart failure, pulmonary embolism, or lung injury | Age >50years , RA duration, Ever smoker, CCP＞500 titer, UIP, DLCO, MUC5B, Age at onset of RA, ACPA titer>500, Male , RA duration | HR |
| Nozomi Tanaka | 2021 | Japan | Retrospective | 2010-2019 | 125 | RA | AE | AE was defined as new HRCT findings associated with ILD (new bilateral ground-glass opacifications and/or consolidations)and any of the following: unexplained worsening or development of respiratory symptoms within 30 days of onset,SpO_2_ 90% at rest, SpO_2_≥90% during a 6-min walking test, newly added or increased corticosteroids for ILD, and exclusion of alternative causes for respiratory failure, such as pulmonary embolism and left heart failure | Age at RA-ILD diagnosis, UIP, KL-6 before AE or the last visit KL-6 levels at RA-ILD diagnosis | HR |
| Byoung Soo Kwon | 2022 | Korea | Retrospective | 1997-2019 | 310 | RA | AE | AE was defined using the criteria suggested by Collard et al., as acute worsening of dyspnea typically within 30 days, with new bilateral lung infiltration that is not fully explained by heart failure or fluid overload and no identified extra-parenchymal causes (pneumo thorax, pleural effusion, pulmonary embolism) | Ever-smoker, FVC, Steroid ± IM, 6MWT Distance, Lowest SpO2 | OR |
| Junji Otsuka | 2022 | Japan | Retrospective | 2011-2019 | 149 | RA | AE | (1) progressively worsening dyspnea; (2) new grand-glass opacities evident in HRCT scans superimposed over background reticular opacity, traction bronchiectasis, traction bronchio loectasis, or honeycombing; and (3) a reduction in resting partial pressure of oxygen in arterial blood (PaO_2_) of more than 10 mmHg compared with previous measurements. | MTX, FVC, DLCO, ILD diagnosis preceding RA onset, Alb, VC, honeycombing | OR |

**Abbreviation:** AE: Acute Exacerbation; CTD: Connective Tissue Disease; IIM: Idiopathic Inflammatory Myopathies; ASS: Anti-Synthetase Syndrome; FVC: Forced Vital Capacity; DLCO: Diffusing Capacity of the Lung for Carbon Monoxide; UIP: Usual Interstitial Pneumonia; OR: Odds Ratio; SSc: Systemic Sclerosis; RR: Relative Risk; RA: Rheumatoid Arthritis; KL-6: Krebs von den Lungen-6; TLC: Total Lung Capacity; CC16: Club Cell Protein 16; IS: Immunosuppressive; HR: Hazard Ratio; TLV: Total Lung Volume; CX3CL1: Chemokine (C-X3-C motif) Ligand 1; ANA: Antinuclear Antibodies; CRP: C-Reactive Protein; CCP: Cyclic Citrullinated Peptide; CYC: Cyclophosphamide; MTX: Methotrexate; MCTD: Mixed Connective Tissue Disease; CCL18: Chemokine (C-C motif) ligand 18; ESR: Erythrocyte Sedimentation Rate; RTX: Rituximab; HRCT: High-Resolution Computed Tomography; Anti-Ro: PFT: Pulmonary Function Test; Anti-dsDNA: Anti-double stranded DNA; Anti-Scl-PM: Anti-Scleroderma-70/Polymyositis; LEF: Leflunomide; RF-IgA: Rheumatoid Factor Immunoglobulin A; HAQ-DI: Health Assessment Questionnaire Disability Index; GGO: Ground-Glass Opacity; DAS28-ESR: Disease Activity Score 28 - Erythrocyte Sedimentation Rate; LDH: Lactate Dehydrogenase; PSS: Primary Sjögren's Syndrome; HTN: Hypertension; IHD: Ischemic Heart Disease; CHF: Congestive Heart Failure; ANCA: Anti-Neutrophil Cytoplasmic Antibodies; PF-ILD: Progressive Fibrosing Interstitial Lung Disease; IgG: Immunoglobulin G; TAC: Tacrolimus; DMARD: Disease-Modifying Antirheumatic Drug; CEA: Carcinoembryonic Antigen; CADM: Clinically Amyopathic Dermatomyositis; ALT: Alanine Aminotransferase; AST: Aspartate Aminotransferase; NSE: Neuron-Specific Enolase; WDFY4 rs7919656: WD Repeat and FYVE Domain Containing 4, rs7919656; MUC5B: Mucin 5B; Alb: Albumin

## **Supplementary Table 2: Quality Evaluation of the Included Studies**

| **First author** | **Representativeness of the Exposed Cohort** | **Selection of the Non-Exposed Cohort** | **Ascertainment of Exposure** | **Demonstration That Outcome of Interest Was Not Present at Start of Study** | **Comparability of Cohorts on the Basis of the Design or Analysis** | **Assessment of Outcome** | **Was Follow-Up Long Enough for Outcomes to Occur** | **Adequacy of Follow Up of Cohorts** | **Total**  **score** |
| --- | --- | --- | --- | --- | --- | --- | --- | --- | --- |
| **I. MARIE 2013** | 1 | 1 | 1 | 1 | 1 | 1 | 1 | 1 | 8 |
| **Giacomo De Luca 2015** | 1 | 1 | 1 | 1 | 2 | 1 | 1 | 1 | 9 |
| **Young Seok Lee 2016** | 1 | 1 | 1 | 1 | 1 | 1 | 1 | 1 | 8 |
| **Tomoyuki Fujisawa 2016** | 1 | 1 | 1 | 1 | 1 | 1 | 1 | 0 | 7 |
| **Sébastien Rivière 2017** | 1 | 1 | 1 | 1 | 1 | 1 | 1 | 1 | 8 |
| **Silje Reiseter 2018** | 1 | 1 | 1 | 1 | 0 | 1 | 1 | 1 | 7 |
| **Anna-Maria Hoffmann-Vold 2018** | 1 | 1 | 1 | 1 | 1 | 1 | 1 | 1 | 8 |
| **Tatiana Cobo-Ibáñez 2019** | 1 | 1 | 1 | 1 | 2 | 1 | 1 | 1 | 9 |
| **Hui Liu 2019** | 1 | 1 | 1 | 1 | 1 | 1 | 0 | 0 | 6 |
| **Qiang Fu 2019** | 1 | 1 | 1 | 1 | 2 | 1 | 1 | 0 | 8 |
| **Takeshi Mochizuki 2019** | 1 | 1 | 1 | 1 | 2 | 1 | 1 | 1 | 9 |
| **Muriel Elhai 2019** | 1 | 1 | 1 | 1 | 2 | 1 | 1 | 1 | 9 |
| **Luling Li A 2019** | 1 | 1 | 1 | 1 | 2 | 1 | 1 | 1 | 9 |
| **Luling Li 2020** | 1 | 1 | 1 | 1 | 2 | 1 | 1 | 0 | 8 |
| **Cristina Vadillo 2020** | 1 | 1 | 1 | 1 | 2 | 1 | 1 | 1 | 9 |
| **Yuetong Xu 2021** | 1 | 1 | 1 | 1 | 2 | 1 | 1 | 1 | 9 |
| **Yu-Hsiang Chiu 2021** | 1 | 1 | 1 | 1 | 2 | 1 | 1 | 1 | 9 |
| **Anna-Maria Hoffmann-Vold 2021** | 1 | 1 | 1 | 1 | 1 | 1 | 1 | 1 | 8 |
| **Natalia Mena-Vázquez 2021** | 1 | 1 | 1 | 1 | 2 | 1 | 1 | 1 | 9 |
| **Satoshi Watanabe 2022** | 1 | 1 | 1 | 1 | 2 | 1 | 1 | 0 | 8 |
| **Ning Chen 2022** | 1 | 1 | 1 | 1 | 0 | 1 | 1 | 0 | 6 |
| **Lei Liu 2022** | 1 | 1 | 1 | 1 | 2 | 1 | 1 | 1 | 9 |
| **Na Zhao 2022** | 1 | 1 | 1 | 1 | 2 | 1 | 1 | 1 | 9 |
| **Shi‑Hao He 2022** | 1 | 1 | 1 | 1 | 2 | 1 | 1 | 0 | 8 |
| **Ju Kwang Lee 2023** | 1 | 1 | 1 | 1 | 1 | 1 | 1 | 1 | 8 |
| **Dandan Chai 2023** | 1 | 1 | 1 | 1 | 1 | 1 | 1 | 0 | 7 |
| **Gonçalo Boleto 2023** | 1 | 1 | 1 | 1 | 1 | 1 | 1 | 1 | 8 |
| **Kyung‑Ann Lee 2023** | 1 | 1 | 1 | 1 | 1 | 1 | 1 | 1 | 8 |
| **Kyuhwan Kim 2023** | 1 | 1 | 1 | 1 | 2 | 1 | 1 | 1 | 9 |
| **Yu-Hsuan Chen 2023** | 1 | 1 | 1 | 1 | 2 | 1 | 1 | 0 | 8 |
| **Wen Zeng 2023** | 1 | 1 | 1 | 1 | 1 | 1 | 1 | 1 | 8 |
| **Ji-Won Kim 2023** | 1 | 1 | 1 | 1 | 1 | 1 | 1 | 1 | 8 |
| **Y. Xu 2016** | 1 | 1 | 1 | 1 | 1 | 1 | 1 | 1 | 8 |
| **Kohei Karino 2020** | 1 | 1 | 1 | 1 | 1 | 1 | 1 | 1 | 8 |
| **Qihua Yang 2021** | 1 | 1 | 1 | 1 | 1 | 1 | 1 | 1 | 8 |
| **Dingxian Zhu 2021** | 1 | 1 | 1 | 1 | 1 | 1 | 1 | 1 | 8 |
| **Yu Zuo 2022** | 1 | 1 | 1 | 1 | 1 | 1 | 1 | 0 | 7 |
| **Y. Zhu 2022** | 1 | 1 | 1 | 1 | 1 | 1 | 1 | 1 | 8 |
| **Hanxiao You 2023** | 1 | 1 | 1 | 1 | 0 | 1 | 1 | 0 | 6 |
| **Li Guo 2023** | 1 | 1 | 1 | 1 | 1 | 1 | 1 | 1 | 8 |
| **Haoru Zhang 2023** | 1 | 1 | 1 | 1 | 1 | 1 | 1 | 1 | 8 |
| **Zou Ruyi 2023** | 1 | 1 | 1 | 1 | 1 | 1 | 1 | 1 | 8 |
| **Lu Cheng 2023** | 1 | 1 | 1 | 1 | 2 | 1 | 1 | 1 | 9 |
| **HAN Yuan-yuan 2023** | 1 | 1 | 1 | 1 | 2 | 1 | 1 | 1 | 9 |
| **Wang Yafei 2023** | 1 | 1 | 1 | 1 | 1 | 1 | 1 | 1 | 8 |
| **Chengyin Lv 2023** | 1 | 1 | 1 | 1 | 1 | 1 | 1 | 1 | 8 |
| **Lei Wang 2024** | 1 | 1 | 1 | 1 | 1 | 1 | 1 | 1 | 8 |
| **Takafumi Suda 2009** | 1 | 1 | 1 | 1 | 1 | 1 | 1 | 1 | 8 |
| **Fumiko Tomiyama 2016** | 1 | 1 | 1 | 1 | 1 | 1 | 1 | 1 | 8 |
| **Mengshu Cao 2019** | 1 | 1 | 1 | 1 | 1 | 1 | 1 | 1 | 8 |
| **Shinji Izuka 2021** | 1 | 1 | 1 | 1 | 1 | 1 | 1 | 1 | 8 |
| **Na Wang 2020** | 1 | 1 | 1 | 1 | 1 | 1 | 1 | 1 | 8 |
| **Nozomi Tanaka 2021** | 1 | 1 | 1 | 1 | 1 | 1 | 1 | 1 | 8 |
| **Byoung Soo Kwon 2022** | 1 | 1 | 1 | 1 | 2 | 1 | 1 | 1 | 9 |
| **Junji Otsuka 2022** | 1 | 1 | 1 | 1 | 2 | 1 | 1 | 1 | 9 |

## Meta-analysis results of the progression of CTD-ILD**.**

### **Male**


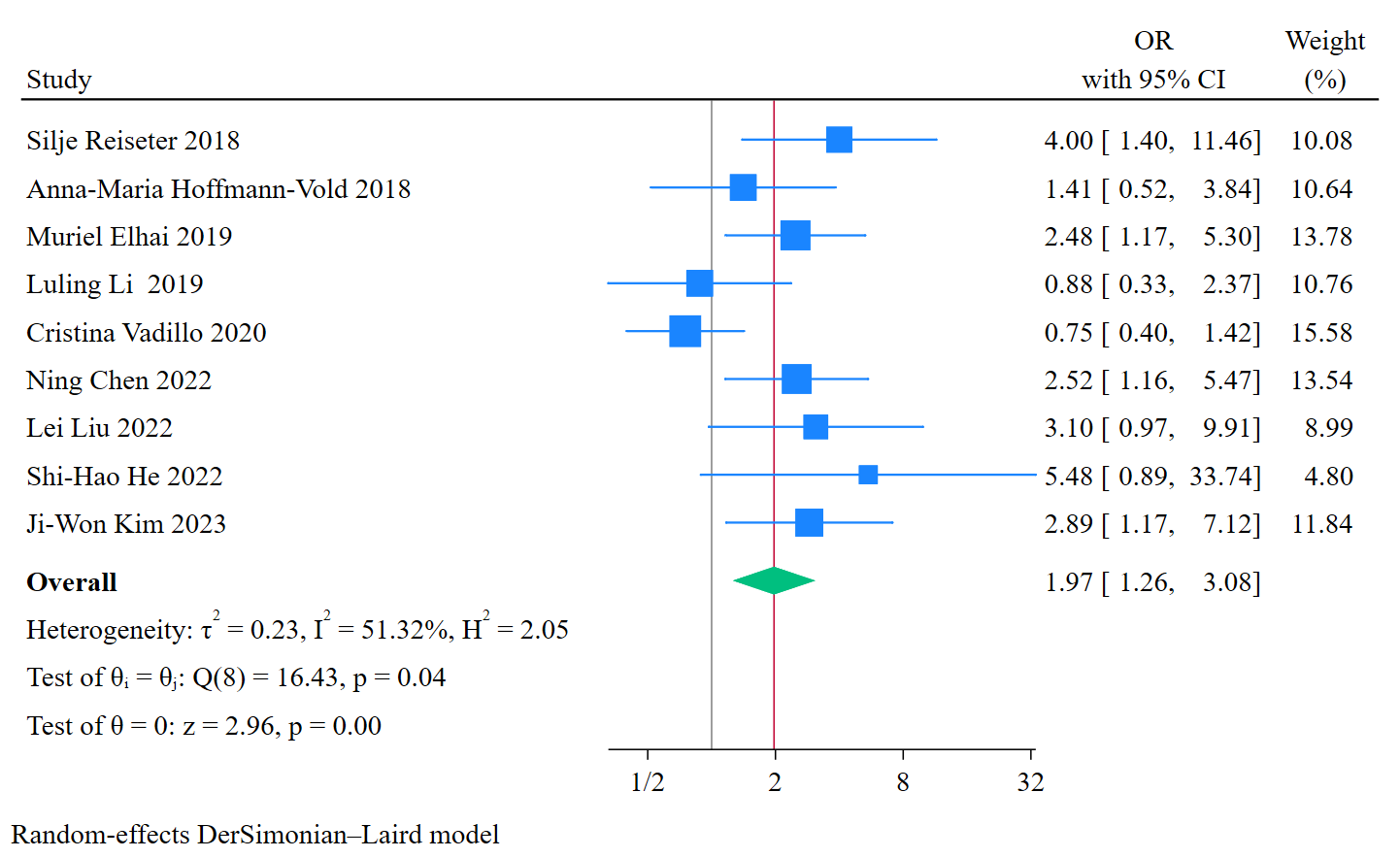


Forest plot for male as a risk factor in the progression of CTD-ILD


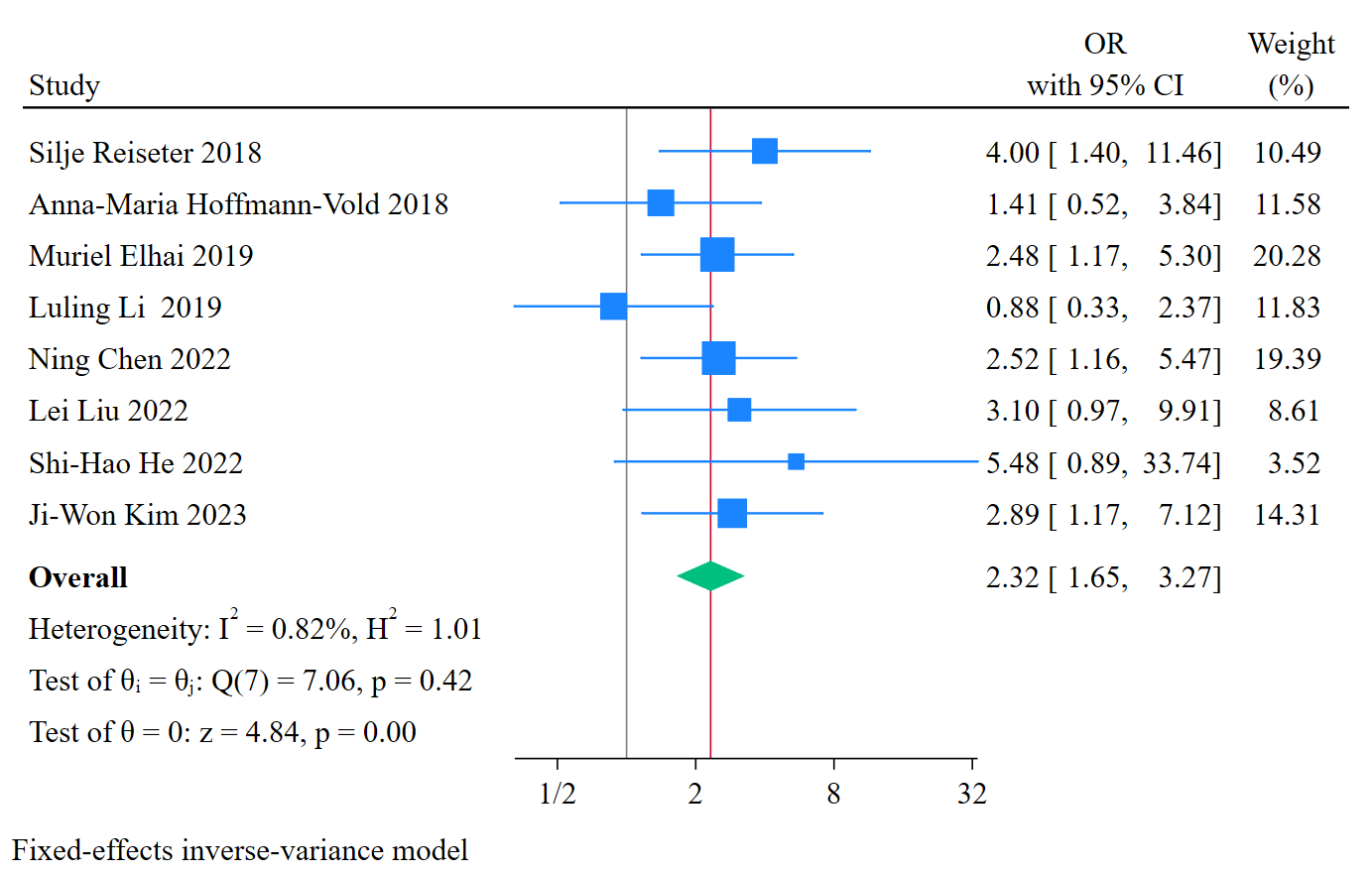


Forest plot for male as a risk factor in the progression of CTD-ILD after excluding one study


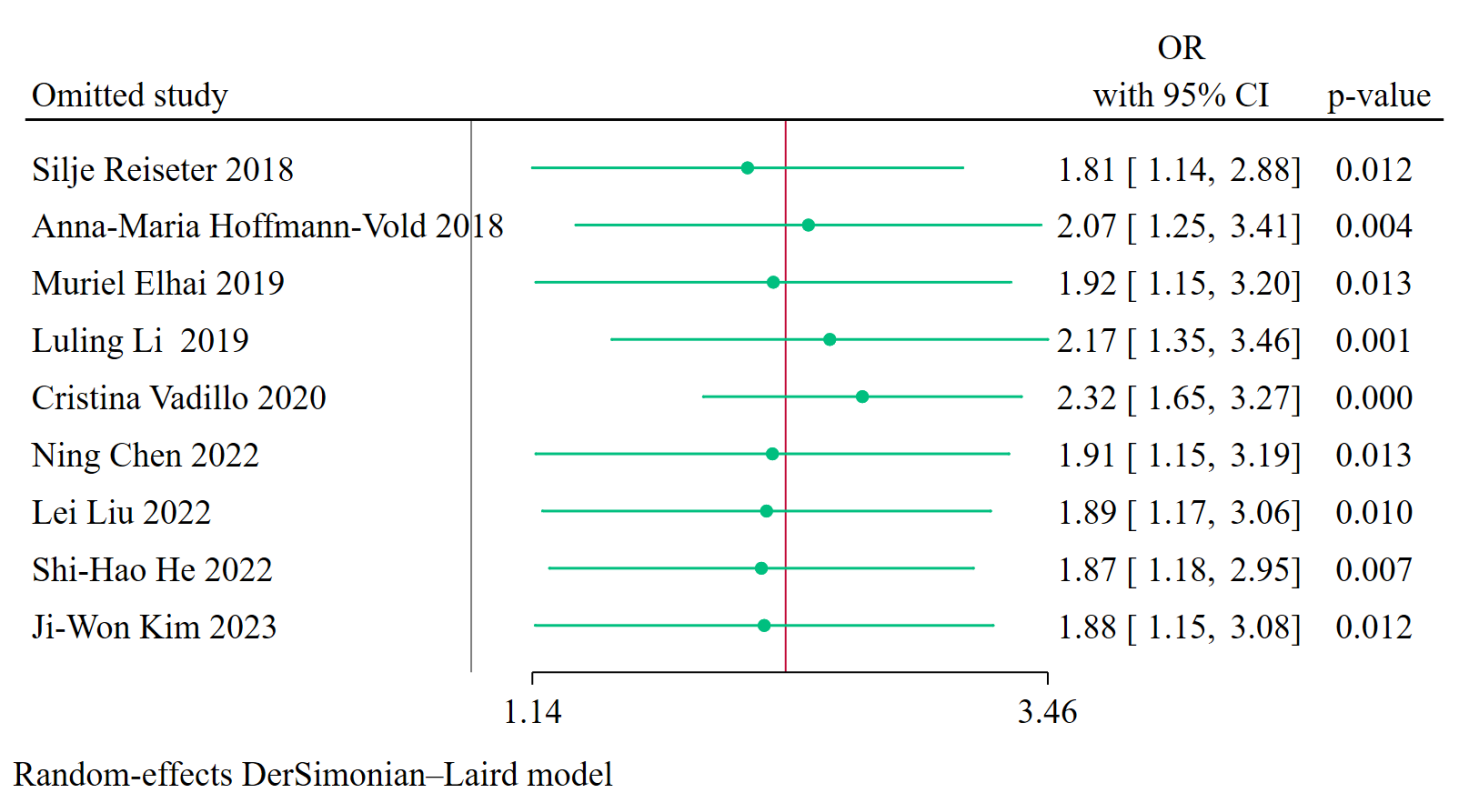


Sensitivity analysis of male as a risk factor for CTD-ILD progression


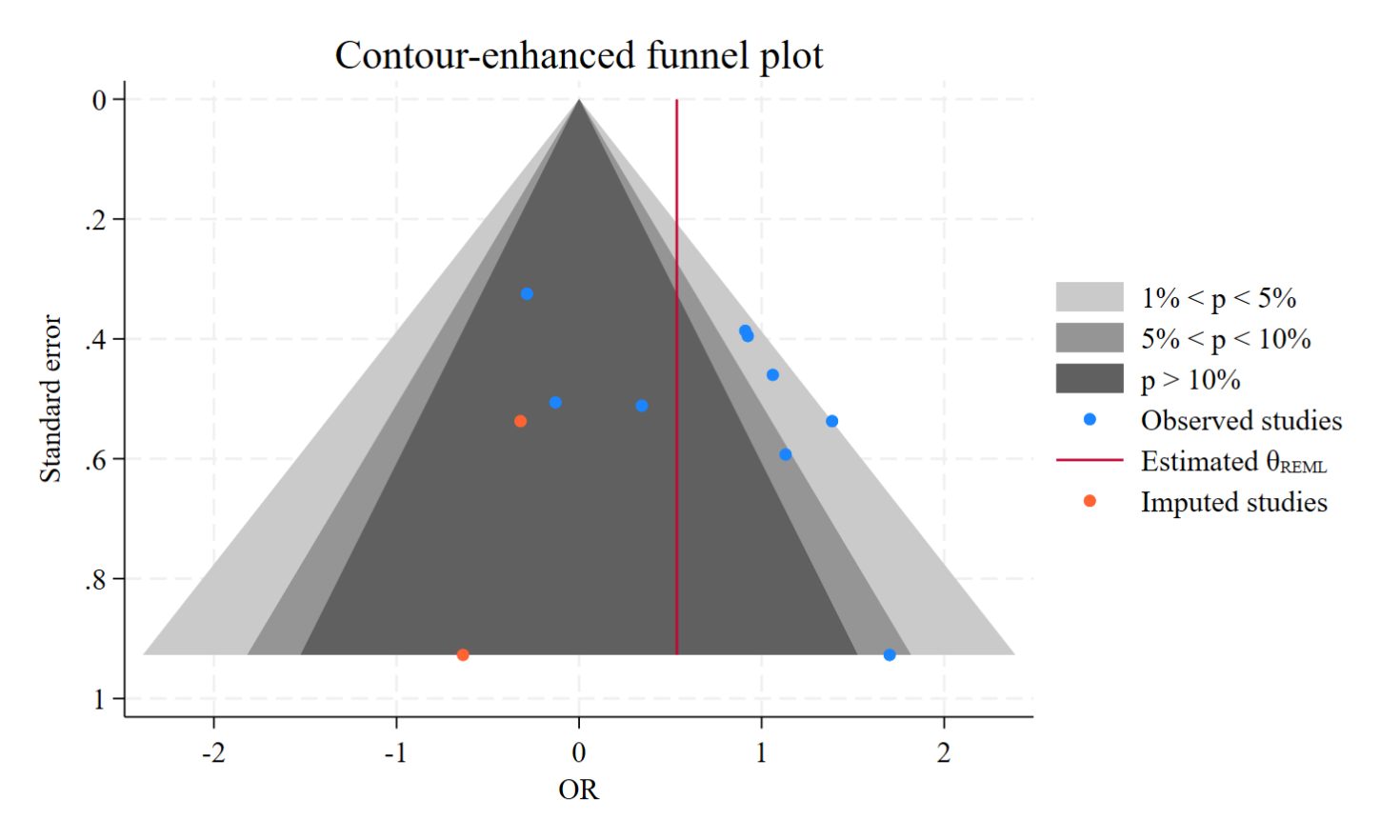


Snip-and-fill funnel plot of male as a risk factor for CTD-ILD progression

### **UIP patterns on HRCT**


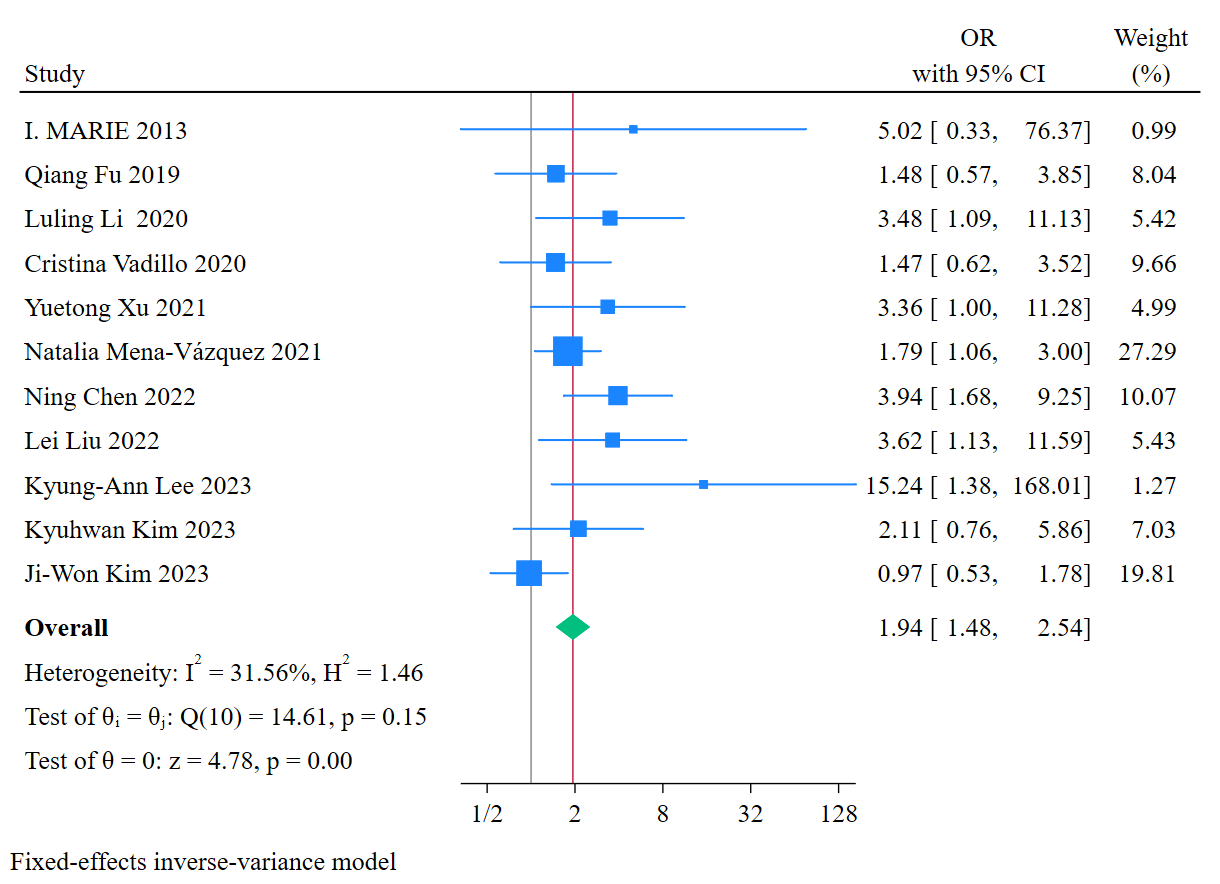


Forest plot for UIP patterns on HRCT as a risk factor in the progression of CTD-ILD


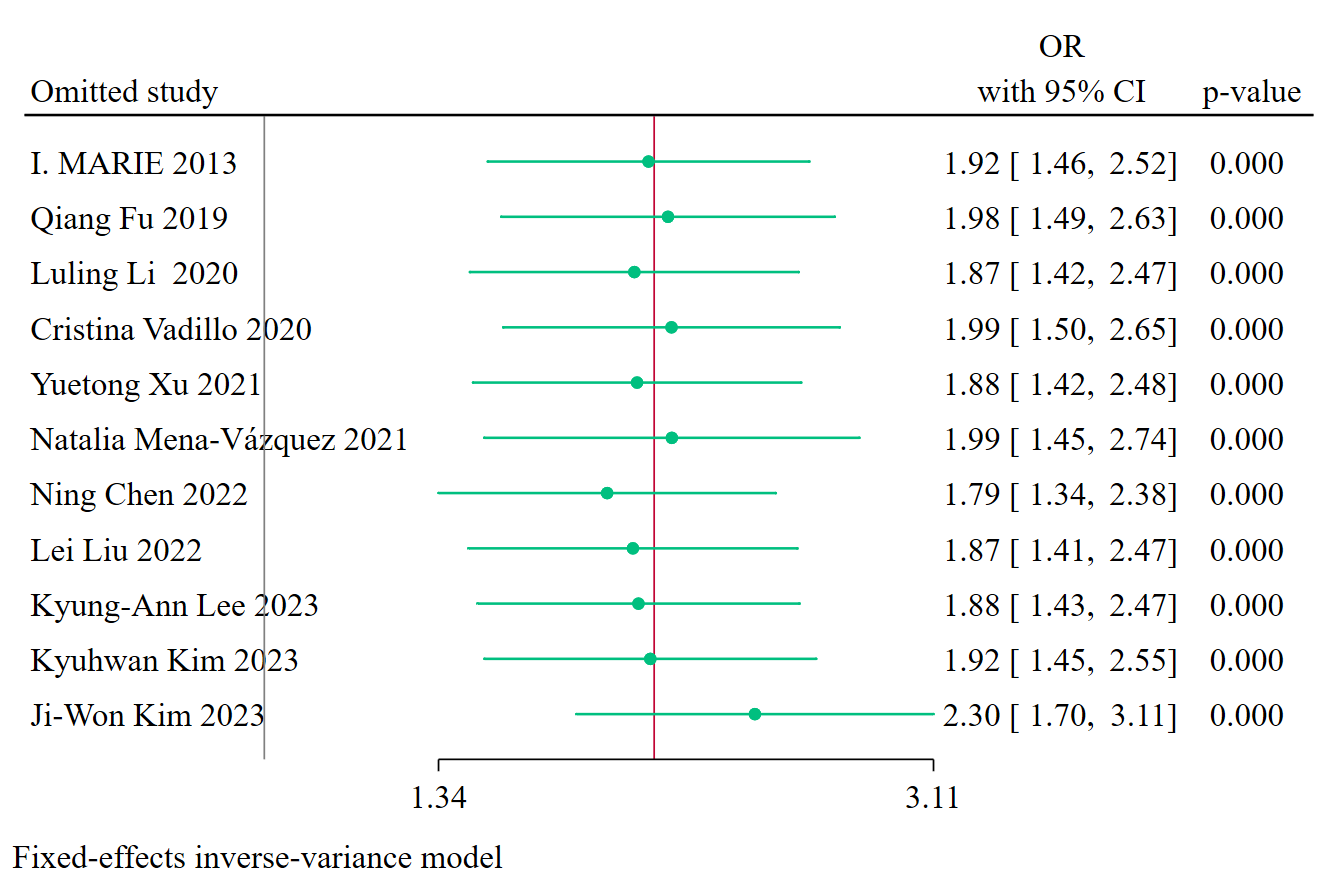


Sensitivity analysis of UIP patterns on HRCT as a risk factor for CTD-ILD progression


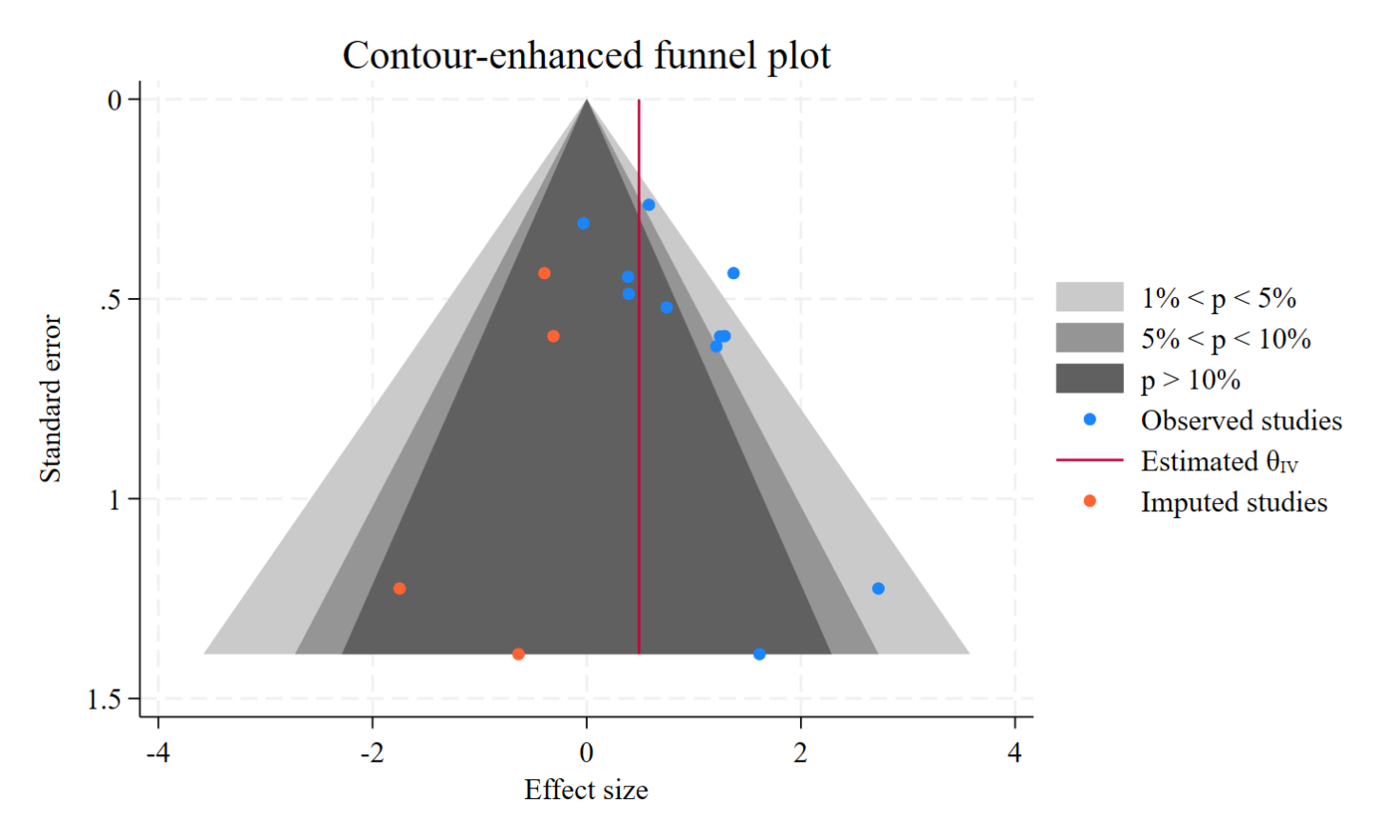


Snip-and-fill funnel plot of UIP patterns on HRCT as a risk factor for CTD-ILD progression

### **extensive lung involvement**


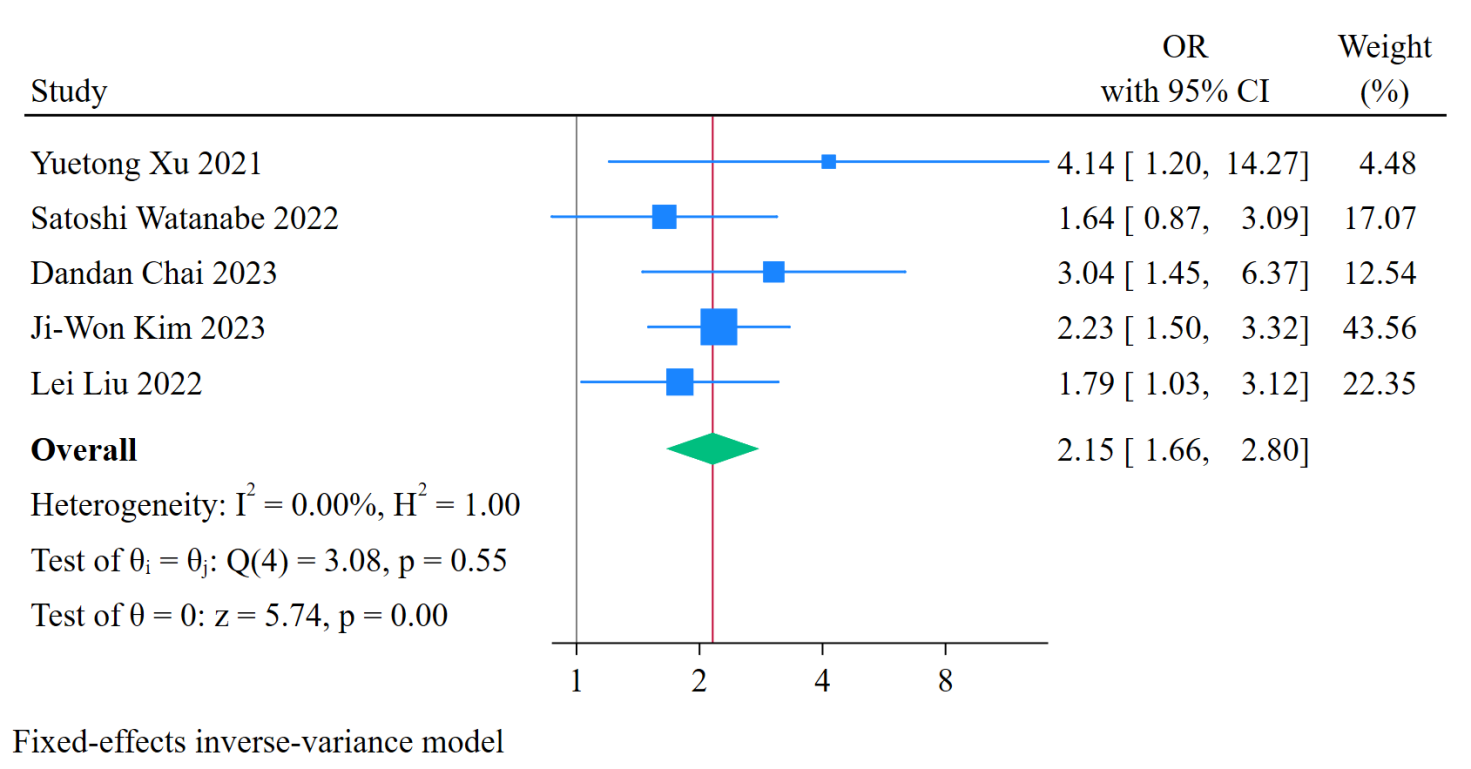


Forest plot for Extensive lung involvement as a risk factor in the progression of CTD-ILD


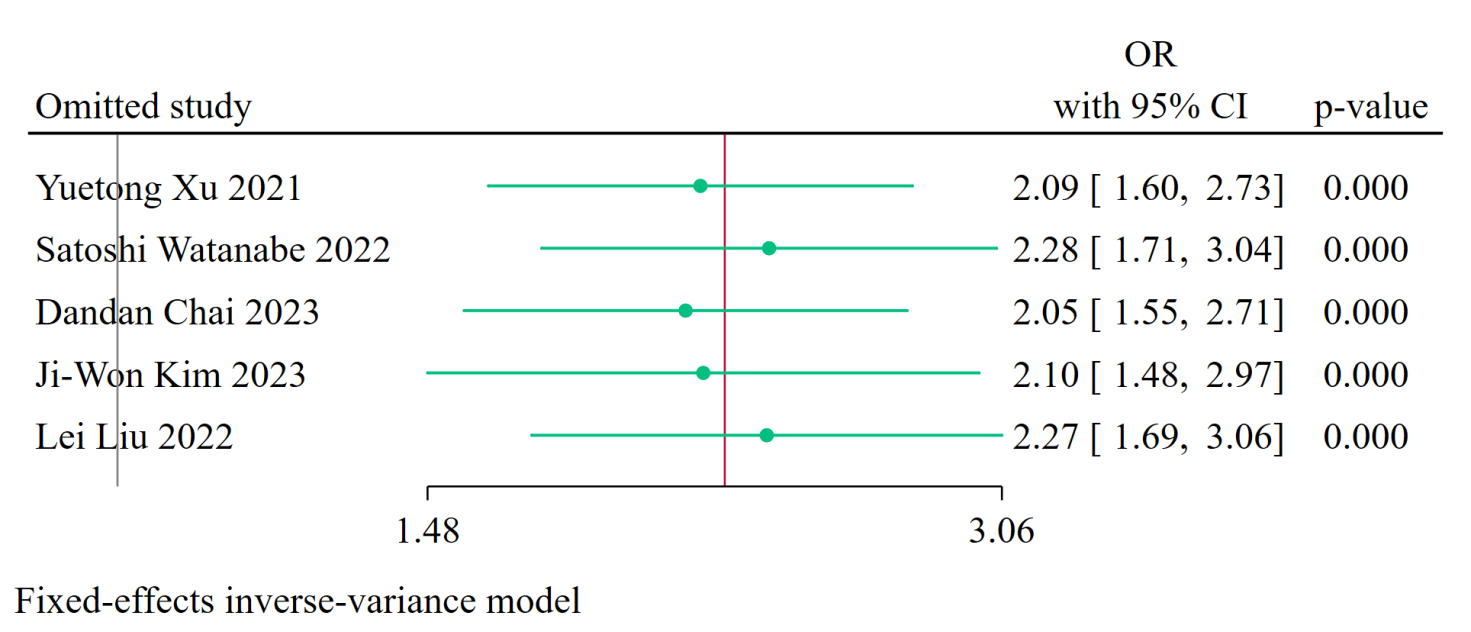


Sensitivity analysis of Extensive lung involvement as a risk factor for CTD-ILD progression


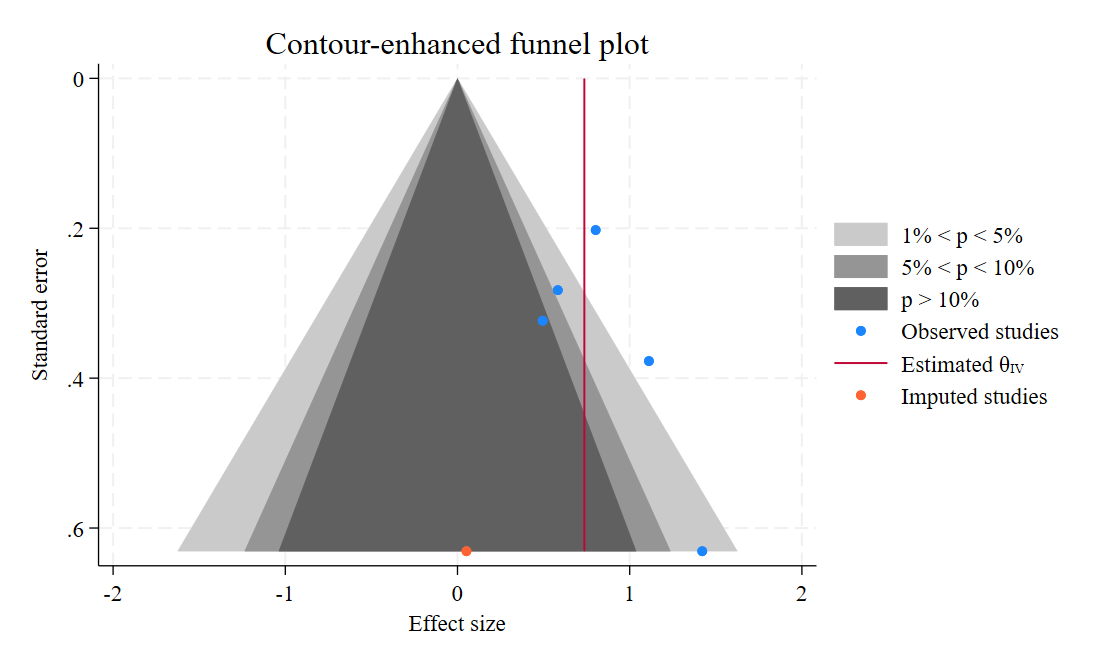


Snip-and-fill funnel plot of Extensive lung involvement as a risk factor for CTD-ILD progression

### **Age**


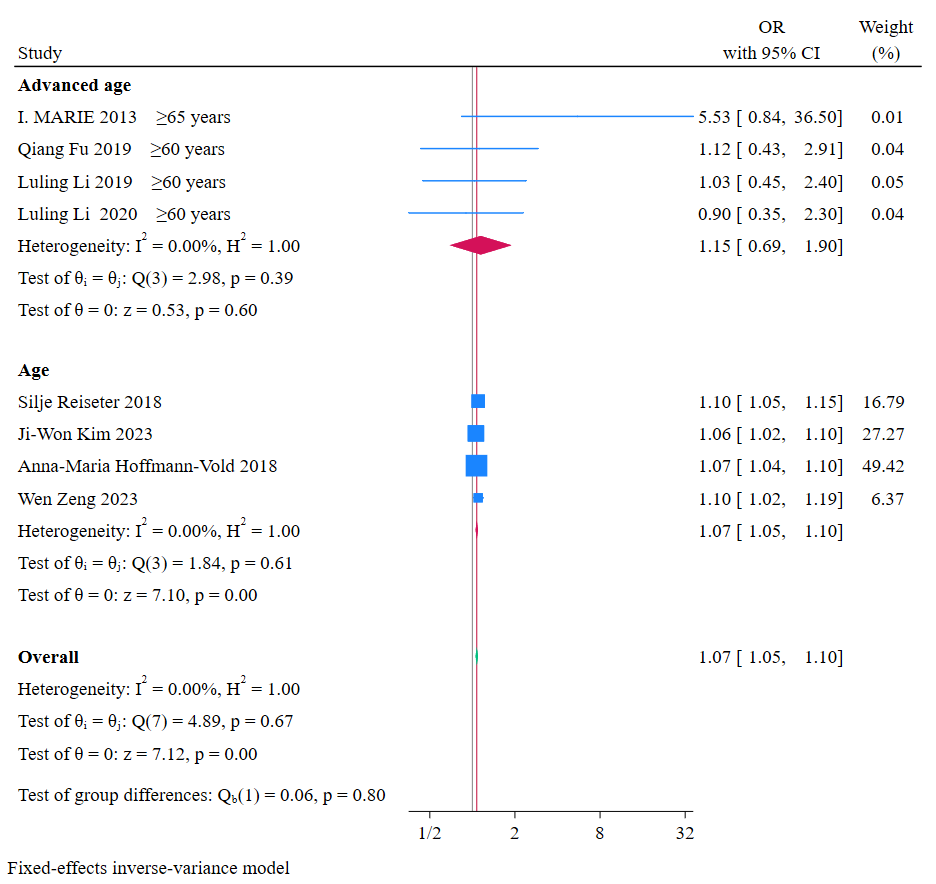


Forest plot for age as a risk factor in the progression of CTD-ILD


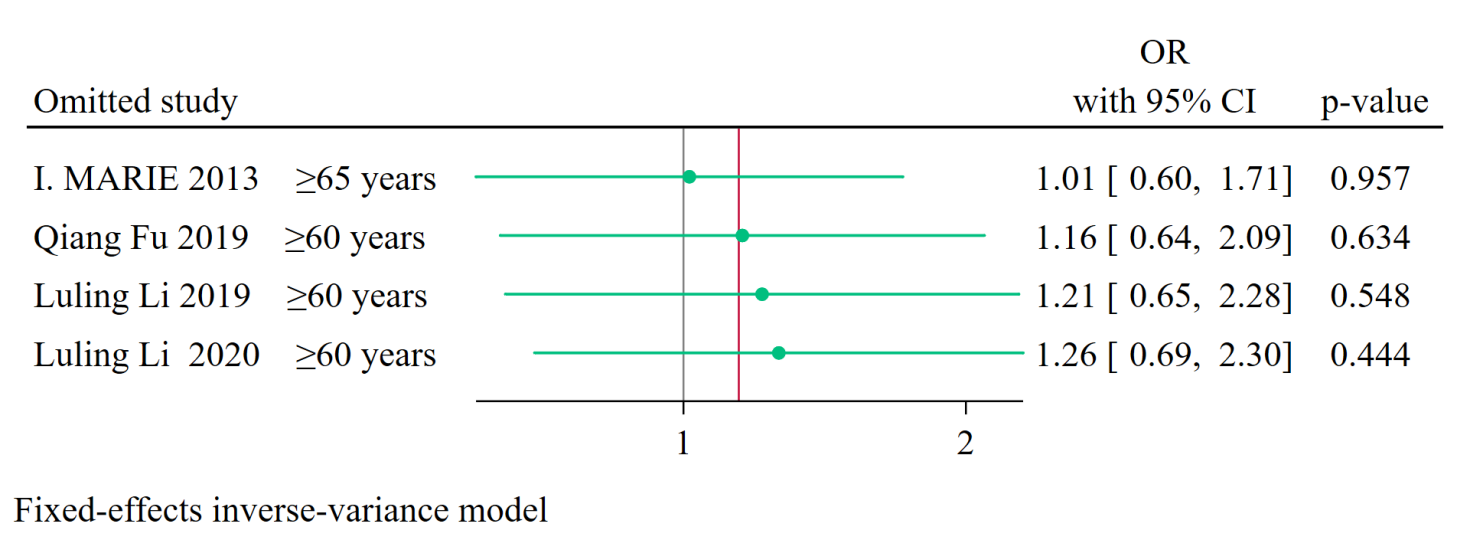


Sensitivity analysis of advanced age as a risk factor for CTD-ILD progression


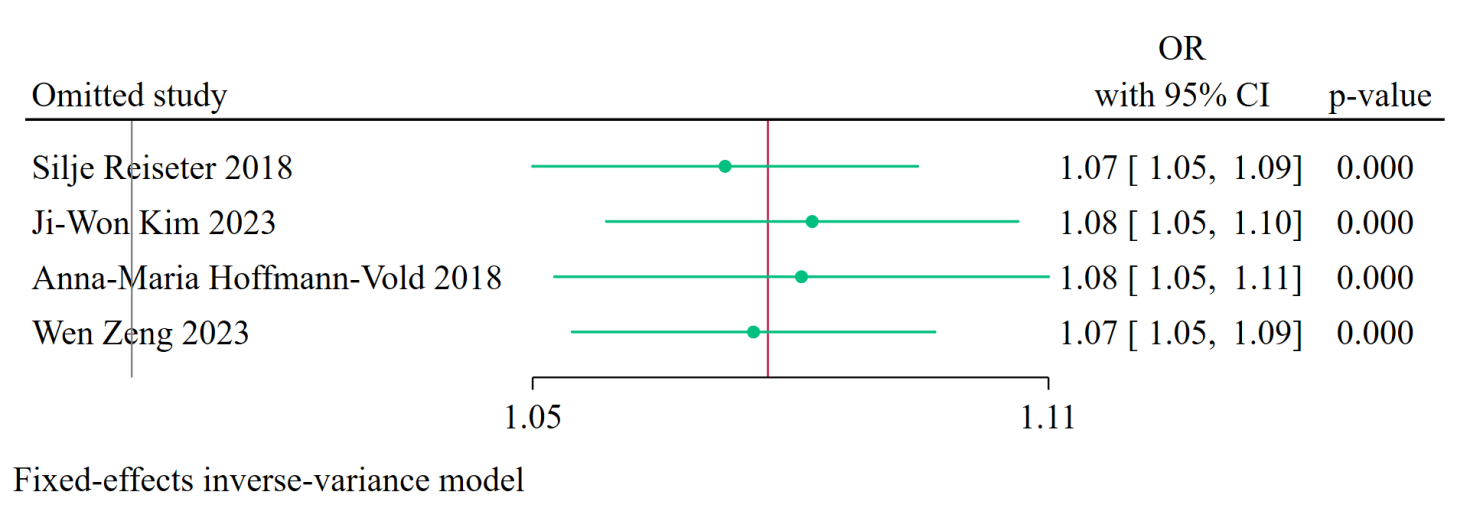


Sensitivity analysis of age as a risk factor for progression

### **FVC**


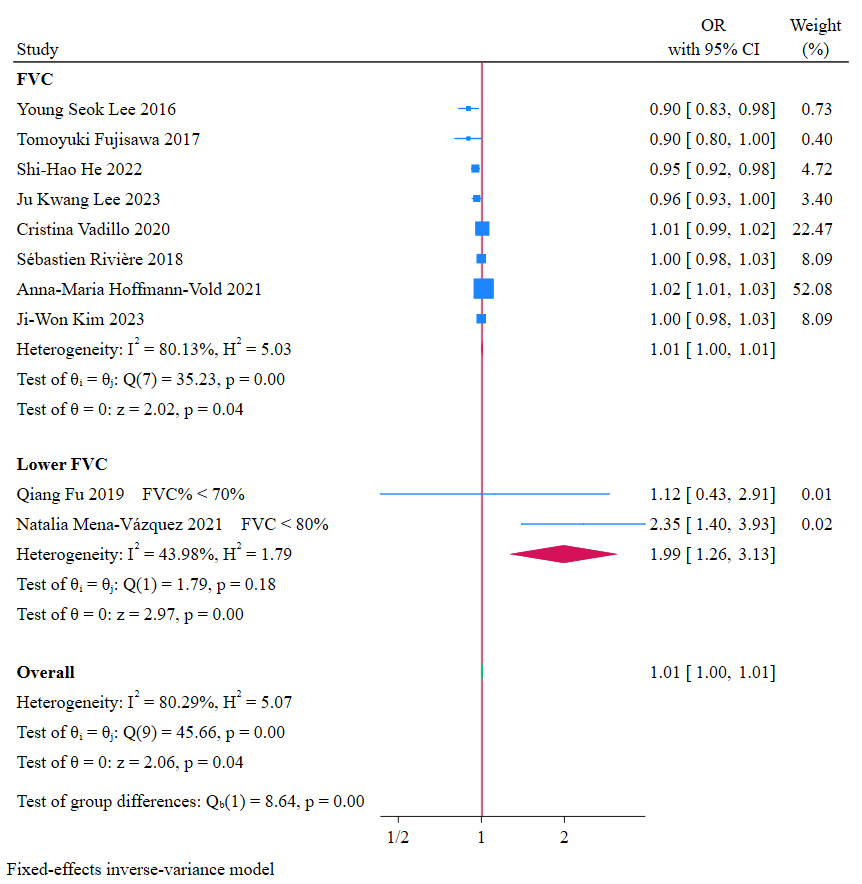


Forest plot for low FVC as a risk factor in the progression of CTD-ILD


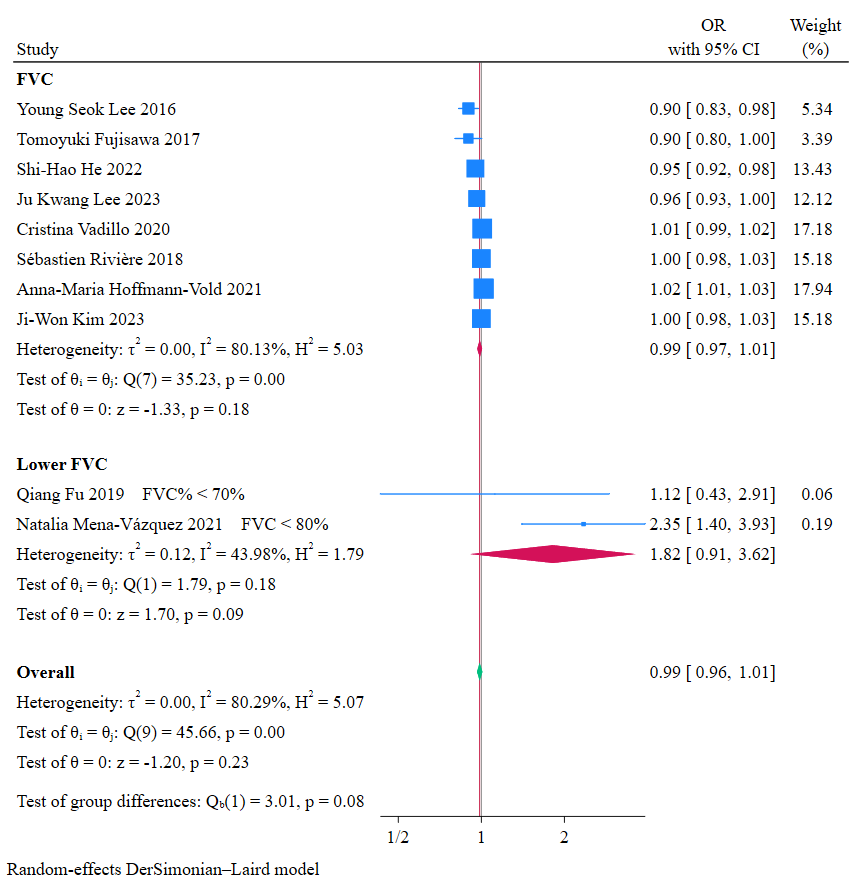


Forest plot for FVC as a risk factor in the progression of CTD-ILD

### **DLCO**


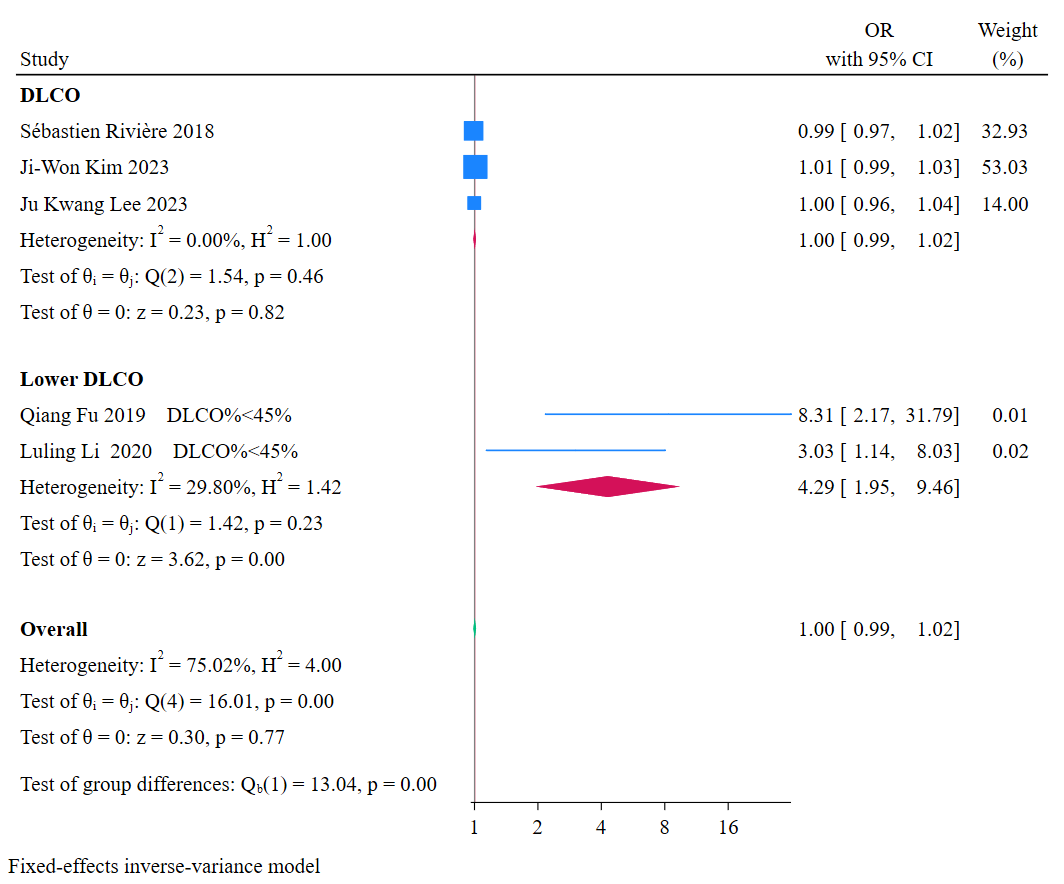


Forest plot for DLCO as a risk factor in the progression of CTD-ILD

### **ANA+**


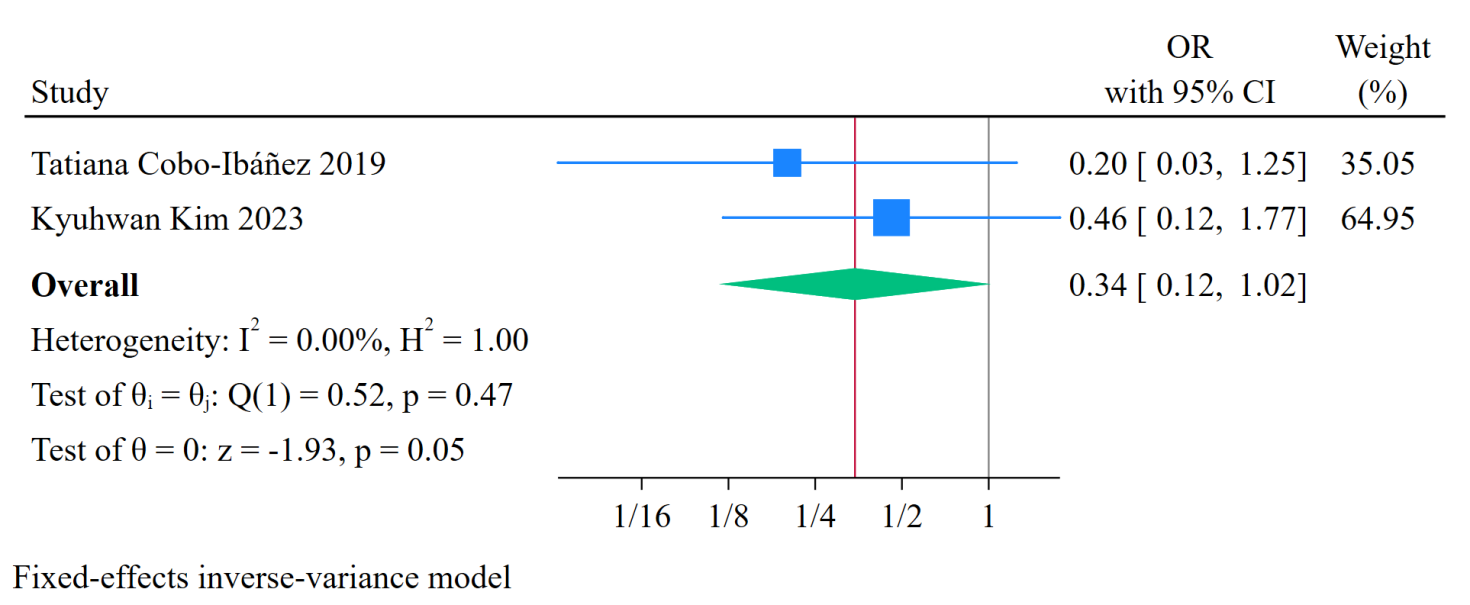


Forest plot for ANA+ as a risk factor in the progression of CTD-ILD

### **ESR**


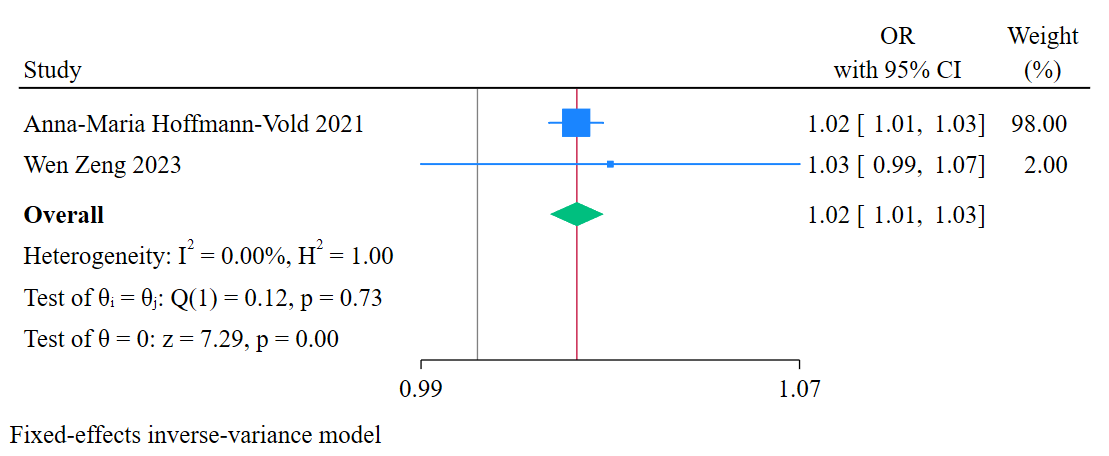


Forest plot for ESR as a risk factor in the progression of CTD-ILD

### **diffuse skin involvement**


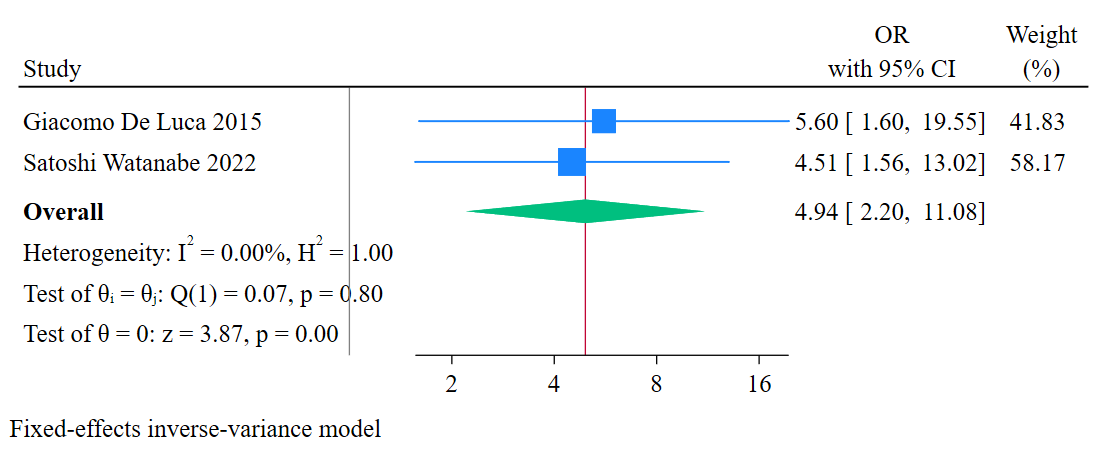


Forest plot for diffuse skin involvement as a risk factor in the progression of CTD-ILD

### **mRSS**


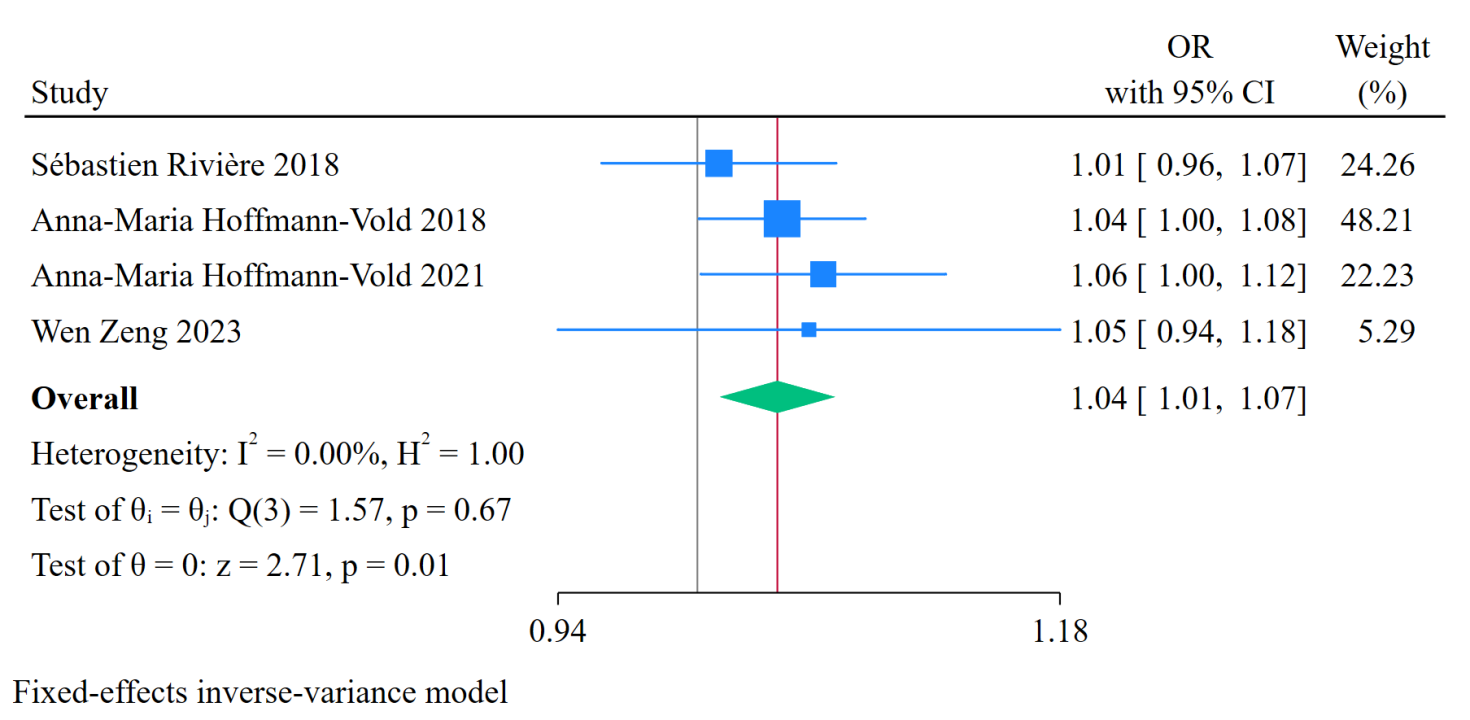


Forest plot for mRSS as a risk factor in the progression of CTD-ILD

### **KL-6**


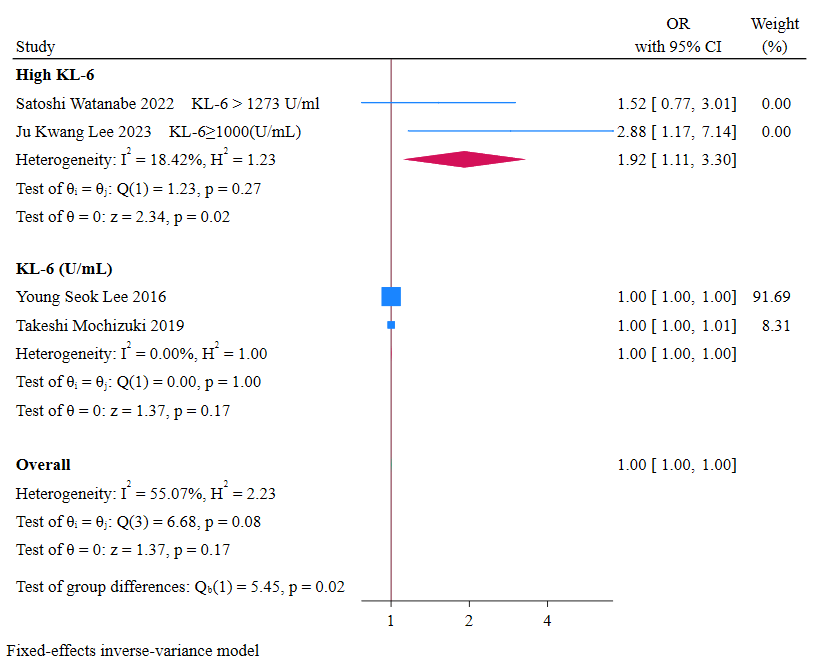


Forest plot for KL-6 as a risk factor in the progression of CTD-ILD

### **RTX**


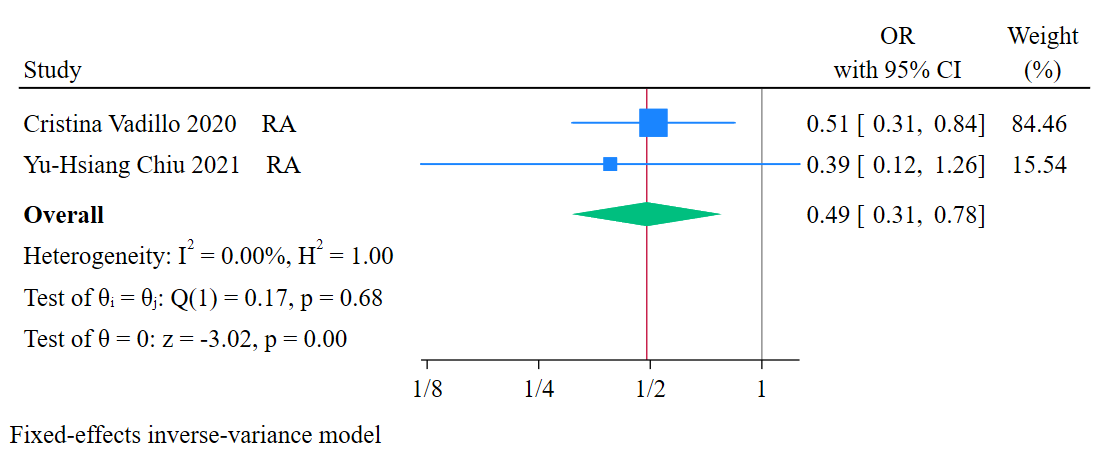


Forest plot for RTX as a risk factor in the progression of CTD-ILD

### **Arthritis**


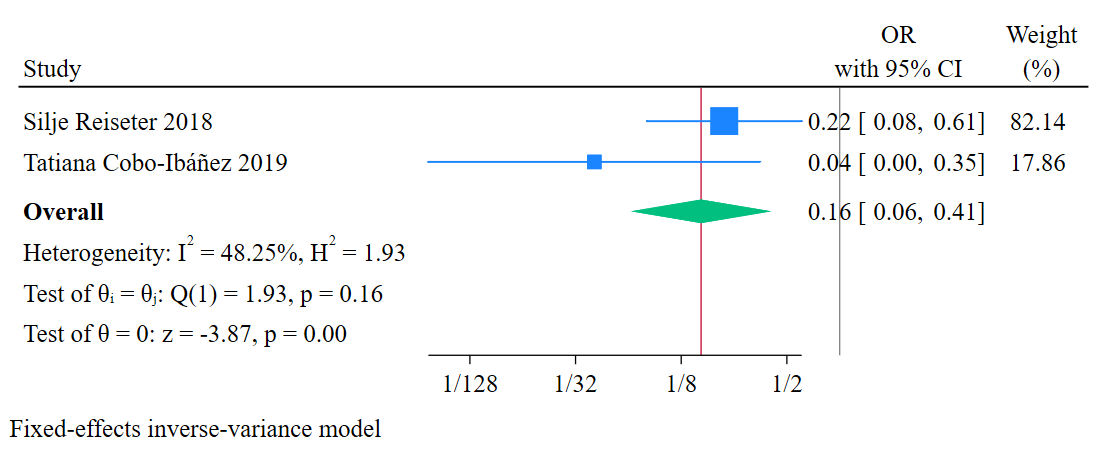


Forest plot for arthritis as a risk factor in the progression of CTD-ILD

### **Dysphagia or reflux**


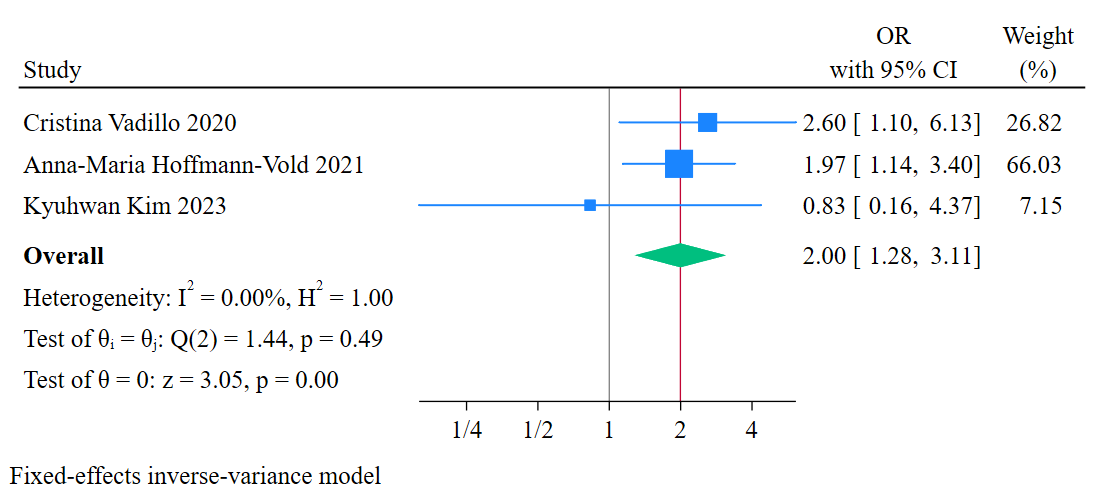


Forest plot for dysphagia or reflux as a risk factor in the progression of CTD-ILD

### **Shortness of breath**


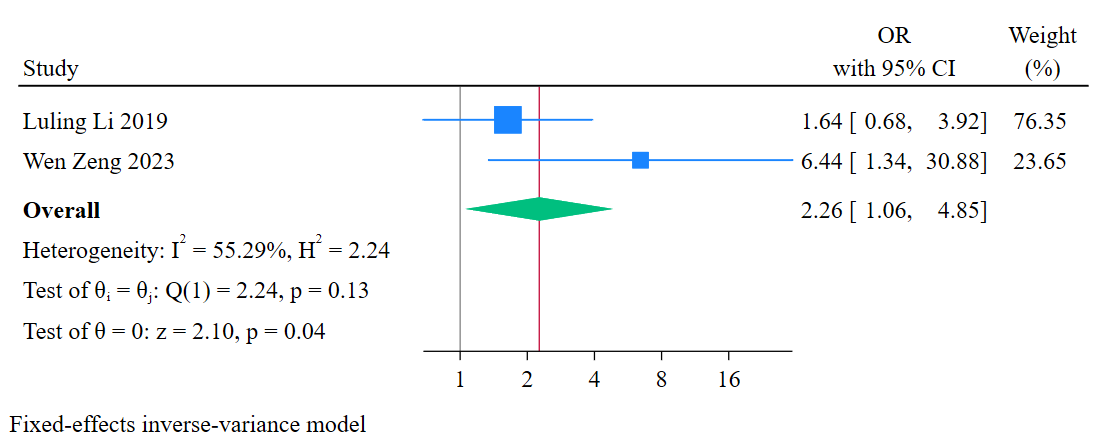


Forest plot for shortness of breath as a risk factor in the progression of CTD-ILD

### **Smoking history**


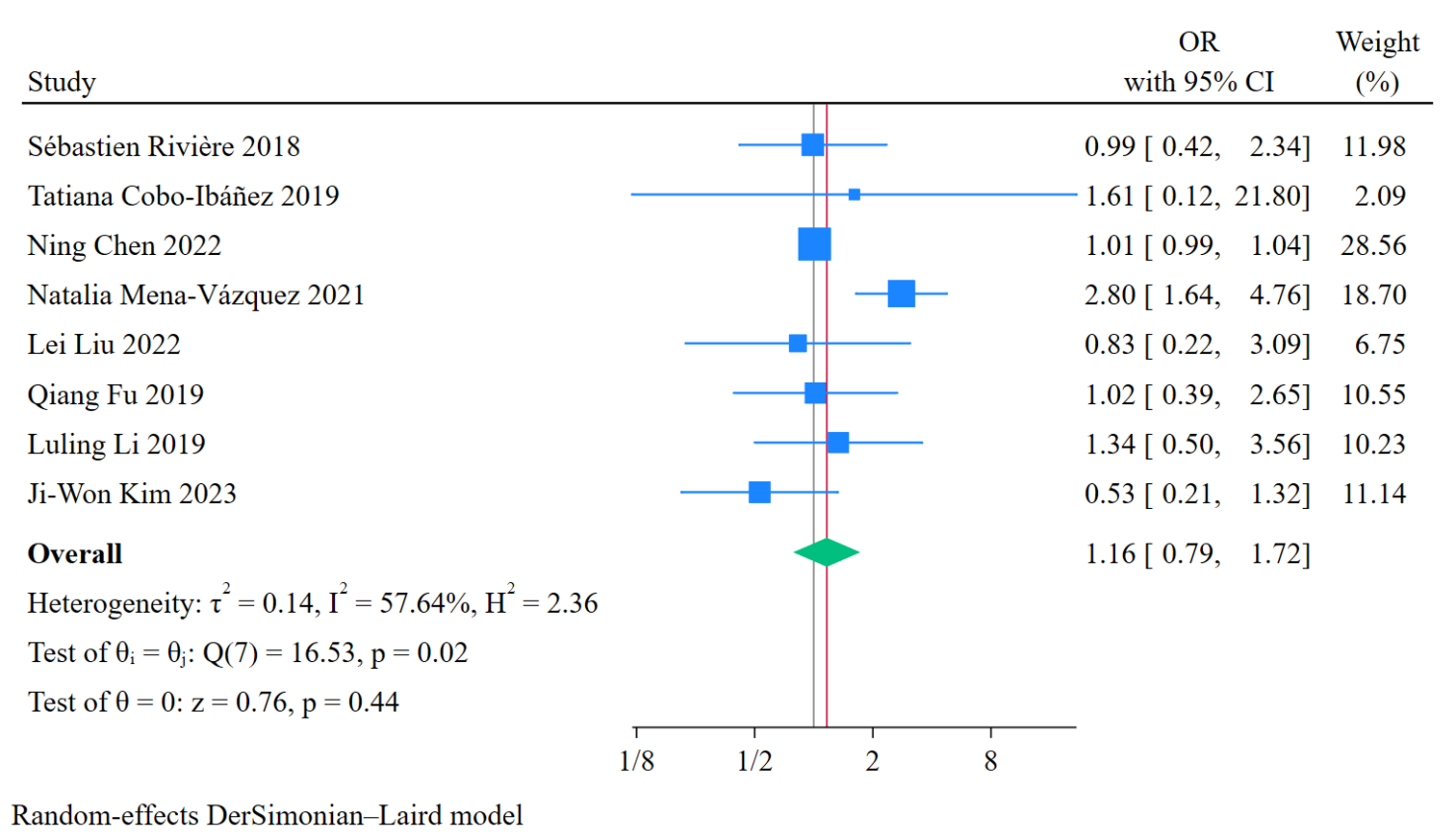


Forest plot for smoking history as a risk factor in the progression of CTD-ILD

### **Disease duration**


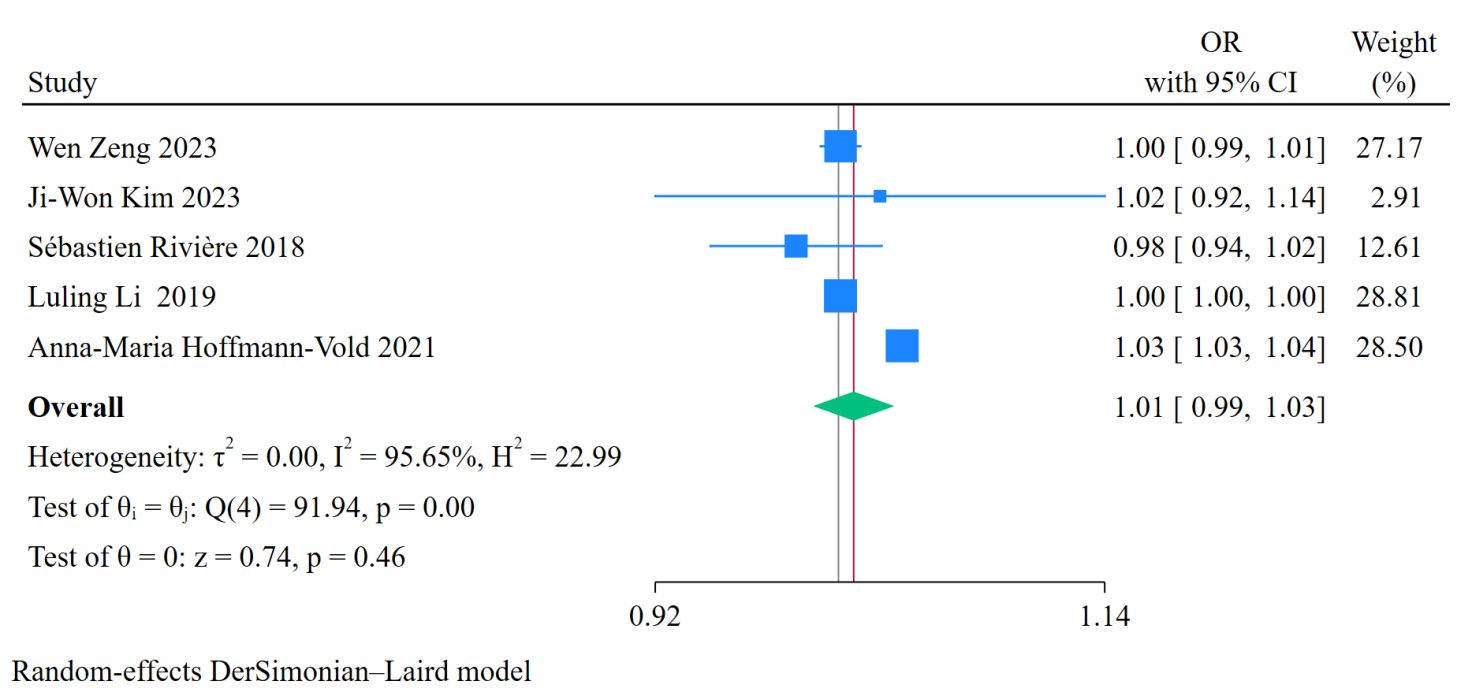


Forest plot for disease duration as a risk factor in the progression of CTD-ILD

### **CCP**


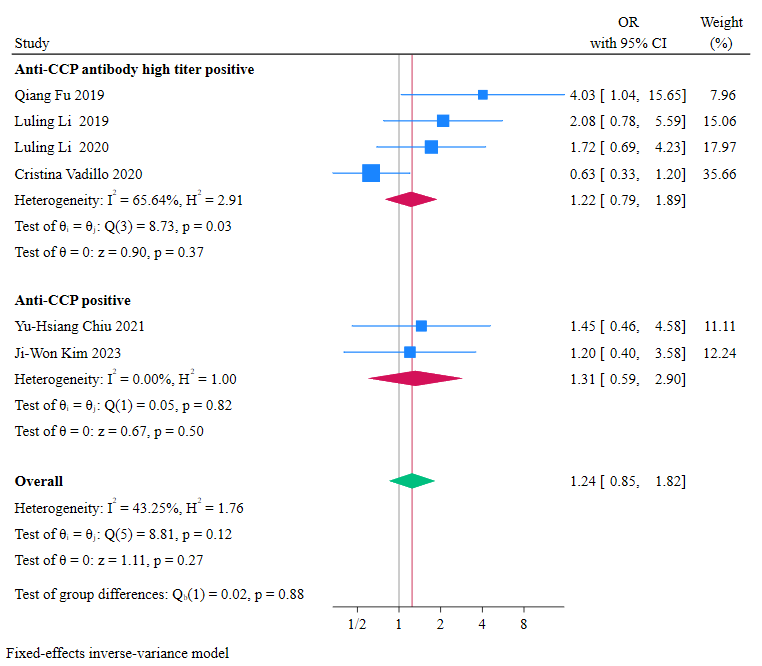


Forest plot for CCP as a risk factor in the progression of CTD-ILD


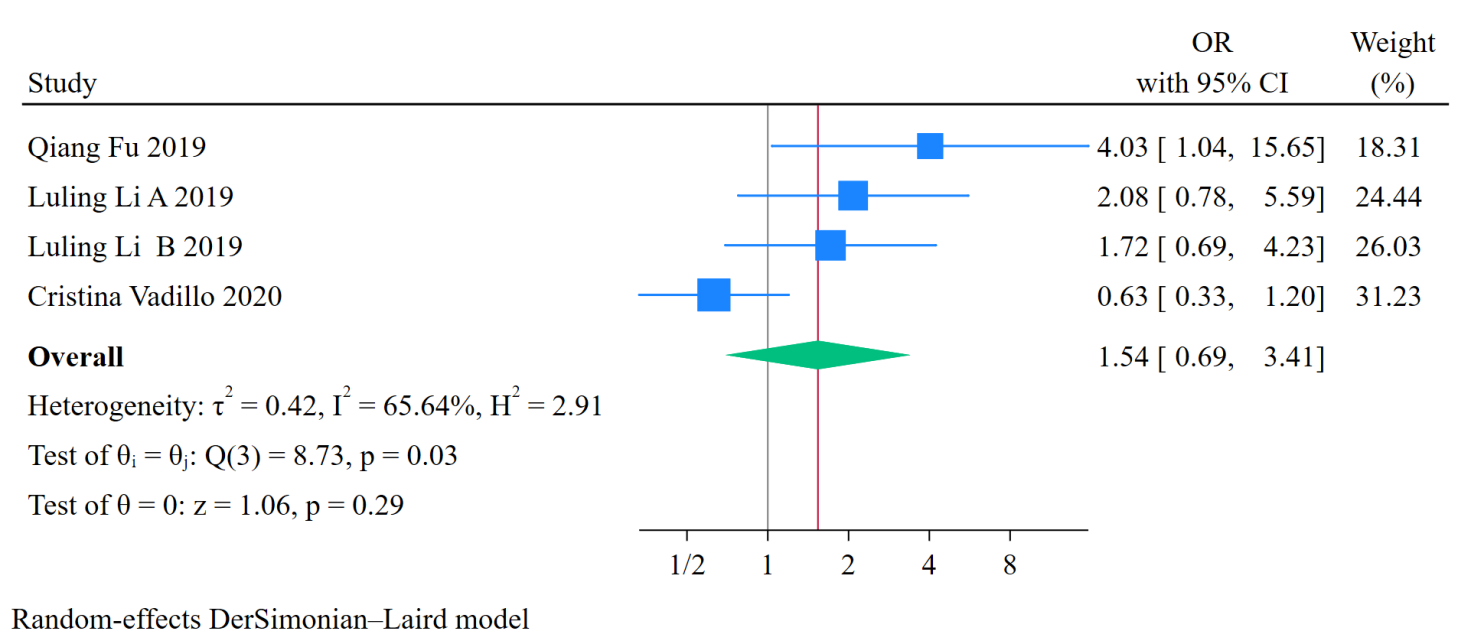


Forest plot for anti-CCP antibody high titer positive as a risk factor in the progression of CTD-ILD

### **CRP**


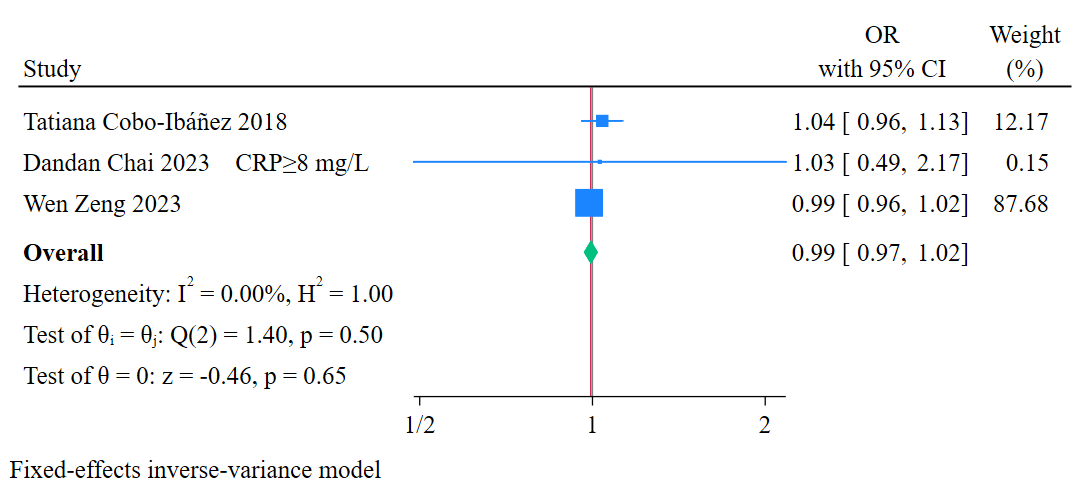


Forest plot for CRP as a risk factor in the progression of CTD-ILD

### **RF**


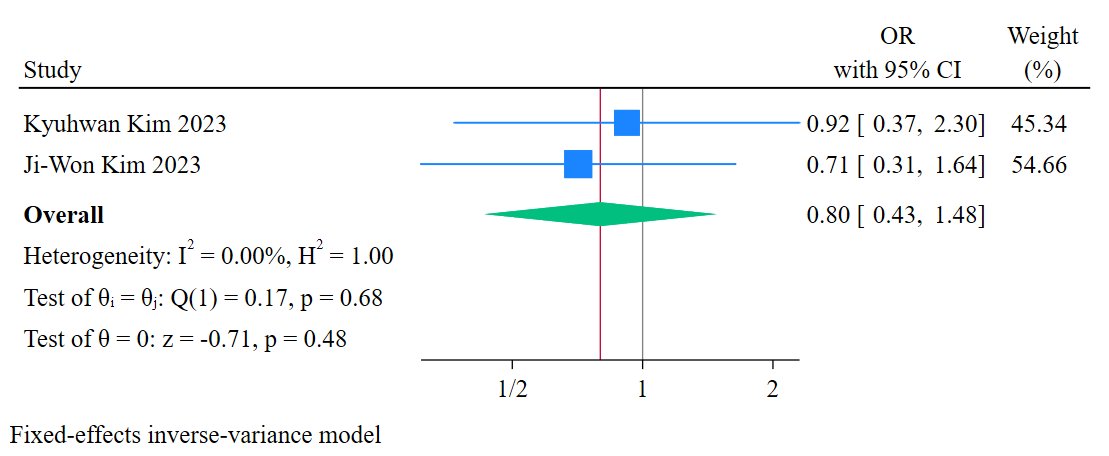


Forest plot for RF as a risk factor in the progression of CTD-ILD

### **Reticulation on HRCT**


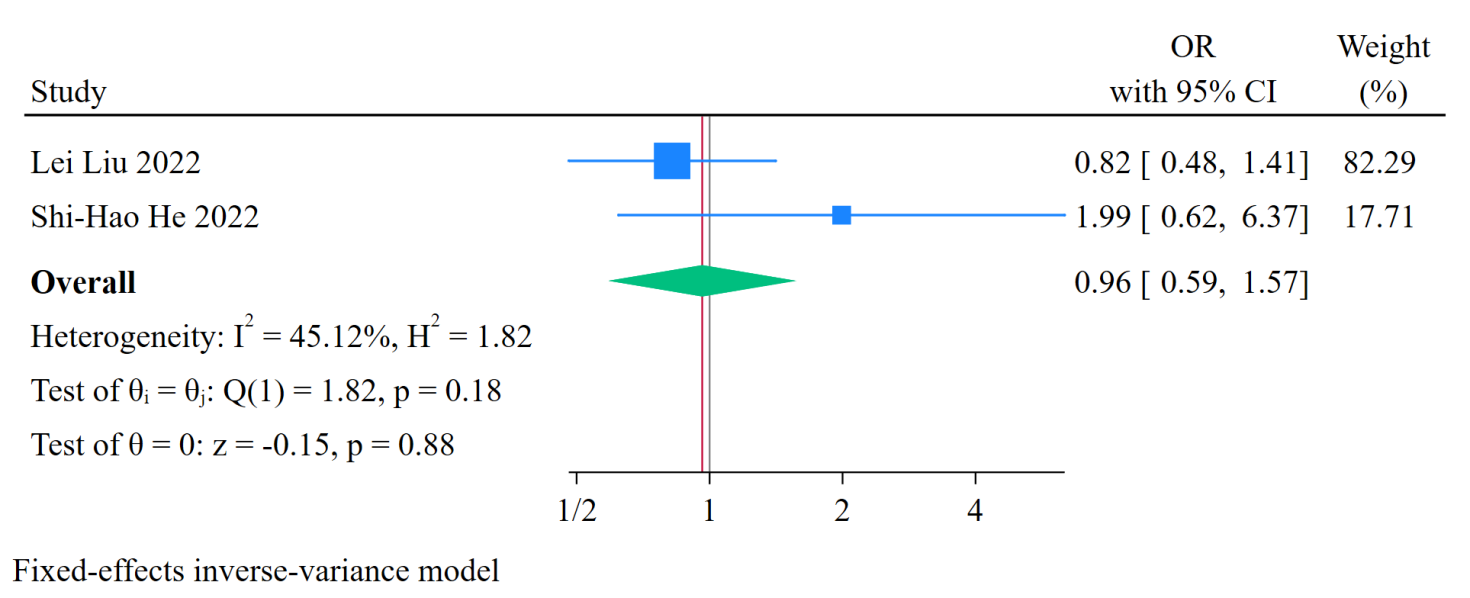


Forest plot for reticulation on HRCT as a risk factor in the progression of CTD-ILD

### **Pulmonary hypertension**


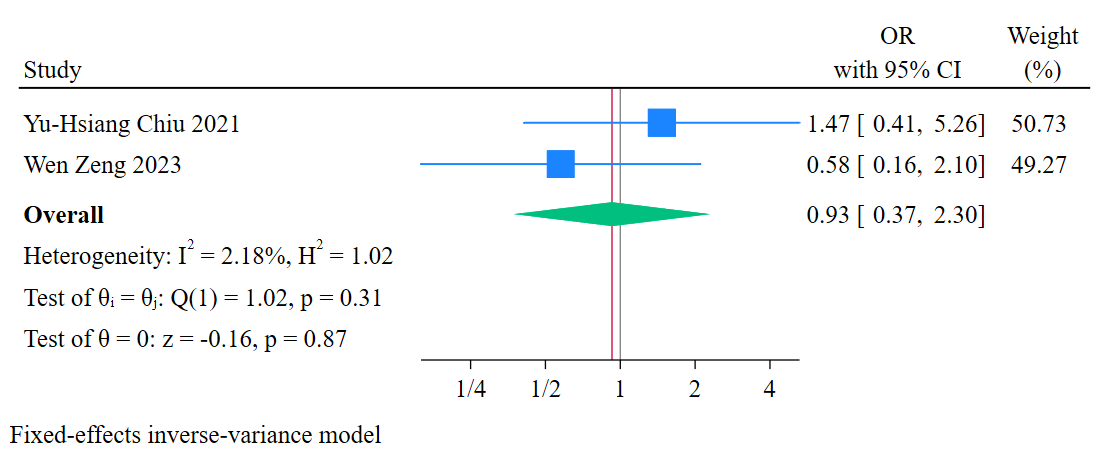


Forest plot for pulmonary hypertension as a risk factor in the progression of CTD-ILD

### **Congestive heart failure**


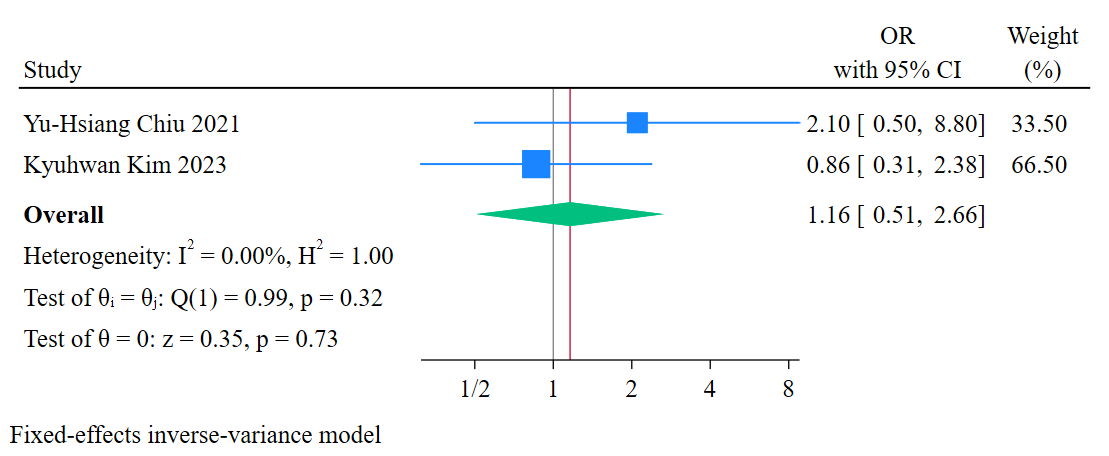


Forest plot for congestive heart failure as a risk factor in the progression of CTD-ILD

### **CYC**


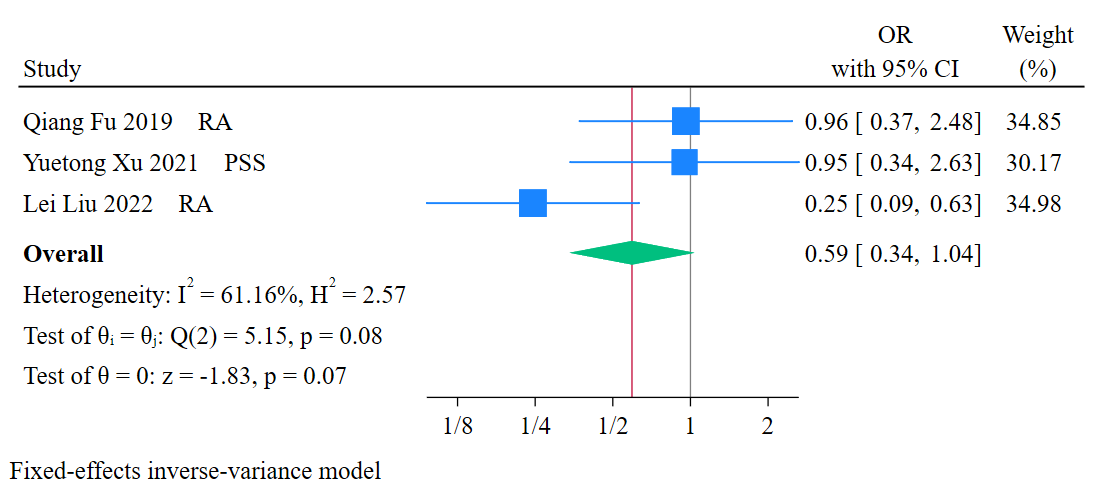


Forest plot for CYC as a risk factor in the progression of CTD-ILD

### **IS**


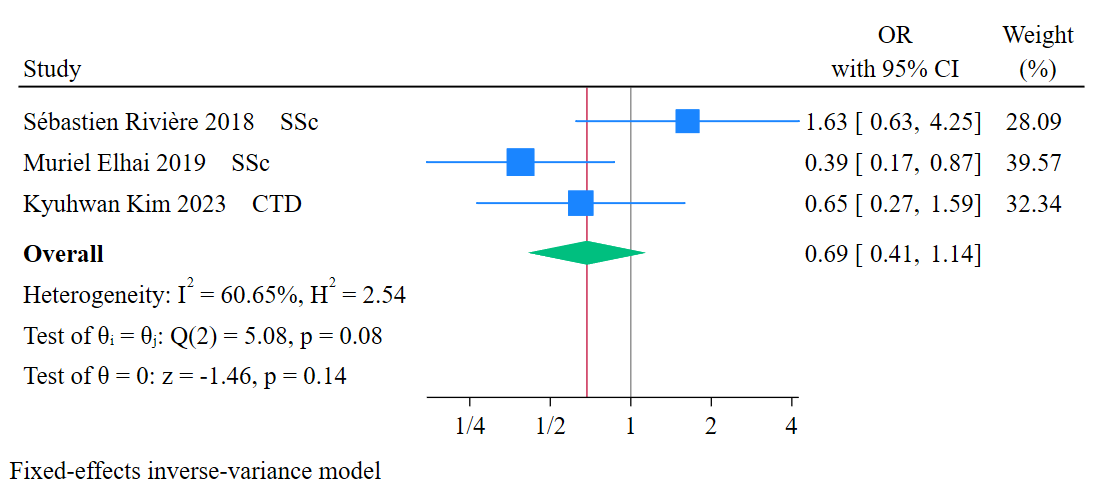


Forest plot for IS as a risk factor in the progression of CTD-ILD

### **LEF**


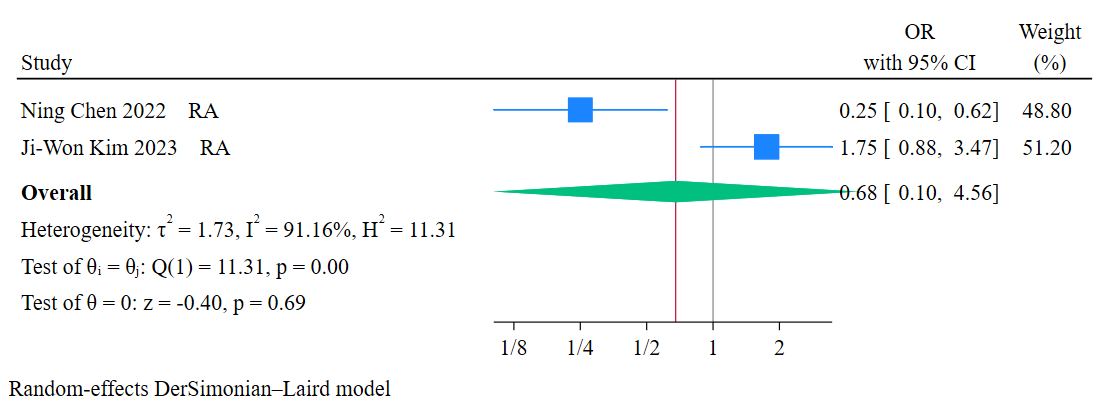


Forest plot for LEF as a risk factor in the progression of CTD-ILD

### **MTX**


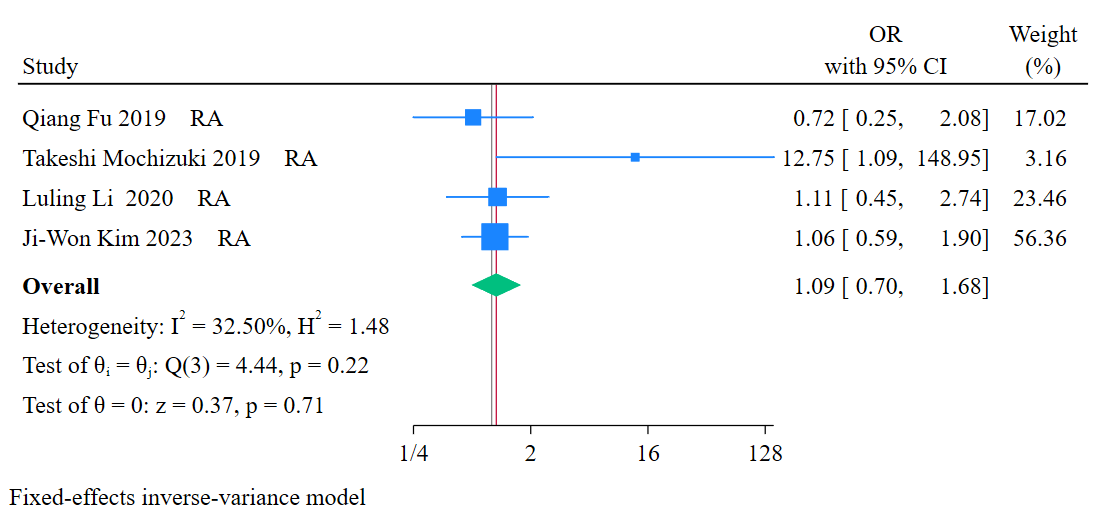


Forest plot for MTX as a risk factor in the progression of CTD-ILD

### **Steroids**


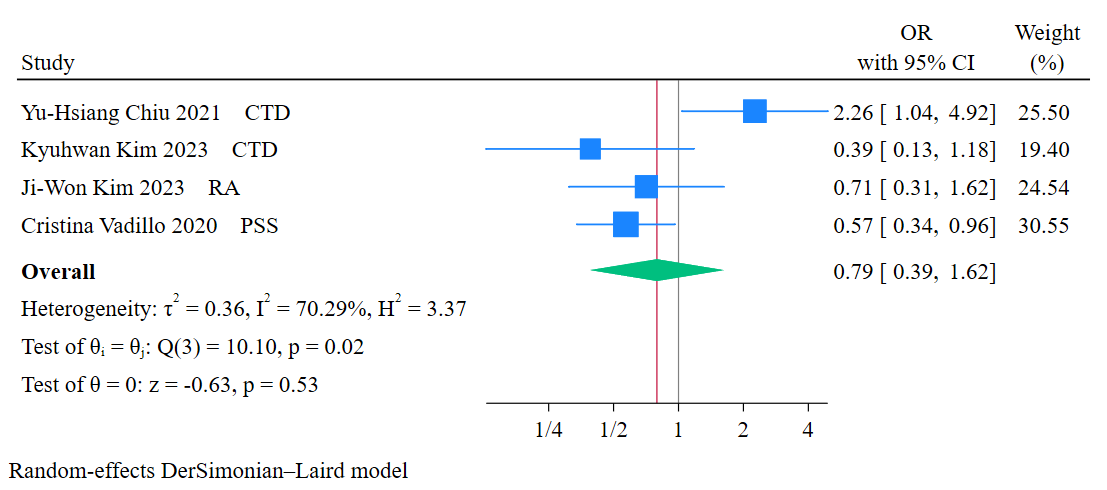


Forest plot for steroids as a risk factor in the progression of CTD-ILD


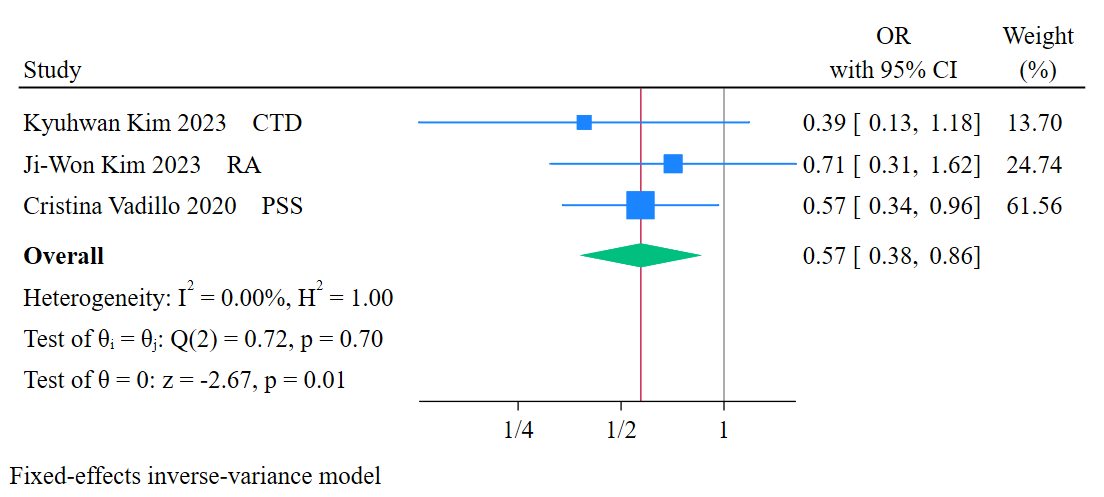


Forest plot for steroids as a risk factor in the progression of CTD-ILD after excluding one study

## 5.Meta-analysis results of the acute exacerbation of CTD-ILD.

### **FVC**


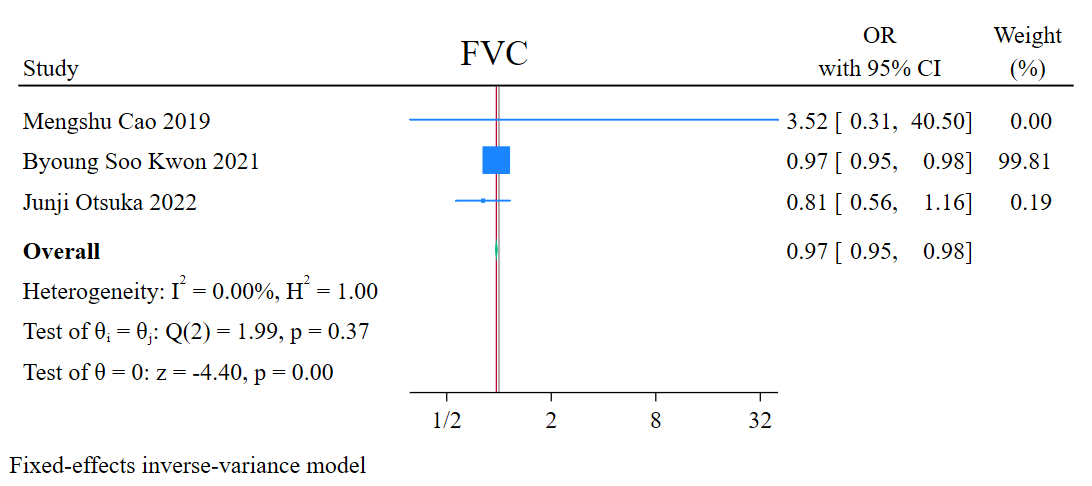


Forest plot for FVC as a risk factor in the AE of CTD-ILD

### **UIP**


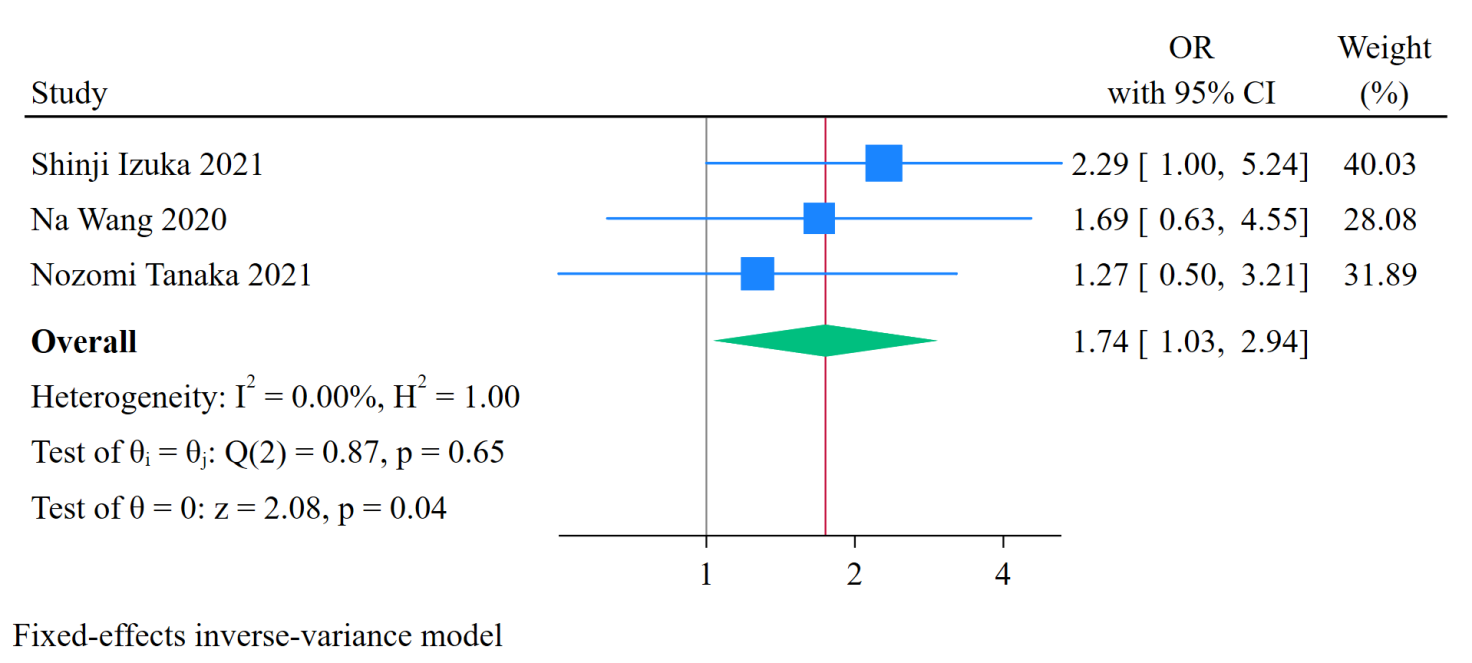


Forest plot for UIP as a risk factor in the AE of CTD-ILD

### **Smoking history**


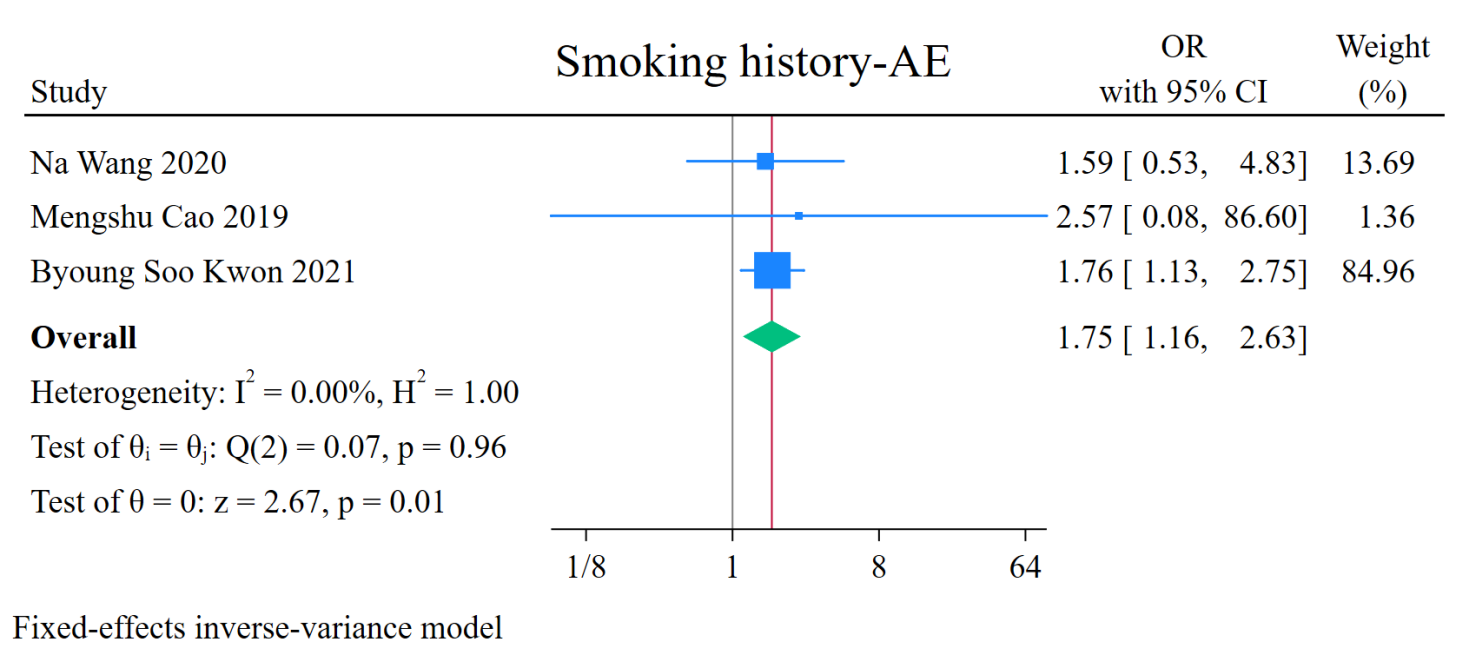


Forest plot for smoking history as a risk factor in the AE of CTD-ILD

### **Age**


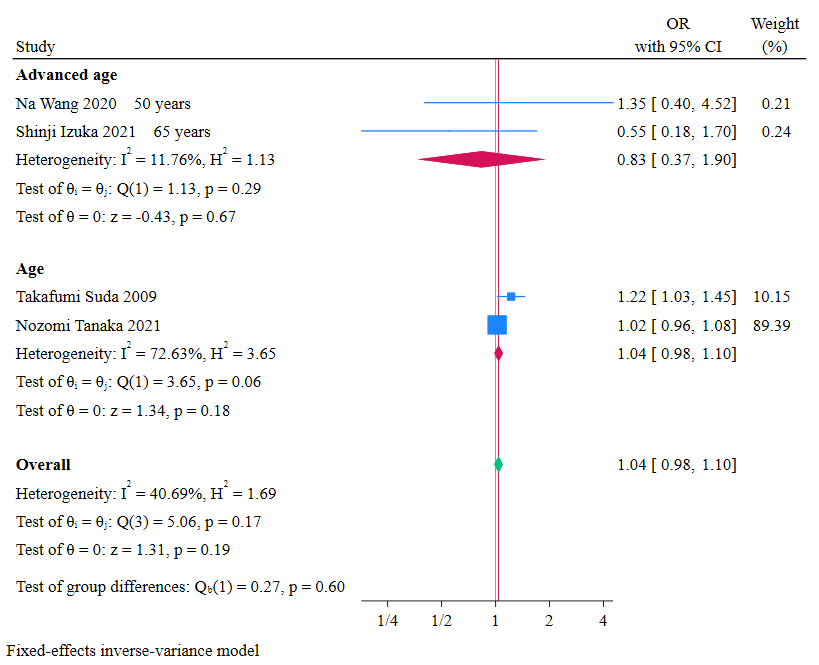


Forest plot for age as a risk factor in the AE of CTD-ILD

### **MTX**


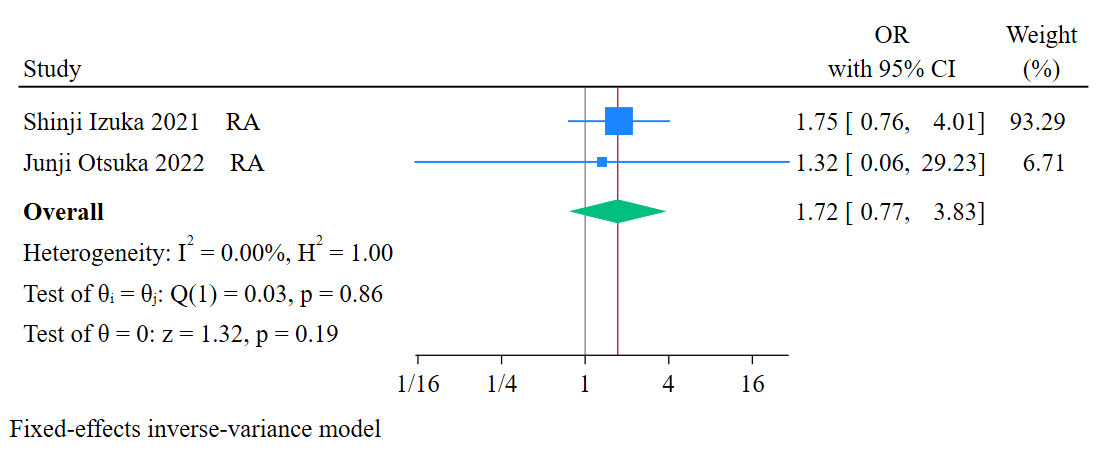


### **Steroids**


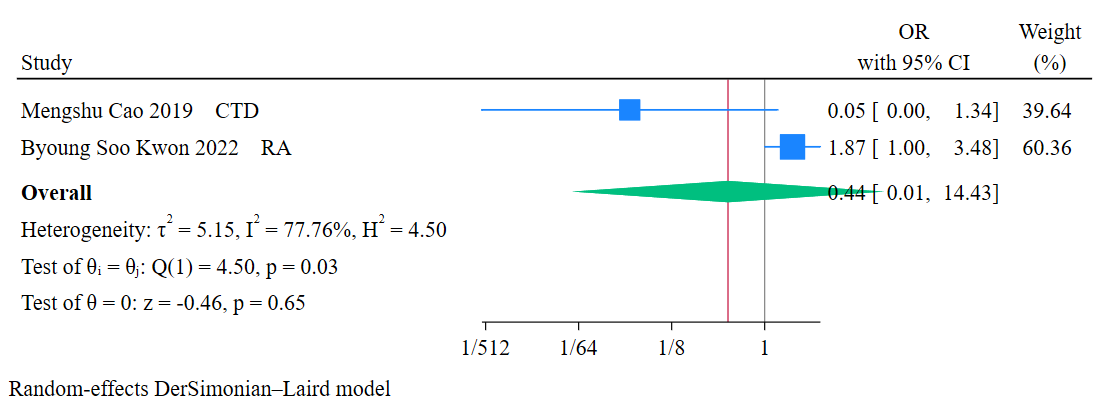


## 6.Meta-analysis results of the rapidly progressive of CTD-ILD

### Disease duration


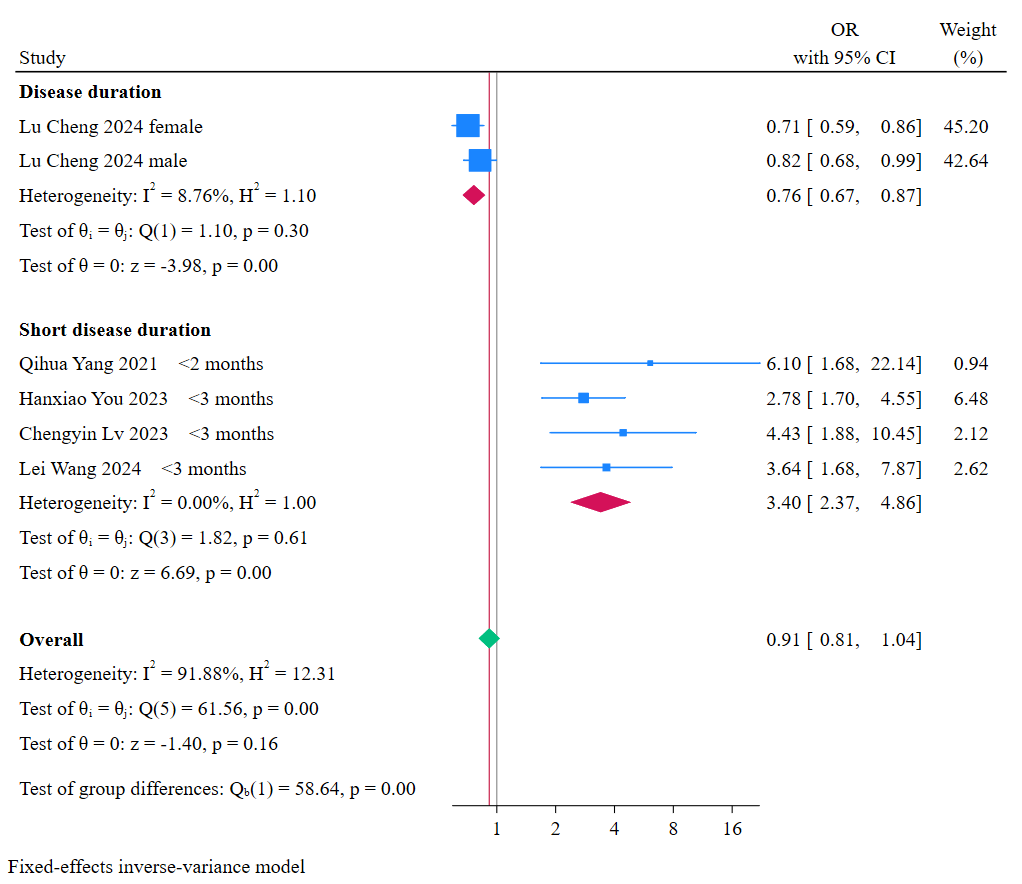


### CRP


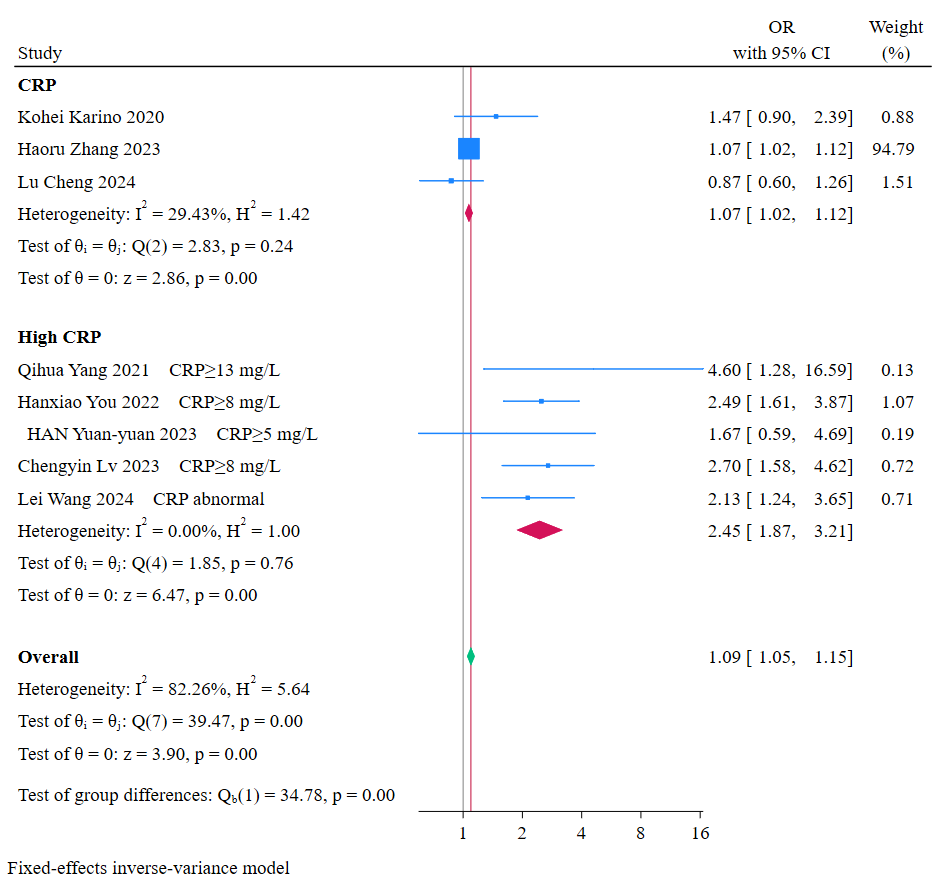


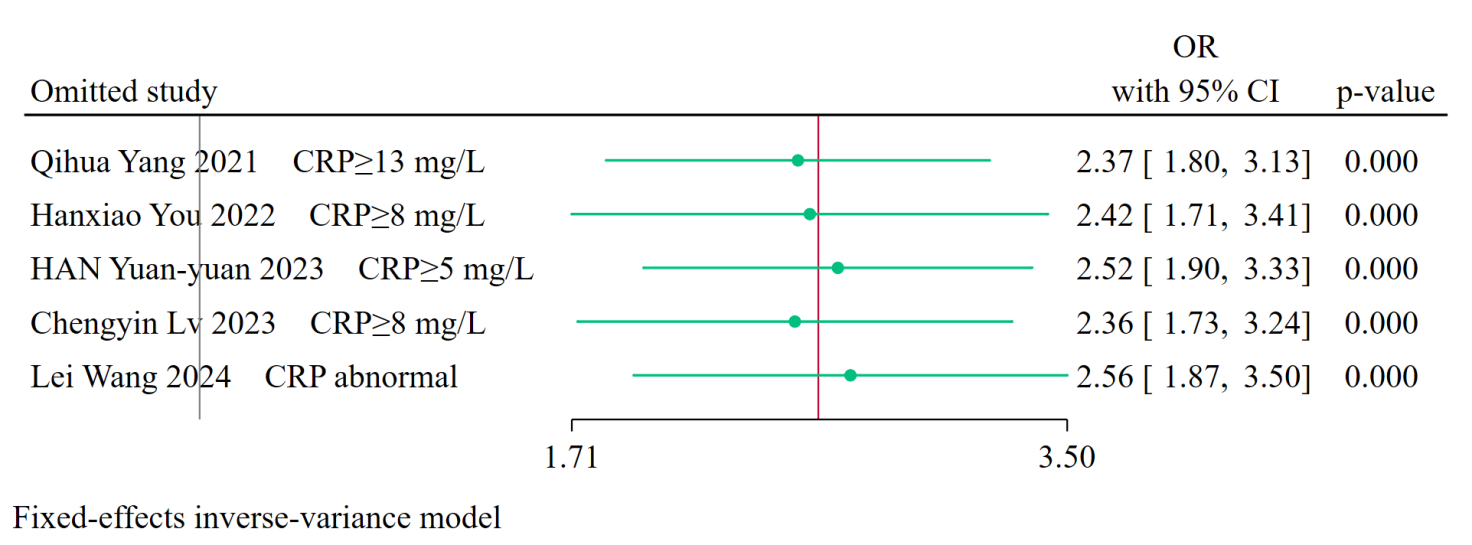


### Ro-52


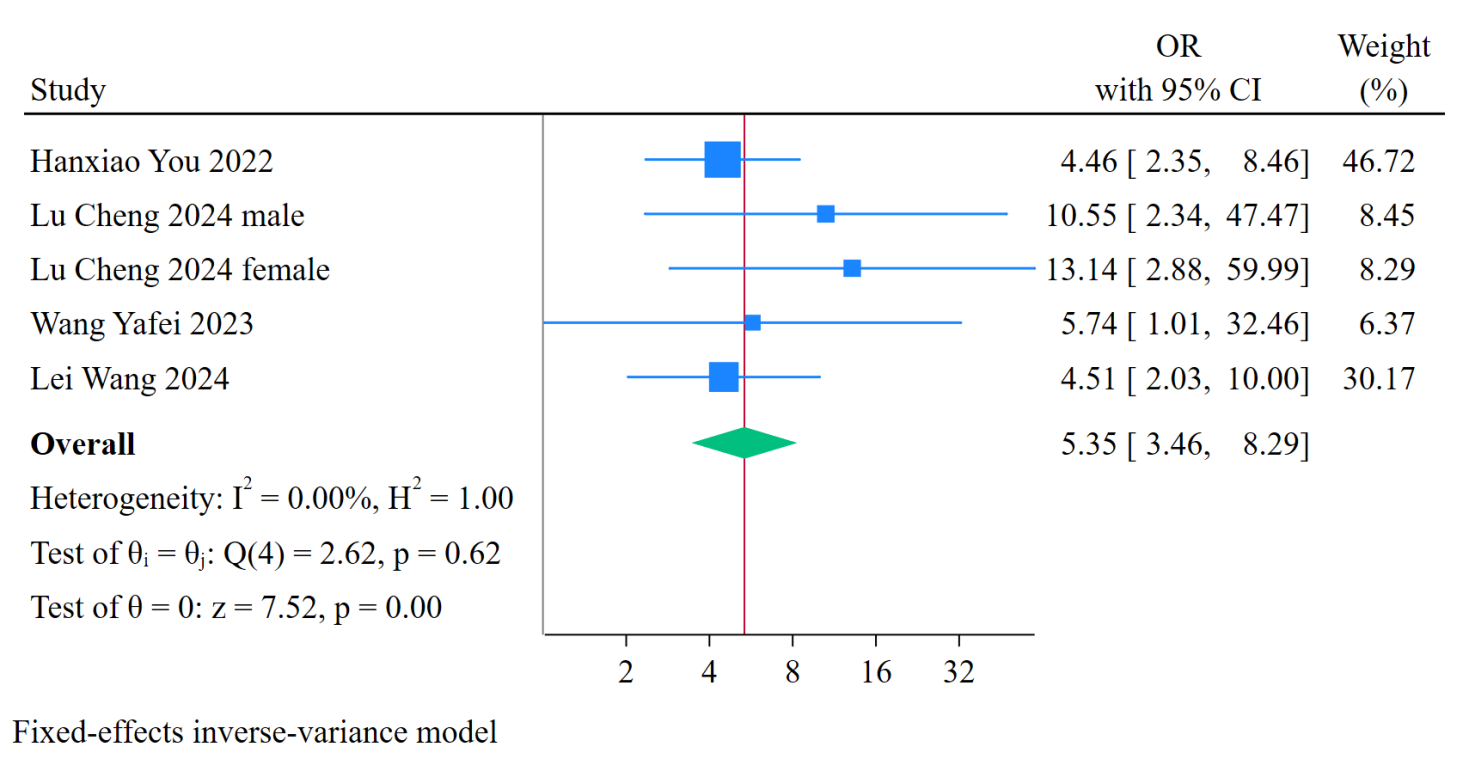


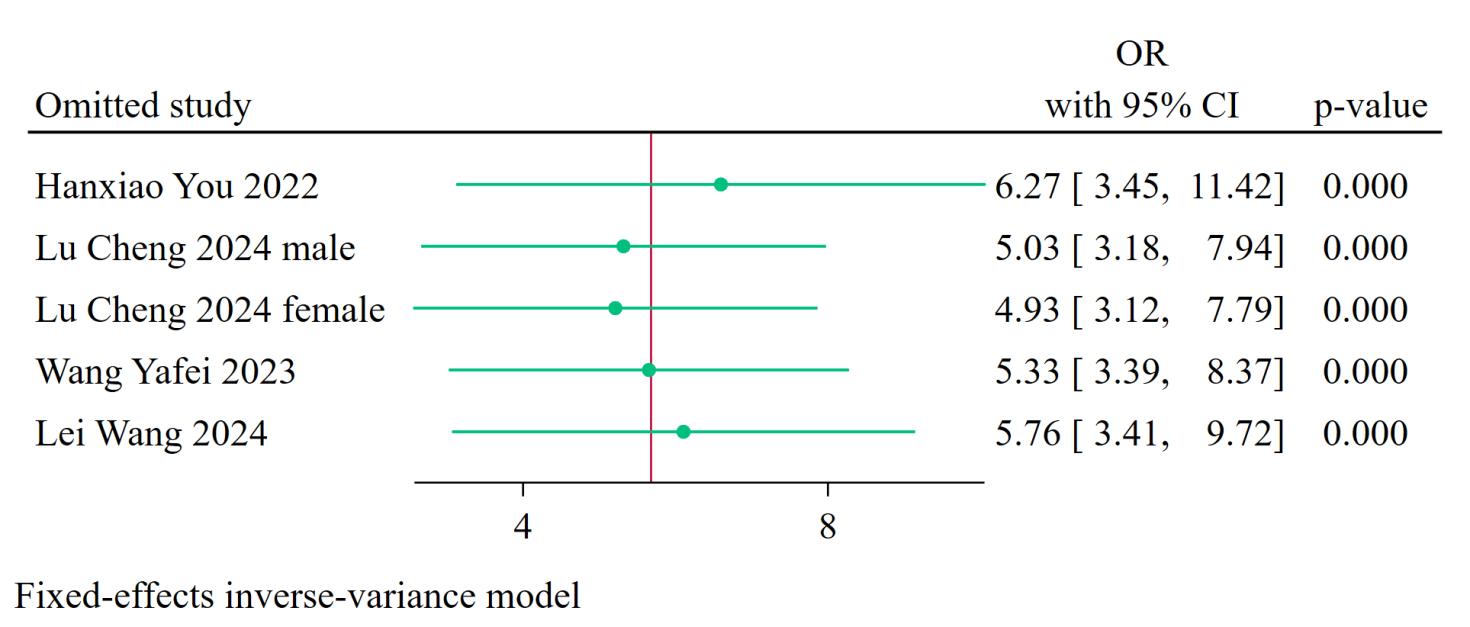


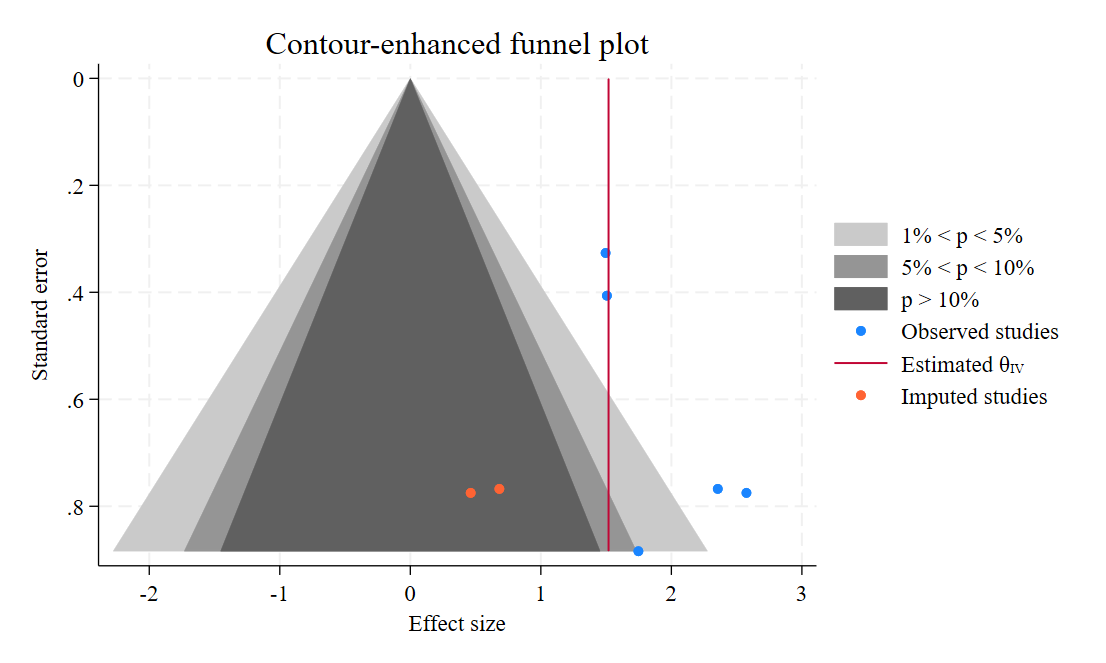


### MDA5


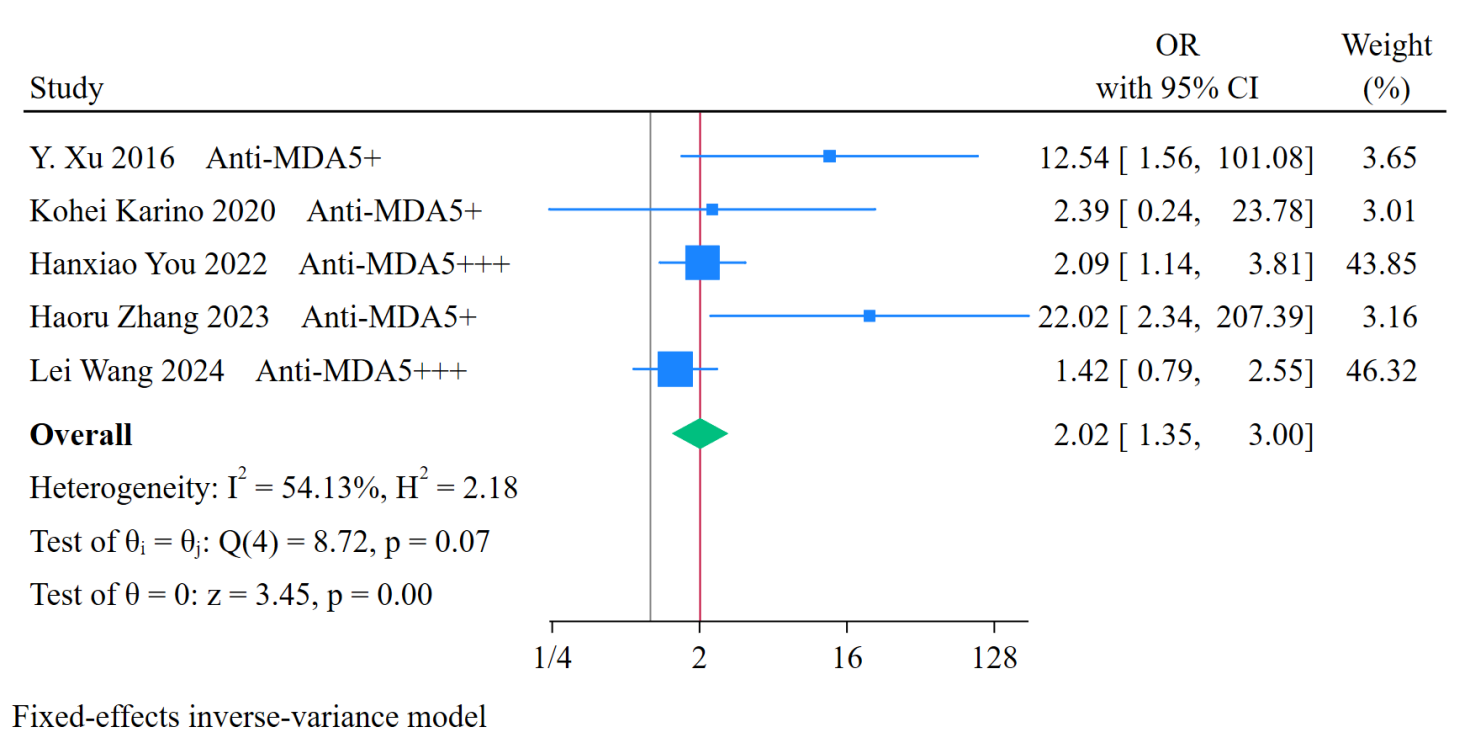


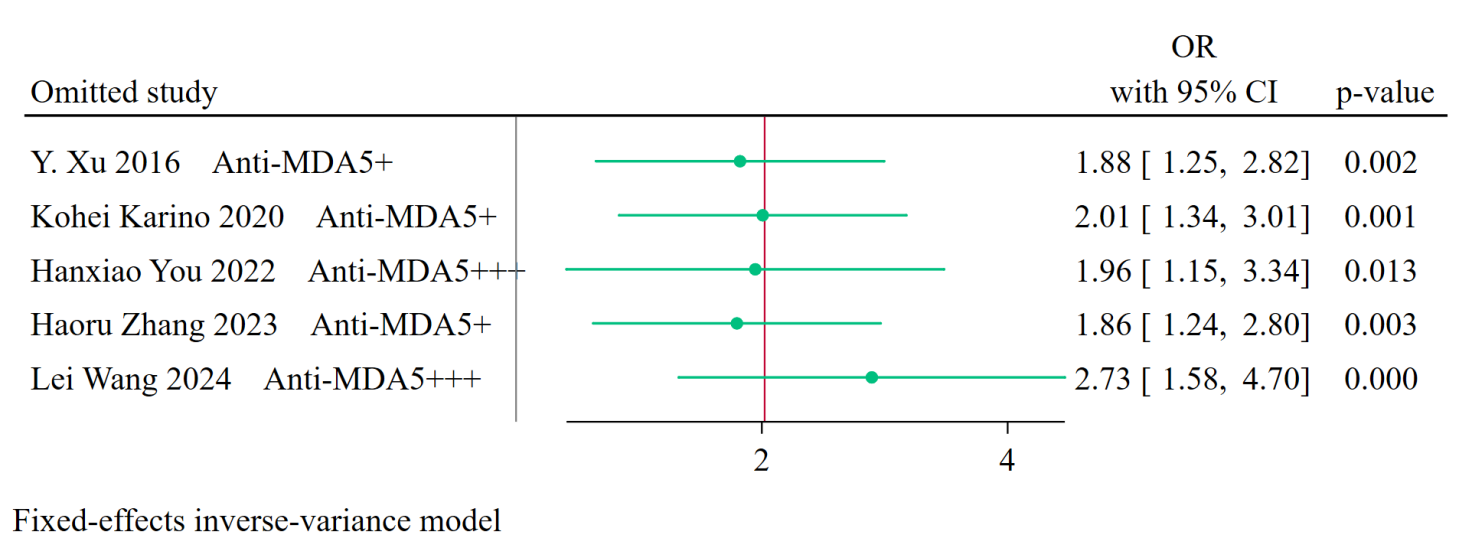


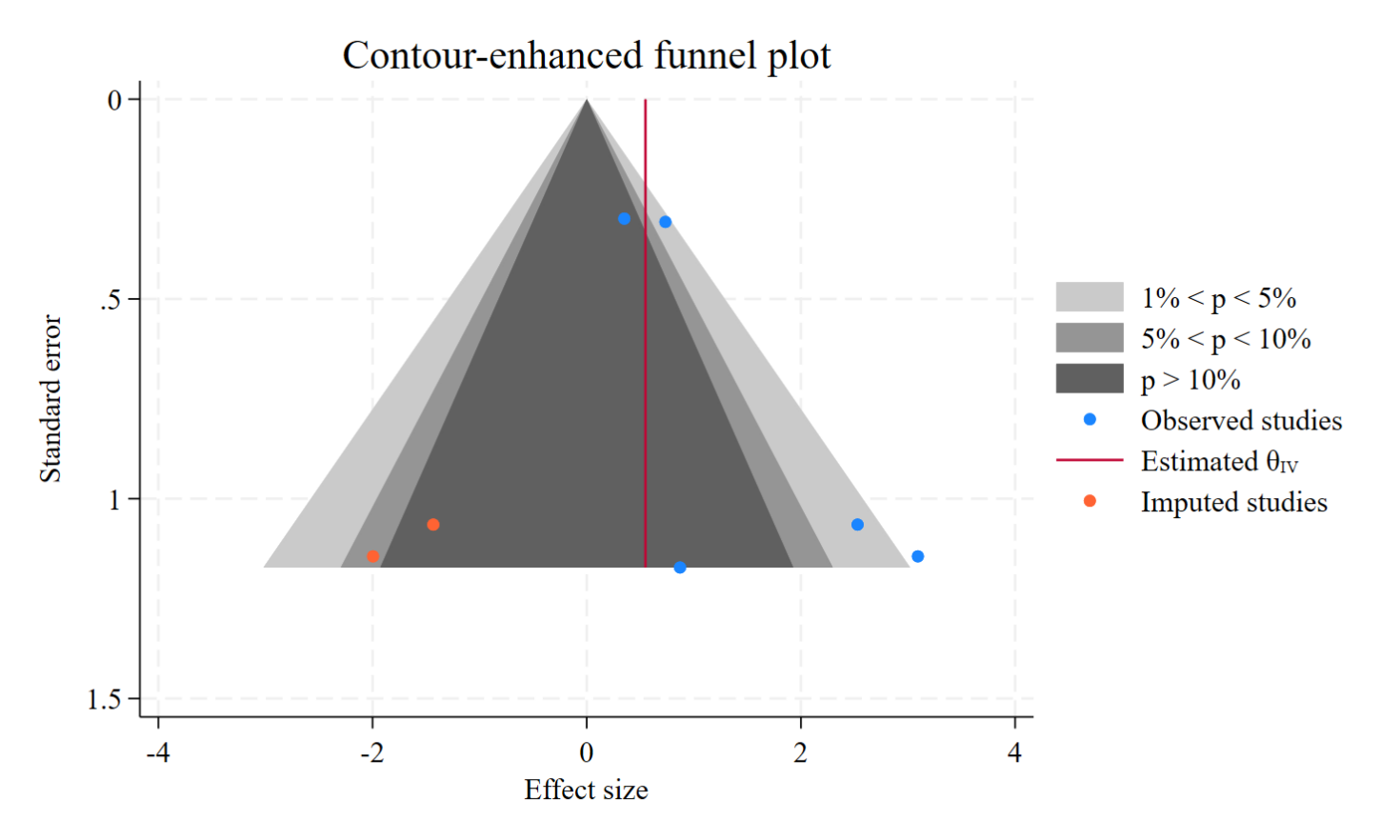


### Age


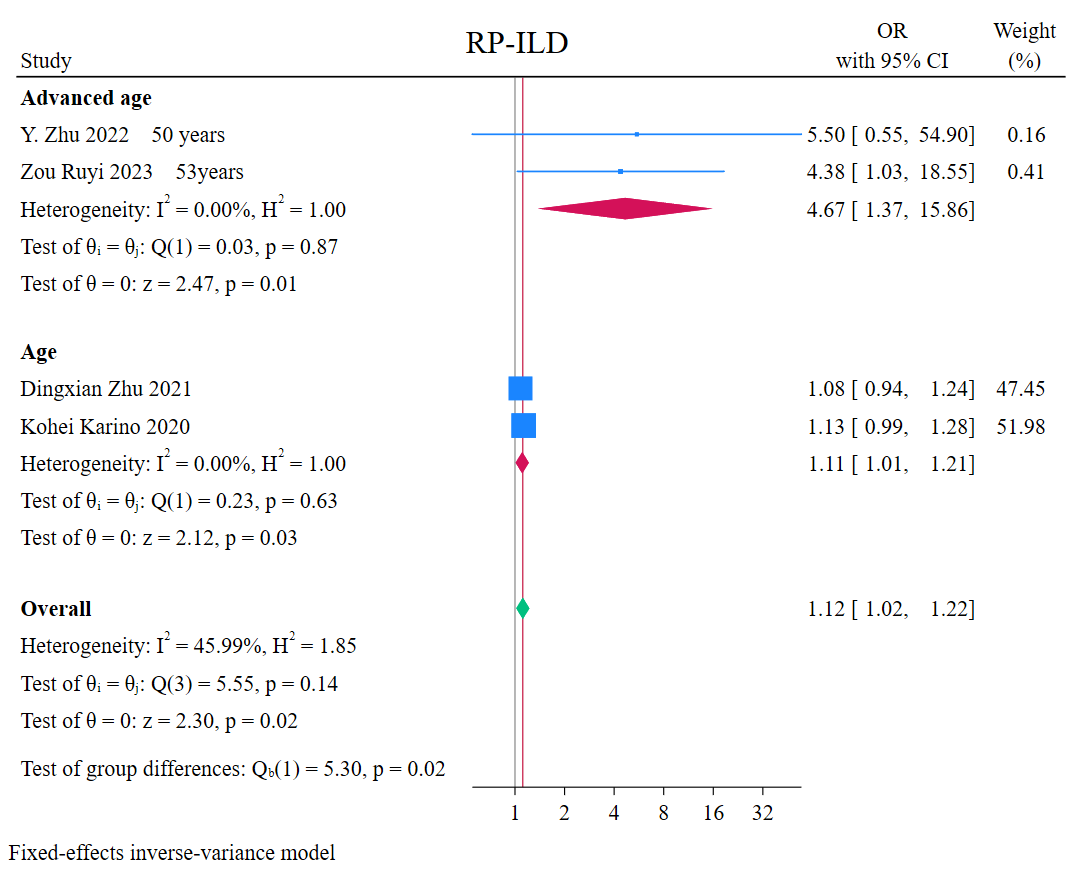


### Male


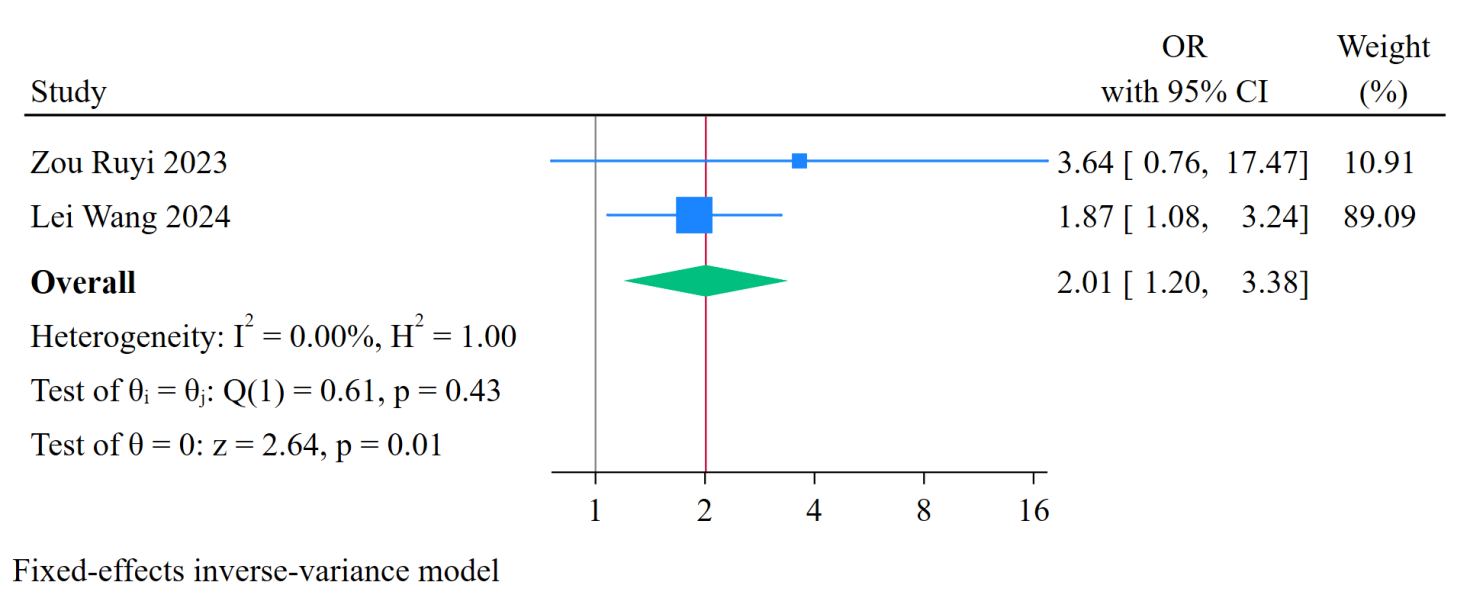


### Lymphocytes


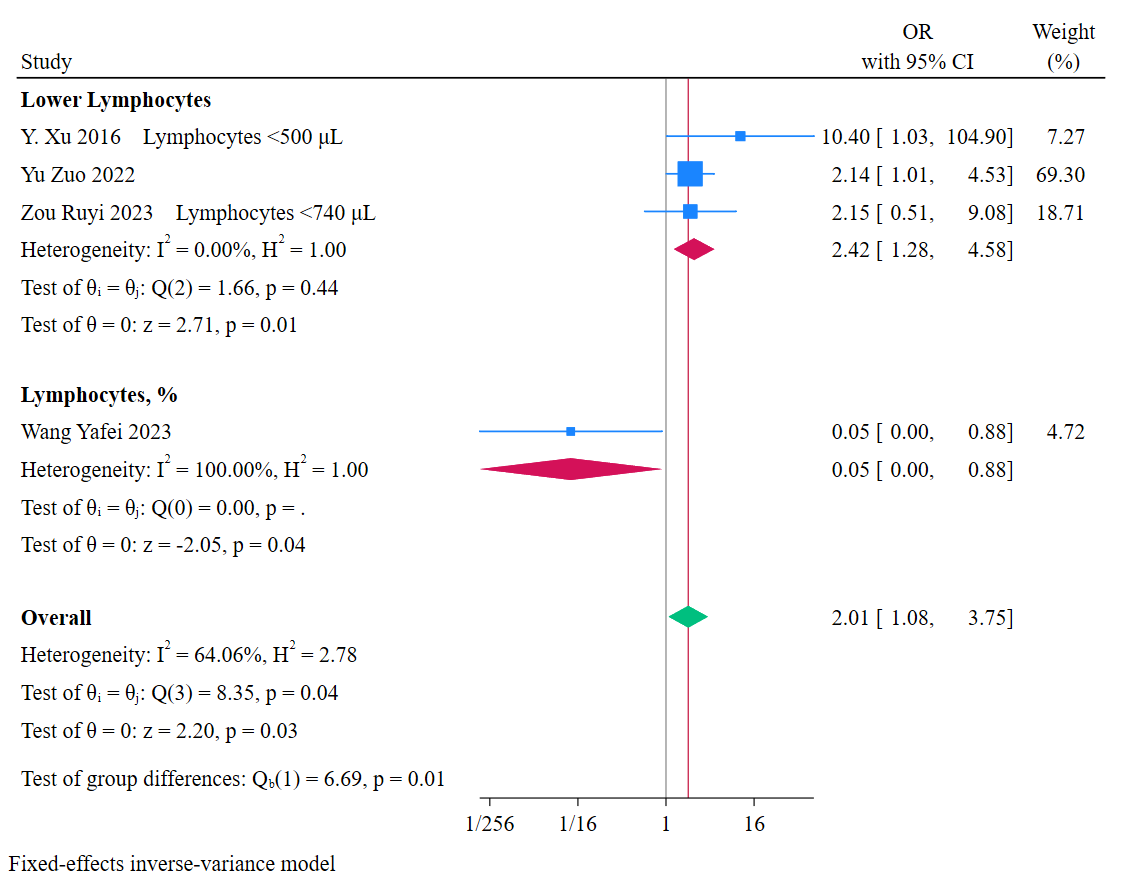


### LDH


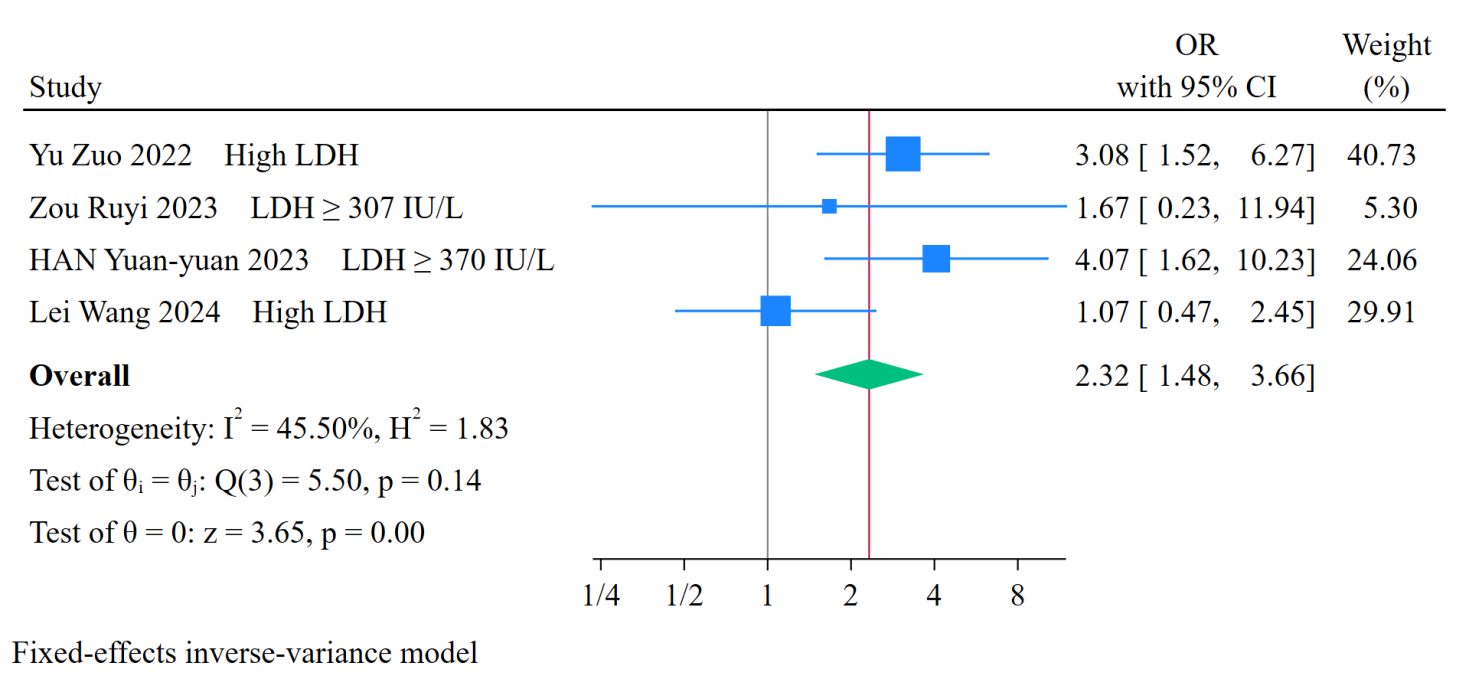


### SF


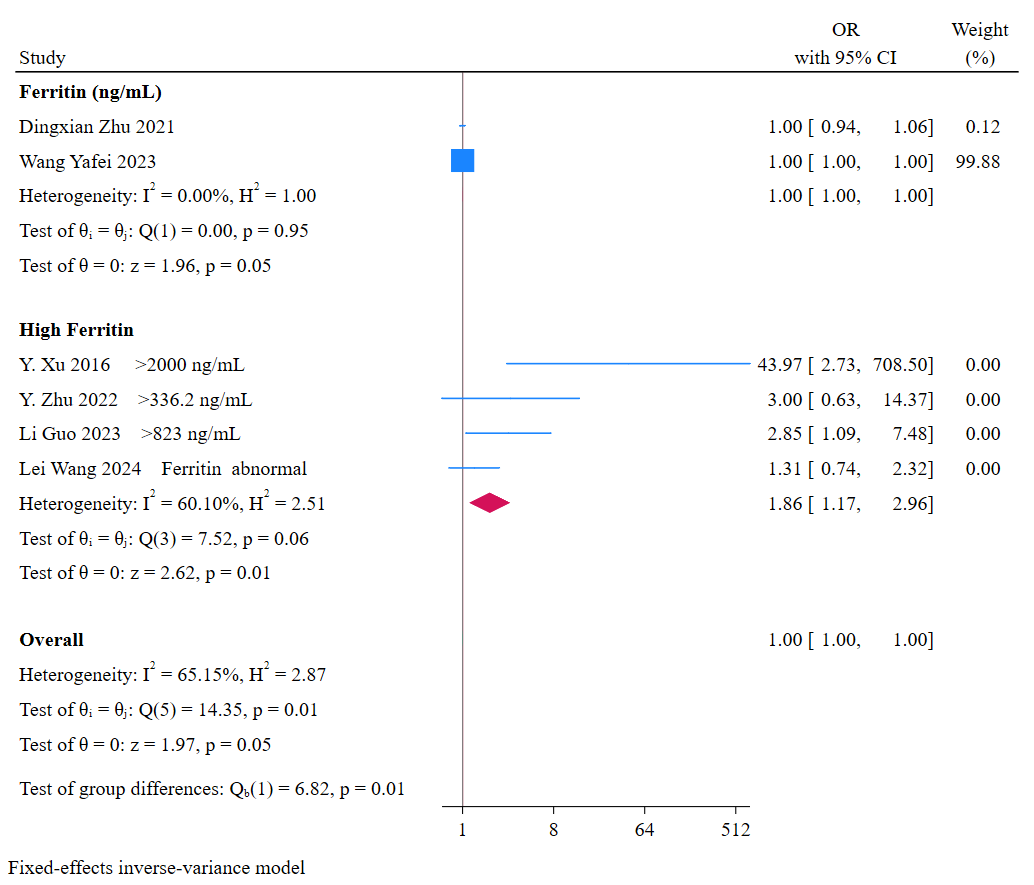


### CEA


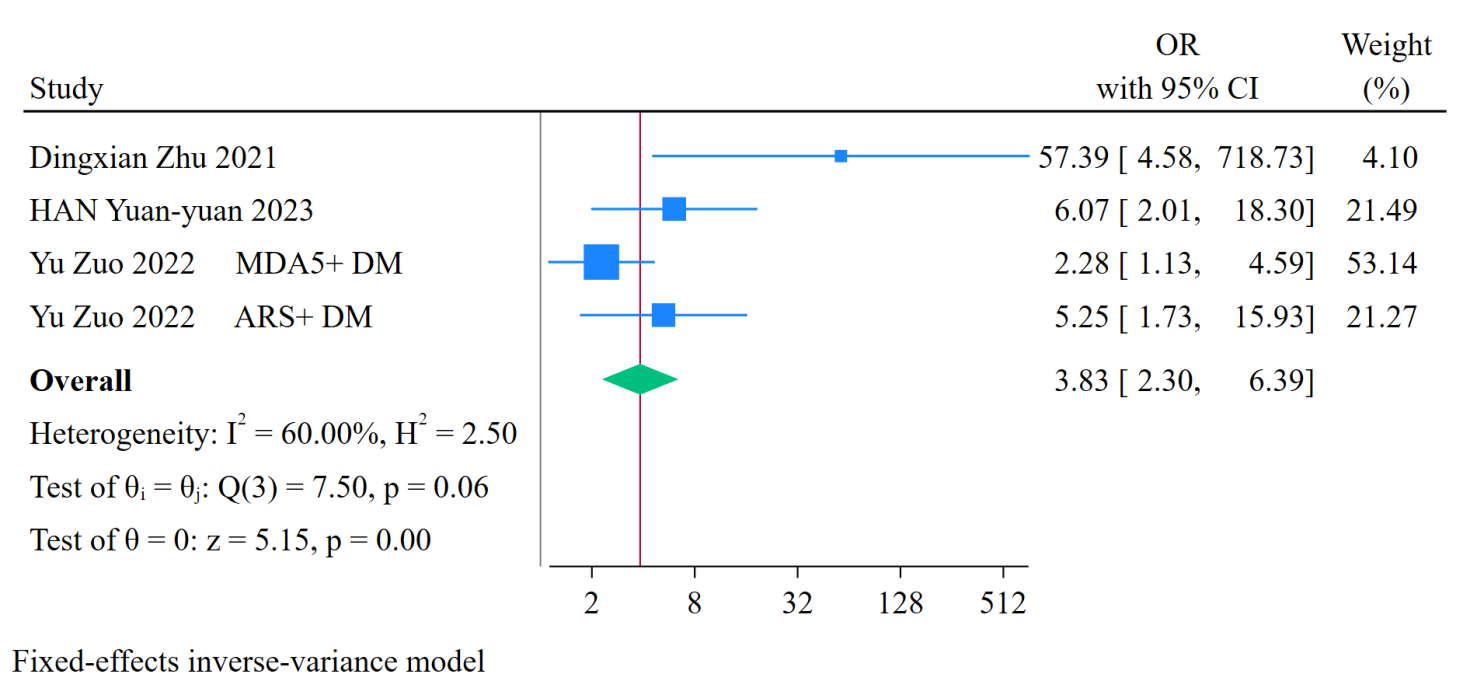


### Fever


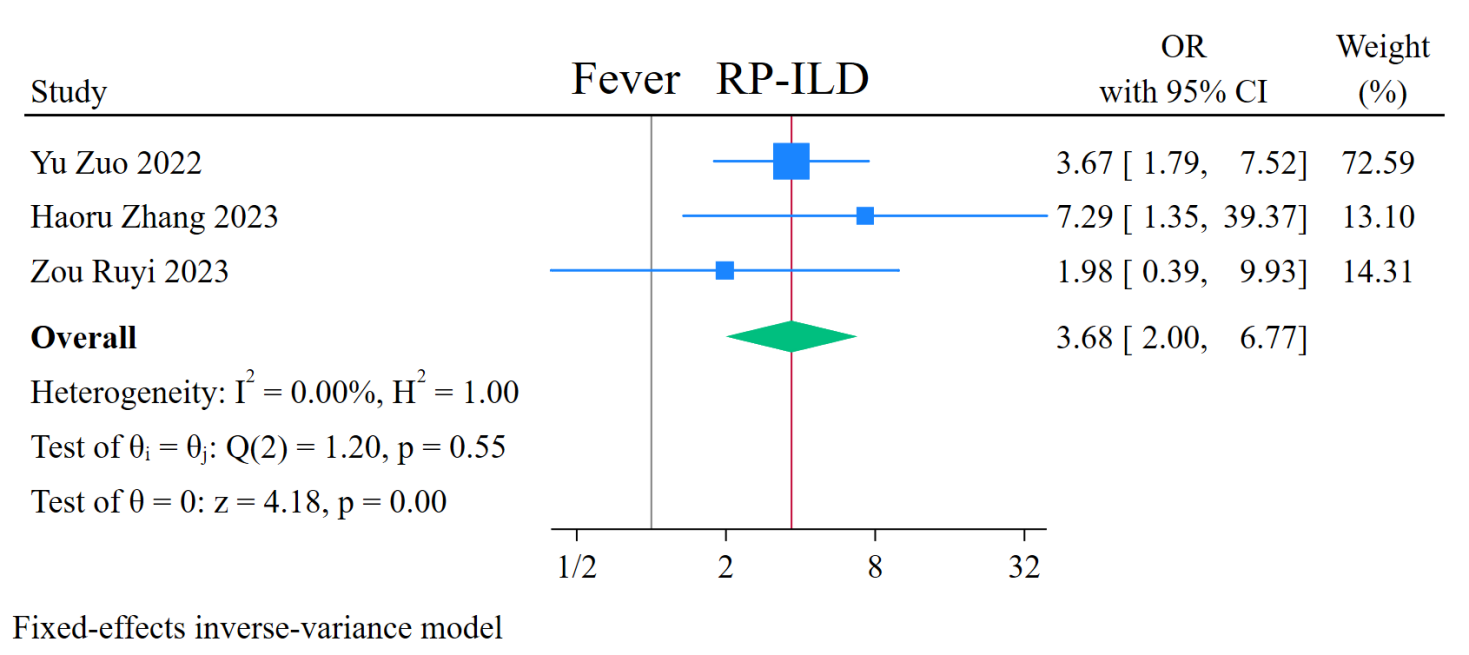


### Arthritis


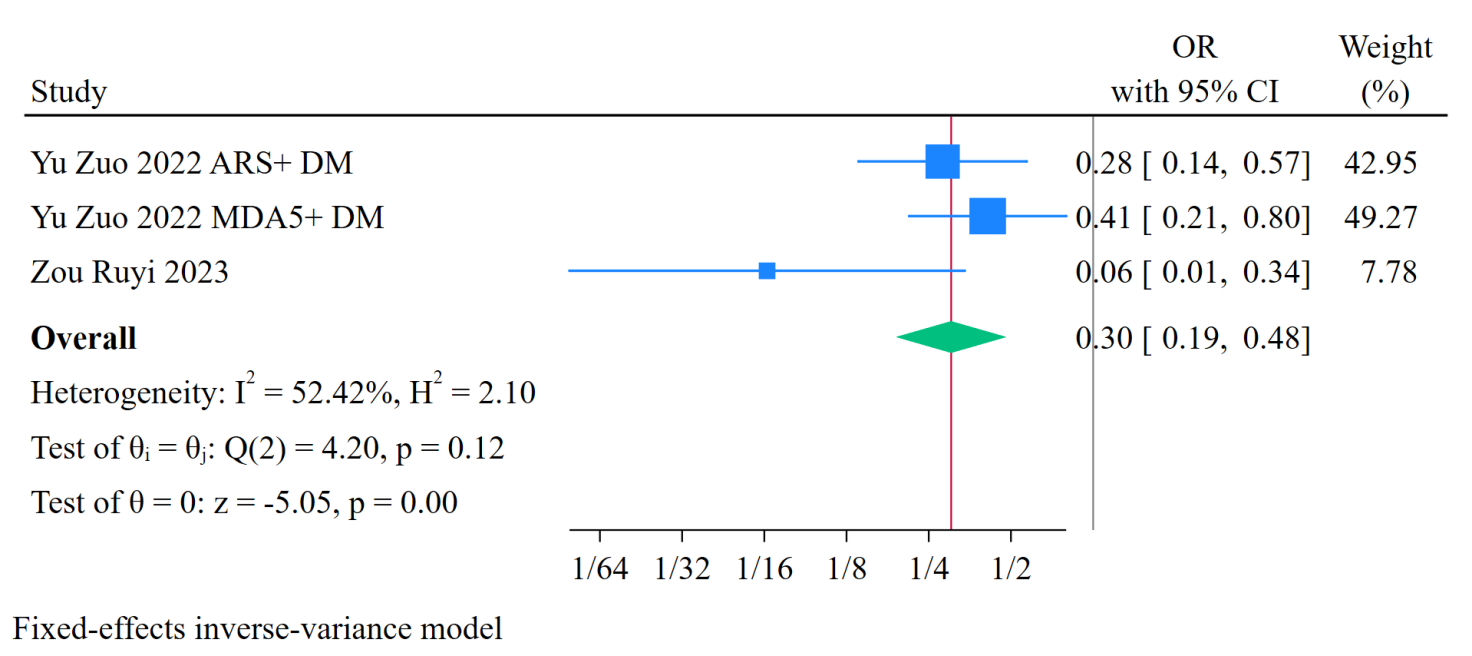


### Muscle weakness


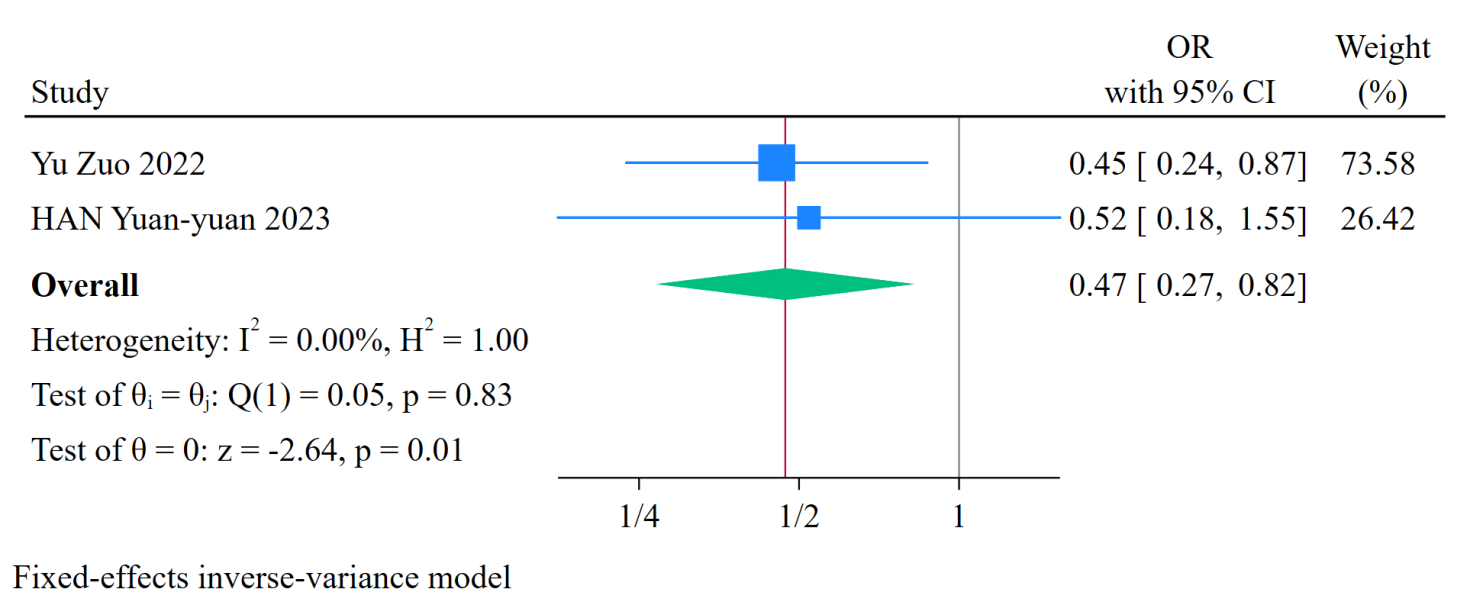


### AST


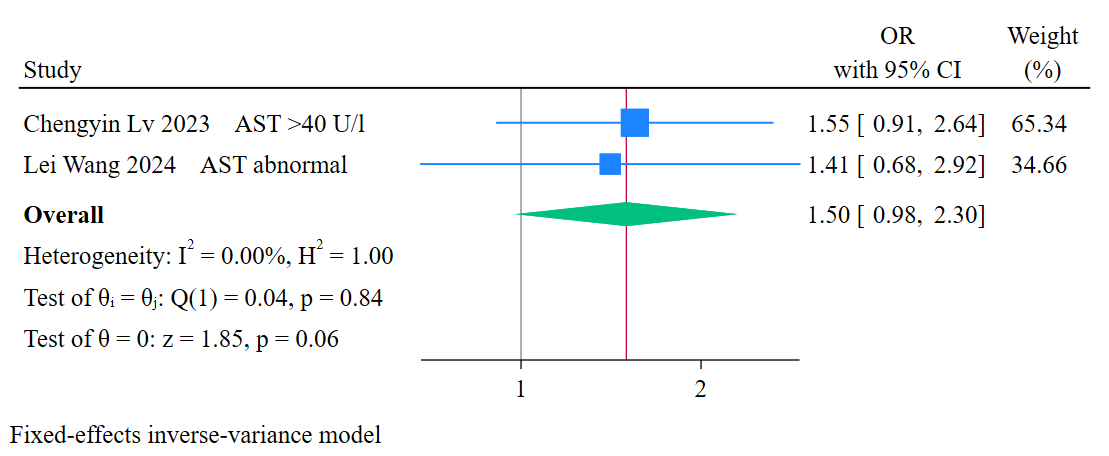


### ALT


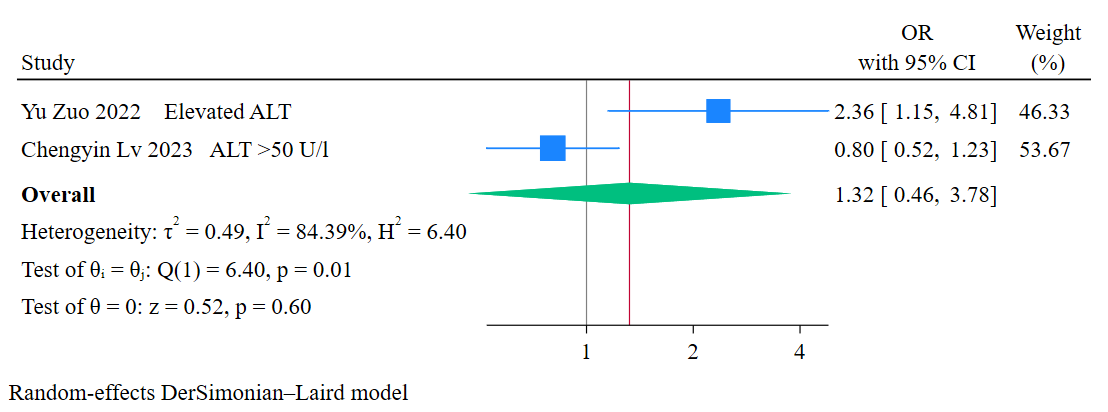


## 7.**Supplementary Table 3:** Summary of risk factors not included in the meta-analysis

| **Type** | **Risk factors** | **Type of CTD** | **OR/RR/HR** | **Types of effect sizes** |
| --- | --- | --- | --- | --- |
| **Progression** | Ventilatory insufficiency due to striated muscle weakness | IIM-ASS | 10.95 (1.46, 82.30) | OR |
|  | Long disease duration | SSc | 9.20 (1.20, 7.80) | RR |
|  | IL-6 (pg/mL) | RA | 1.04 (1.00, 1.08) | OR |
|  | Anti-PL-7 antibody positivity | IIM | 39.48 (3.57, 1639) | OR |
|  | CC16> 33 ng/ml | SSc | 2.90 (1.20–6.75) | HR |
|  | Serum creatinine |  | 1.00 (0.99–1.02) |  |
|  | Anti-Ro-52 positivity | MCTD | 3.50 (1.20, 10.20) | HR |
|  | Anti-RNP value (per 50 U/L increase) |  | 1.50 (1.10, 2.00) |  |
|  | ILD of TLV |  | 1.10 (1.00, 1.10) |  |
|  | CX3CL1 (pg/ul) | SSc | 1.30 (1.12, 1.52) | OR |
|  | Age at onset (years) |  | 1.07 (1.04, 1.10) |  |
|  | CD3+CD4+ cell counts | IIM-ASS | 1.00 (1.00, 1.00) | OR |
|  | Fever at presentation |  | 12.27 (2.17, 69.46) |  |
|  | ILD occurs within 10 years of RA diagnosed | RA | 0.47(0.18, 1.22) | OR |
|  | CCL18＞56.6 ng/ml | SSc | 2.90 (1.25, 6.74) | OR |
|  | Overall extent of lung abnormalities | RA | 0.98 (0.95, 1.02) | OR |
|  | Respiratory symptoms | RA | 1.12 (0.47, 2.66) | OR |
|  | Emphysema | RA | 0.37 (0.19, 0.70) | OR |
|  | Lag time to ILD diagnosis |  | 1.03 (1.01, 1.04) |  |
|  | Decreased C3(%) | PSS | 2.90 (0.95, 8.85) | OR |
|  | Raynaud's syndrome(%) |  | 2.01 (0.40, 10.04) |  |
|  | Hypoproteinemia |  | 1.02 (0.35, 3.01) |  |
|  | Coronary artery disease | CTD | 1.09 (0.31, 3.81) | OR |
|  | Diabetes mellitus |  | 4.52 (1.10, 18.51) |  |
|  | Anti-dsDNA |  | 0.16 (0.03, 0.78) |  |
|  | Anti-Scl-PM |  | 0.64 (0.16, 2.48) |  |
|  | Anti-Ro/SSA |  | 0.61 (0.28, 1.33) |  |
|  | Obesity |  | 0.16 (0.03, 0.85) |  |
|  | RA VS SSC, IM | CTD | 1.90 (1.32, 2.73) | HR |
|  | Anti-topoisomerase-I positive | SSc | 1.58 (0.85, 2.92) | HR |
|  | HRCT scores at baseline | RA | 1.01 (0.98, 1.03) | HR |
|  | Interlobular septal thickening | RA | 0.97 (0.89, 1.07) | OR |
|  | HAQ-DI |  | 1.36 (0.63, 2.93) |  |
|  | GGO |  | 0.90 (0.79, 1.03) |  |
|  | DAS28-ESR |  | 1.88 (1.07, 3.33) |  |
|  | Initial glucocorticoid dose, mg/d | IIM-ASS | 0.96 (0.92, 1.00) | OR |
|  | CD3-CD19+, cells/ul |  | 1.00 (1.00, 1.00) |  |
|  | Sicca onset | PSS | 0.25 (0.07, 0.96) | OR |
|  | Baseline Albumin (g/dL) | CTD | 0.61 (0.33, 1.12) | HR |
|  | Digital ulcers | MCTD | 16.7 (2.80, 100) | HR |
|  | Follow-up duration (years) | PSS | 1.40 (1.06, 1.87) | OR |
|  | Time interval between CTD and ILD diagnosis | CTD | 0.95 (0.88, 1.03) | OR |
|  | ANCA |  | 2.05 (0.47, 8.83) |  |
|  | Antifibrotics |  | 1.32 (0.31, 5.61) |  |
|  | Albumin level at 3rd month | PSS | 0.02 (0.00, 0.97) | OR |
|  | IgG level change during first 3 months of ILD diagnosis |  | 1.00 (0.94, 1.07) |  |
|  | Cough | SSc | 0.91 (0.23, 3.60) | OR |
|  | Chest tightness and pain |  | 0.50 (0.09, 2.84) |  |
|  | TAC | RA | 0.94 (0.52, 1.72) | HR |
|  | Biological or targeted synthetic DMARD |  | 0.88 (0.48, 1.63) |  |
| **AE** | RA VS CTD | CTD | 2.47 (0.93, 11.21) | HR |
|  | Anticentromere | SSc | 0.31 (0.07, 1.40) | HR |
|  | Overlap with polymyositis/dermatomyositis positive |  | 6.97 (1.81, 26.93) |  |
|  | Corticosteroids reduction or discontinuation | CTD | 0.70 (0.39, 1.28) | HR |
|  | Pulmonary hypertension |  | 1.35 (0.97, 1.87) |  |
|  | MUC5B | RA | 2.31 (0.95, 5.62) | HR |
|  | Age at onset of RA |  | 5.25 (0.49, 56.36) |  |
|  | ACPA titer>500 |  | 0.72 (0.26, 1.99) |  |
|  | Male |  | 0.51 (0.06, 4.42) |  |
|  | RA duration |  | 1.16 (0.17, 8.07) |  |
|  | KL-6 before AE or the last visit | RA | 3.37 (1.16, 8.87) | HR |
|  | KL-6 levels at RA-ILD diagnosisa |  | 1.06 (0.92, 1.21) |  |
|  | 6MWT distance | RA | 1.00 (1.00, 1.00) | OR |
|  | Lowest SpO2 |  | 0.90 (0.86, 0.93) |  |
|  | ILD diagnosis preceding RA onset | RA | 3.11 (0.37, 25.80) | OR |
|  | Alb, g/dl |  | 0.09 (0.01, 0.73) |  |
|  | VC, %pred |  | 1.23 (0.87, 1.74) |  |
|  | HRCT, honeycombing |  | 0.78 (0.06, 10.46) |  |
| **RP-ILD** | Skin ulcerations | CADM | 17.37 (1.19,254.09) | OR |
|  | Myofascia/muscle | DM | 25.45 (1.71, 379.71) | OR |
|  | Total CT GGO score≥4 | IIM | 10.20 (3.80, 37.50) | OR |
|  | Elevated fet | IIM | 4.97 (1.97, 12.50) | OR |
|  | Decreased CD3+T |  | 2.56 (1.17, 5.61) |  |
|  | Decreased CD3+CD4+T |  | 2.80 (1.37, 5.73) |  |
|  | Decreased CD3+CD8+T |  | 2.18 (1.05, 4.50) |  |
|  | Elevated B2 |  | 3.17 (1.41, 7.13) |  |
|  | Elevated CA153 |  | 3.31 (1.50, 7.27) |  |
|  | Myalgia |  | 0.40 (0.20, 0.79) |  |
|  | Elevated CA125 |  | 2.79 (1.10, 7.11) |  |
|  | Elevated NSE |  | 4.86 (1.44, 16.37) |  |
|  | V sign | DM | 1.11 (0.33, 3.80) | OR |
|  | Shawl sign |  | 0.55 (0.13, 2.34) |  |
|  | Prednisolone ≥ 67.5 (mg/d) | DM | 29.82 (9.04, 124.70) | OR |
|  | WDFY4 rs7919656 |  | 4.98 (1.59, 17.19) |  |
|  | FVC % predicted < 50% |  | 9.63 (4.12, 25.16) |  |
|  | DLCO % predicted < 30% |  | 2.76 (1.45, 5.45) |  |
|  | RF-IgA | DM | 2.55 (1.23, 5.32) | OR |
|  | oxygenation index | DM | 0.98, (0.97, 0.99) | OR |
|  | Heliotrope rash |  | 1.42, (0.32, 6.32) |  |
|  | Neutrophil/lymphocyte ratio (≥4) | DM | 1.89 (0.61, 5.80) | OR |
|  | SpO2<90% | DM | 23.61 (2.81, 198.29) | OR |
